# Novel strategies to improve chicken performance and welfare by unveiling host-microbiota interactions through hologenomics

**DOI:** 10.3389/fphys.2022.884925

**Published:** 2022-09-06

**Authors:** Núria Tous, Sofia Marcos, Farshad Goodarzi Boroojeni, Ana Pérez de Rozas, Jürgen Zentek, Andone Estonba, Dorthe Sandvang, M. Thomas P. Gilbert, Enric Esteve-Garcia, Robert Finn, Antton Alberdi, Joan Tarradas

**Affiliations:** ^1^ Animal Nutrition, Institute of Agrifood Research and Technology (IRTA), Constantí, Spain; ^2^ Applied Genomics and Bioinformatics, University of the Basque Country (UPV/EHU, Bilbao, Spain; ^3^ Institute of Animal Nutrition, Department of Veterinary Medicine, Freie Universität Berlin (FUB), Berlin, Germany; ^4^ Animal Health-CReSA, Institute of Agrifood Research and Technology (IRTA), Bellaterra, Spain; ^5^ Chr. Hansen A/S, Animal Health Innovation, Hoersholm, Denmark; ^6^ Center for Evolutionary Hologenomics, The GLOBE Institute, University of Copenhagen, Copenhagen, Denmark; ^7^ University Museum, Norwegian University of Science and Technology (NTNU), Trondheim, Norway; ^8^ European Bioinformatics Institute (EMBL-EBI), Wellcome Genome Campus, Hinxton, United Kingdom

**Keywords:** animal performance, genomics, metagenomics, multi-omics, sustainability

## Abstract

Fast optimisation of farming practices is essential to meet environmental sustainability challenges. Hologenomics, the joint study of the genomic features of animals and the microbial communities associated with them, opens new avenues to obtain in-depth knowledge on how host-microbiota interactions affect animal performance and welfare, and in doing so, improve the quality and sustainability of animal production. Here, we introduce the animal trials conducted with broiler chickens in the H2020 project HoloFood, and our strategy to implement hologenomic analyses in light of the initial results, which despite yielding negligible effects of tested feed additives, provide relevant information to understand how host genomic features, microbiota development dynamics and host-microbiota interactions shape animal welfare and performance. We report the most relevant results, propose hypotheses to explain the observed patterns, and outline how these questions will be addressed through the generation and analysis of animal-microbiota multi-omic data during the HoloFood project.

## Introduction

With the ever-increasing human population on Earth, humanity is facing several major challenges to ensure long-term balance between natural resource use and environmental conservation (Reid et al., 2010). Many of the current agricultural practices are not sustainable due to excessive carbon emission, resource consumption, and waste production (Agovino et al., 2019). Hence, there is an urgent need to transition into a more resilient and sustainable agriculture model, in which the efficiency of production is improved, the use of antibiotics is reduced, and the welfare of animals is ensured (Eyhorn et al., 2019).

A fundamental step to optimise farming practices is to obtain in-depth understanding on the biological functioning of the animal production systems (Messerli et al., 2019). These systems often include biological elements beyond the actual animal that is being produced, among which host-associated microorganisms stand out due to their relevance for the optimal biological functioning of most animals (McFall-Ngai et al., 2013). Intestinal microorganisms not only modulate nutrient intake (Diaz Carrasco et al., 2019), but also shape intestinal immune and inflammatory processes (Zhou et al., 2020), intervene on host systemic growth parameters (Fraune and Bosch, 2010), and even influence host behaviour (Johnson and Foster, 2018). Microorganisms colonise the animal gut as soon as it is exposed to the environment (Sprockett et al., 2018), and develop communities with complex spatial and temporal dynamics (Debray et al., 2021), which continuously interact with the host animal (Khan et al., 2019; Ansari et al., 2020).

Animal-microbiota interactions have so far remained largely unexplored because of the limited capacity of scientists to properly characterise and analyse the key elements partaking in this interplay due to their excessive complexity (Alberdi et al., 2021). However, the development of high-throughput DNA sequencing and mass spectrometry technologies, linked to higher computing capacity and development of powerful bioinformatic tools, is changing this scenario (Graw et al., 2021). Today we are not only able to characterise the entire genetic information of animals and their associated microorganisms (namely the hologenome), but we can also quantify how genes are expressed, which proteins are synthesised and what metabolites result from enzymatic reactions happening in the gut (Nyholm et al., 2020). Such a holo-omic approach that considers multiple omic layers of both animals and associated microorganisms, is starting to unveil biological features and patterns that have remained hidden so far (Alberdi et al., 2021).

This technological revolution can contribute to many sources of variability that have been so far attributed to background noise, such as host microgenetic and microbiota variation among individuals, to be surfaced and included in the analyses (Alberdi et al., 2021). This requires an increased attention on the biological processes happening in each individual animal, rather than considering animals just as units that contribute to pen or tank statistic averages. The first attempts to implement individual-based multi-omic strategies in farm animals have provided detailed understanding of feed-microbiota-animal interactions (Andersen et al., 2021), by for example demonstrating which bacteria with which genes are able to degrade which carbohydrates (Michalak et al., 2020). We anticipate that such a mechanistic understanding of biological processes will contribute to generating refined hypotheses, predictive models and experimental treatments that will lead to a reduction of animals employed for research and an ultimate development of more optimal farming strategies.

HoloFood (HoloFood, 2019) is a multi-partner H2020 project that is pioneering such an approach, among other farming systems, on broiler chickens. The project aims at developing and implementing joint multi-omic analyses of animals and their associated microorganisms to generate in-depth knowledge of animal-microbiota interactions, and in doing so, improve the quantity, quality and safety of the produced food, the sustainability of the production process and the welfare of animals. To set the baseline to an upcoming series of publications that implement such multi-omic analyses, here we introduce the strategic vision, and experimental work conducted to generate biological samples and associated performance results of broiler chickens in the project HoloFood. We present the main performance results, discuss their relevance, and relate them to future multi-omic analyses that HoloFood partners will conduct to address the variety of biological questions raised from the initial screening of animal performance.

## Material and methods

### Animals and housing

The study consisted of three identical experiments (A, B, and C). In each trial a total of 960 days-of-hatch broiler chicks belonging to two fast growing genetic lines (Ross308^®^ and Cobb500^®^) from two hatcheries (to increase genetic variability) were allocated at 24 pens upon arrival. The name of genetic lines has been blinded and each one is described along the manuscript under the letter X or Y. In order to avoid the possible influence of the parent stock, birds were distributed in such a way that each replicate received the same number of broilers from each hatchery tray. Each pen had a total surface of 2.25 m^2^ with 40 birds per pen. Each pen was provided with one individual hopper feeder and two nipple drinkers. The barn is windowless and provided with automatic environment control with a gas heating system by screens and ventilation by depression. The room also has programmable lighting, provided by TL tubes evenly distributed. The temperature program was adjusted according to the standard program used in the farm: from 0 to 2 days the temperature increased from 32 to 34°C; from 3 to 7 days the temperature was reduced to 29–31°C and continued decreasing for 3°C per week afterwards until reaching 21°C. The lighting program was 24 h of light the first 2 days, 18 h of light until 7 days, and 14 h of light per day afterwards. The litter was fresh wood shavings. All birds were vaccinated against Avian Infectious Bronchitis and Gumboro diseases according to the vaccination program usually practised at the hatchery. Moreover, a set of 240 Cobb500 animals from one of the hatcheries in experiment 1 were vaccinated against Marek disease.

**FIGURE 1:**
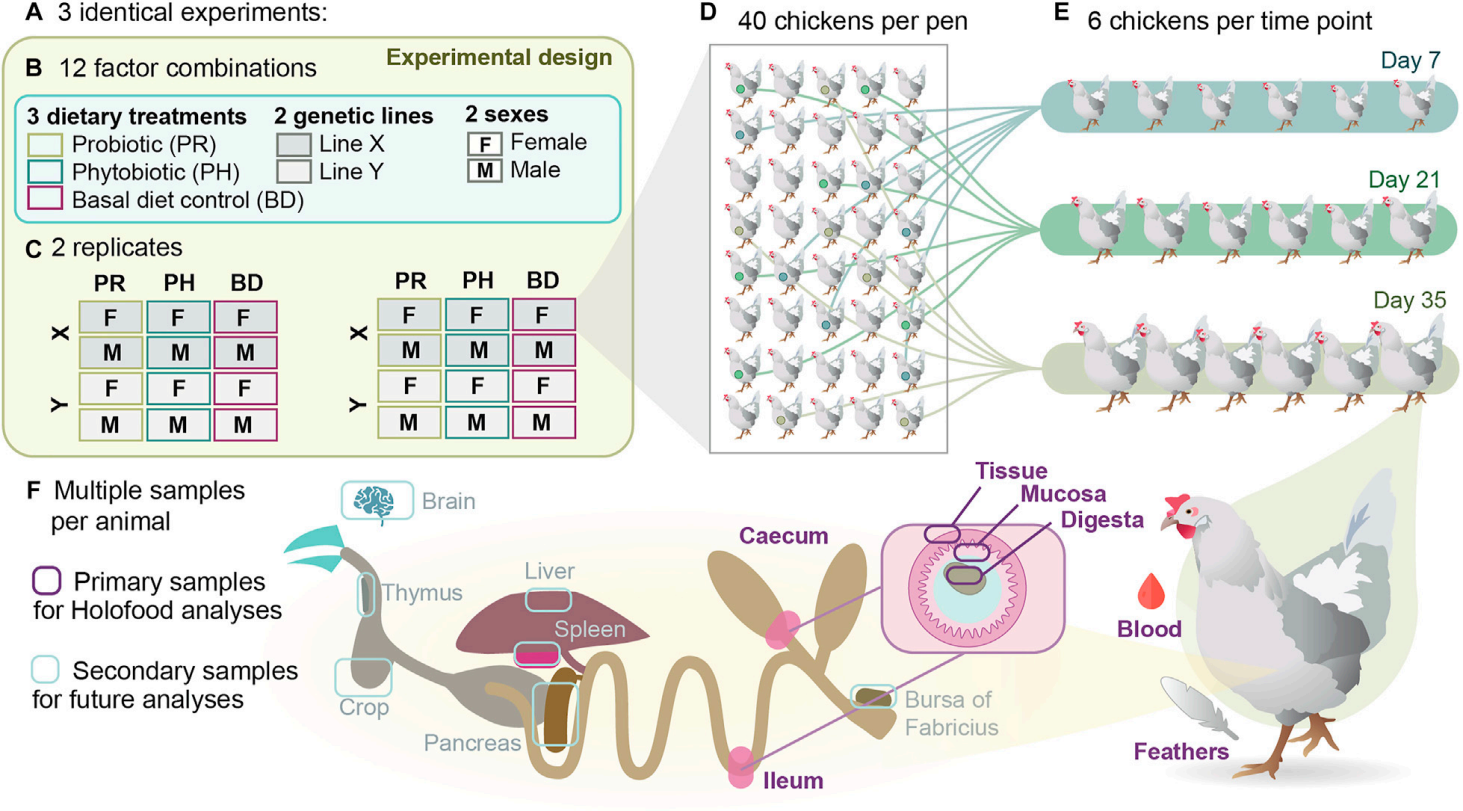
Experimental, sampling and analytical design of the HoloFood study focused on broiler chickens. **(A)** Three experiments with identical study design were conducted in 2019. **(B)** Each experiment contained 12 factor combinations of three dietary treatments, two genetic lines and two sexes. **(C)** Each combination was replicated twice per experiment, yielding 24 pens per experiment and 72 pens in total. **(D)** Each pen contained 40 chickens in the beginning of the experiment, 18 experimental animals that were randomly selected and tagged the first day of the experiment, and 22 more animals that provided commercial-like density conditions. **(E)** Six chickens were euthanised at day 7, six more at day 21 and six more at day 35 for collecting samples for performance, multi-omic and complementary analyses. **(F)** From each animal, 14 tissue and digesta samples were collected for a variety of analyses (see Table 1 for details), and complementary organ samples were also collected for future analyses.

The study had a randomized complete block design with a factorial 2 × 2 × 3 arrangement according to broiler line (X or Y), sex (male or female) and dietary treatment: 1) basal diet (BD), 2) BD plus a probiotic additive (PR), and 3) BD plus a phytobiotic additive (PH) (overview in Figure 1). The three trials were carried out in different seasons of the year: spring (trial A), summer (trial B) and autumn (trial C). Treatments were randomly assigned to one pen of each block (2 blocks per experiment), so that each treatment had 2 replicates (pens) per experiment (6 in total) with 40 animals per pen (20 animals from each hatchery). The experimental design deviates from conventional studies aimed at testing the effect of feed additives on performance, as it was designed seeking to maximise inter-individual genetic variability, and in doing so increase the probability to identify interactions between animal and microbial genomic features. The six animals to be slaughtered at days 7, 21, and 35 within each pen were randomly selected and marked at day 0 to avoid observer biases in subsequent samplings.

### Diets, additives and feeding

Aimed at maximising the effects of the feed additives, the BD was designed as a pro-inflammatory diet, using wheat (a cereal rich in non-starch polysaccharides (NSP) with a more than 50% inclusion) and soybean meal as main ingredients without the addition of enzymes, antibiotics, or coccidiostats. Diets were formulated according to birds’ requirements and commercial practices in three different periods: starter (0–9 days), grower (10–23 days) and finisher (24–37 days). Feeds were presented in crumble form for the starter period and in 3 mm pellets later on. Composition of diets and estimated nutrient contents are presented in Supplementary Table S1. The PR treatment consisted of BD with a mixture of three strains, namely *Bacillus subtilis* DSM 32324, *B. subtilis* DSM 32325 and *B. amyloliquefaciens* DSM 25840 (level of inclusion at 0.75 g/kg feed, >3.20 × 10^9^ CFU/g), which has been recently authorised as a zootechnical product for poultry species (EFSA Panel on Additives and Products or Substances used in Animal Feed et al., 2020). The PH treatment consisted of BD with a phytobiotic additive obtained from white grapes and containing 78% of procyanidins and 22% of polyphenols as active ingredients (level of inclusion at 0.75 g/kg feed).

Batch feed samples were taken from each production for proximate analysis (Association of Official Analytical Chemists, 2000, moisture -dry matter-by oven drying–method 2-, nitrogen -crude protein-by combustion -Dumas method-, ether extract on a Soxtec system -method 3B- and ash after muffle furnace incineration -method 12) and to quantify concentrations of probiotic and phytobiotic additives. Data of analytical composition of diets is shown in Supplementary Table S1. In addition, water and litter samples were collected in each pen at days 0, 7, 21, and 35 for microbiological and litter quality analyses. Moreover, three samples of each feed per diet period were taken from three different bags for microbiological assessment.

### Animal monitoring

Animals were counted and weighed by hatchery tray upon arrival and by replicate at days 7, 21, and 35. Growth performance was monitored to replicate the same days. Pen level (40 chickens/pen) analyses included average body weight (BW), daily gain (ADG), daily feed intake (ADFI), feed conversion ratio (FCR) and European production efficiency factor (EPEF). The incidence and severity of footpad dermatitis per pen was subjectively evaluated by trained personnel at days 7, 21 and 35 according the 5-point scale described by Butterworth (Butterworth and Welfare Quality Consortium and Normalisatie-instituut, 2009): 0) no evidence of footpad dermatitis; 1 and 2) minimal evidence of footpad dermatitis; 3 and 4) evidence of footpad dermatitis. Dead animals were weighted, and the most probable cause of death recorded. Animals (laggards) excluded from the trial during the first week were not considered.

### Animal sampling

At days 7–8, 21–22 and 35–37 (multiple days were necessary due to workload), 6 animals per pen were randomly selected, individually weighed (iBW), evaluated for footpad dermatitis (data not shown), euthanised and sampled. Animals were euthanised according to RD 53/2013 (Spain), following the ethical requirements established. After the euthanasia, a total of 14 samples were collected from each animal to measure individual key performance indicators (KPIs - iBW, concentration of corticosterone (COR) in feathers, concentration of acute phase proteins (C-reactive protein (CRP) and chicken haptoglobin-like protein (PIT54)), and lipopolisaccharides (LPS) concentration in plasma, and pathogens detection in caecal contents (Salmonella spp., Campylobacter spp., and Clostridium spp.)) as well as to generate multi-omic and complementary analyses (Table 1). Measured KPIs aimed at quantifying quantity, quality and safety of the produced food, the sustainability of the production process and the welfare of the produced animals. Sections of ileum and cecum, intestinal content from ileum and cecum, feathers, and blood were obtained. Different aliquots were distributed and properly stored for downstream analyses. Liver, pancreas, thymus, bursa of Fabricius, brain, and spleen samples were also obtained for future analyses (Figure 1F).

**TABLE 1:** Overview of the biological samples collected from each individual animal within the project HoloFood and their corresponding multi-omic and complementary analyses with data included in this article bolded.

Sample	Analyses
Blood	**Lipopolysaccharide and acute phase proteins**
Ileum tissue	Mucus production
	Histology
	Targeted amplification of inflammatory markers
	Shotgun chicken transcriptomics
Ileum mucosa	Shotgun chicken transcriptomics
	16S rRNA amplicon sequencing
Ileum content	Shotgun metagenomics
	16S rRNA amplicon sequencing
	Shotgun metatranscriptomics
	Chicken genomics
	Metabolomics
Caecum tissue	Mucus production
	Histology
	Targeted amplification of inflammatory markers
	Shotgun chicken transcriptomics
Caecum mucosa	Shotgun chicken transcriptomics
	16S rRNA amplicon sequencing
Caecum content	**Pathogen detection through PCR**
	Shotgun metagenomics
	16S rRNA amplicon sequencing
	Shotgun metatranscriptomics
	Chicken genomics
	Metabolomics
Feathers	**Corticosterone measurement**

In addition, at day 37 (commercial slaughtering age), 6 extra animals per pen were randomly selected, euthanised and subjected to evaluation of meat quality traits (carcass, abdominal fat, breast, and leg yield). The oxidative stability of the thigh muscle was determined over a period of 7 days from randomly selected three animals used for meat quality assessment.

The data on each individual animal derived from the experiments were analysed at three different levels. The first level included the analyses of measurements obtained in the farm (e.g., iBW). The second level comprised analyses conducted *a posteriori* from samples obtained during the trials in order to control the health, welfare of animals (e.g., ELISA for CRP, PIT54, LPS and COR quantification, and PCR for pathogen detection), and meat quality traits (e.g., meat oxidative stability). The third level included multi-omic analyses, which characterises the animal-microbiota system at the highest level of breadth and resolution. These analyses will include whole-genome sequencing of chicken genomes, metagenomics of microbial communities (meta)transcriptomics and metabolomics. This third level of data will be carried out in the next steps of the project.

### Analytical procedures

#### Pathogen detection

Opportunistic infections of different zoonotic pathogens (*Salmonella, Campylobacter and Clostridium* spp.) were controlled in caecal contents from sampled animals at days 7, 21 and 35 by conventional PCR (absence*/presence*)*. Salmonella* spp. was detected using the primers 5′-GTG​AAA​TTA​TCG​CCA​CGT​TCG​GGC​AA-3′ and 5′-TCA​TCG​CAC​CGT​CAA​AGG​AAC​C-3′, which are specific for the InvA gene of *Salmonella*, following the conditions described in Rahn et al. (1992). For the detection of *Clostridium perfringens*, the primers 5′-AAG​ATT​TGT​AAG​GCG​CTT-3′ and 5′-ATT​TCC​TGA​AAT​CCA​CTC-3′ specific for alpha-toxin gene present in all strains of this bacteria species were used. The specific amplification program was as follows: 94°C/4’; (94°C/1′, 55°C/1′, 72°C/1′20″)x35; 72°C/15’; 4°C/end. The presence of *Campylobacter* spp. was detected using the primers 5′-TTG​GAA​ACG​ACT​GCT​AAT​ACT​CTA-3′ and 5′-AGC​CAT​TAG​ATT​TCA​CAA​GAG​ACT-3′, which amplify a specific segment of 16S ribosomal RNA gene specific of *Campylobacter* spp. The specific amplification program was as follows: 94°C/4’ (94°C/1′, 48°C/2′, 72°C/1′)x35; 72°C/15’; 4°C/end.

#### Corticosterone in feathers

Corticosterone was determined in accordance with the method described by Bortolotti et al. (2008).

#### Acute phase proteins in plasma

Chicken haptoglobin-like protein (PIT54) was quantified using the haptoglobin ELISA Kit (Ref. ABIN1563052, antibodies-online.com) and C-reactive protein (CRP) was measured using the CRP ELISA Kit (Ref. ABIN4947413, antibodies-online.com) in accordance with manufacturer instructions.

#### Lipopolysaccharide in plasma

Lipopolysaccharide concentration in plasma was determined using Pierce LAL Chromogenic Endotoxin Quantification Kit (Ref. 88282; Thermofisher, United States) in accordance with manufacturer instructions.

#### pH of litter

Three samples of 50 g of litter per pen were collected for pH determination at days 7, 21 and 35 avoiding the areas near and below the feeders and drinkers. The three samples collected from the same pen and day were pooled and homogenised and the moisture was determined in a sub-sample of 100 g according to the AOAC method Association of Official Analytical Chemists, 2000 method 925.09). For pH analysis, a subsample of 10 g was placed in a beaker with 100 ml of distilled water, shaked with a glass rod and allowed to stand for 30 min. The pH value was obtained using a pH metre (Crison, L’Hospitalet de Llobregat, Spain).

#### Oxidative stability of meat

Samples (5 g of muscle from the thigh) were homogenised with an aqueous 7.5% trichloroacetic acid solution, filtered and brought to 20 ml. To proceed, 5 ml of the extraction solution and 5 ml of 0.02 M thiobarbituric acid were mixed and boiled for 15 min and then cooled in cold water. Absorbance of the peak was measured at 525 nm as malondialdehyde production in an ultraviolet-visible spectrophotometer (Shimadzu, Japan) using the third derivative of the spectrum between 425 and 650 to correct the baseline. The 1,1,3,3-tetraethoxypropane was used as standard (Botsoglou et al., 1994; Ruiz et al., 1999).

### Statistical analyses

Data was explored to discard any possible outlier according to the Kolmogorov-Smirnov test (Massey, 1951). As no outliers were considered, the statistical analysis included all data. The GLIMMIX procedure of SAS software (SAS/STAT 14.1; SAS Institute Inc., Cary, NC, United States) was used to perform the analysis of the different variables. In the case of ELISA determinations, when the limit of detection was not reached, the missing values were replaced by the limit of detection (L)/√2 (Hornung and Reed, 1990). The statistical model used is shown below:

$${\text{y}}_{\text{ijklm}}=\mu +{\text{T}}_{\text{i}}+{\text{B}}_{\text{k}}{+\text{S}}_{1}+{\text{E}}_{\text{m}}+\gamma_{\text{j}}{+\text{e}}_{\text{ijklm}}$$

Where y_ijklm_ is the response variable, T_i_ is the dietary treatment effect, B_k_ is the broiler line effect, S_l_ is the biological sex effect, E_m_ is the experiment effect, γ_j_ is the random block effect, and e_ij_ is the error of the experimental unit. The experimental unit for the statistics and the tables presented was the pen. Results in Supplementary Tables S2–S12 are expressed as least square means ± standard error. Differences were considered significant at *p* < 0.05, while those at *p* < 0.1 are reported as tendencies.

Hierarchical decomposition of the variance was carried out using ANOVA-estimation of variance components as implemented in the fitVCA function of the R package VCA (Schuetzenmeister and Dufey, 2017), using iBW as response variable, and pen and experiment as explanatory variables.

## Results

In the following, we present the main results obtained from pen-level analyses, and the first two levels of the individual analyses sorted by topic. We explain their biological relevance based on current knowledge, and we project the potential of multi-omic analyses that will be conducted in the project HoloFood on top of these results to address the most relevant pending questions.

### Effect of dietary treatments

**FIGURE 2:**
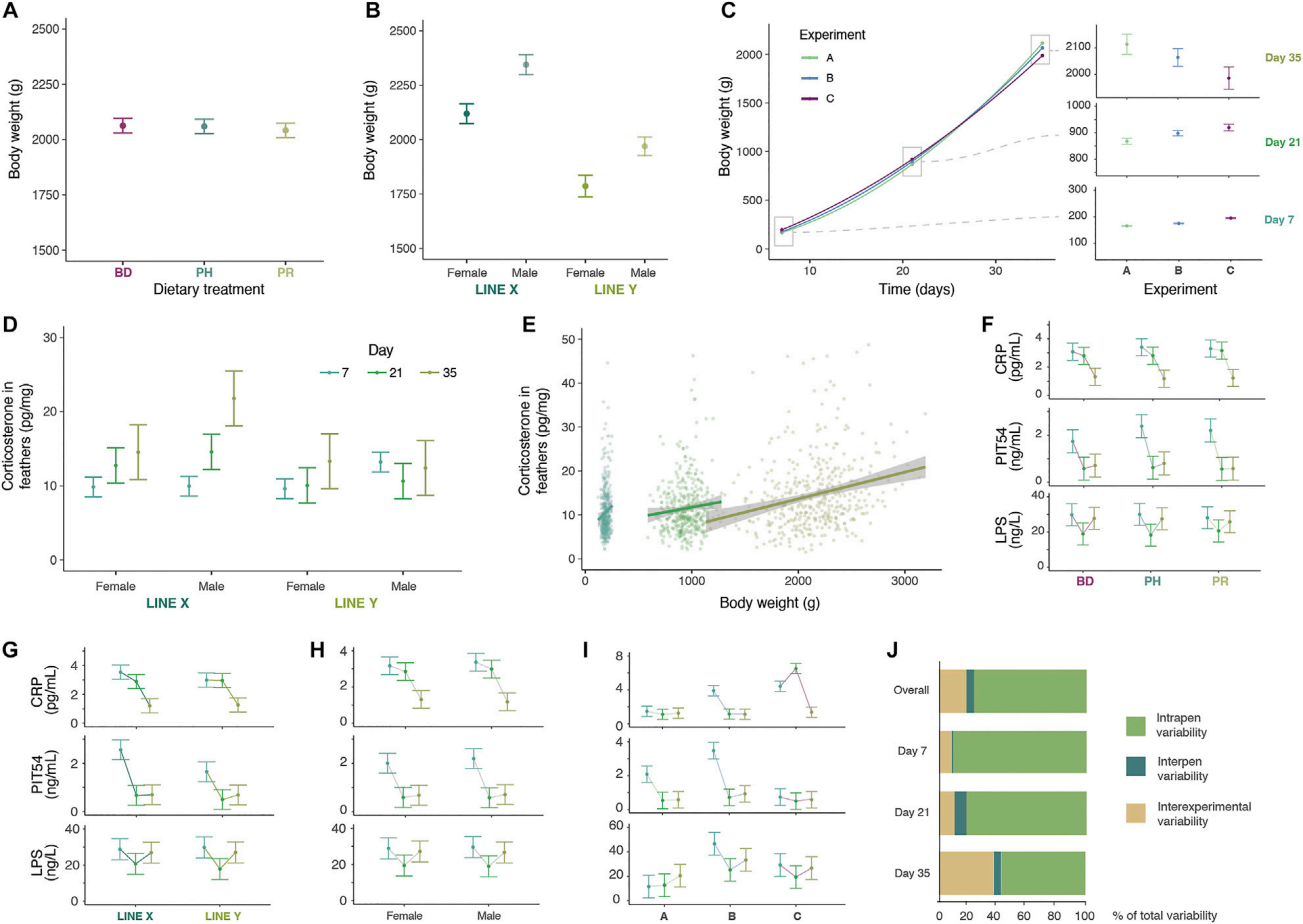
Overview of main results. **(A)** Body weight differences across dietary treatments, namely basal diet (BD), BD plus probiotic (PR) and BD plus phytobiotic (PH), at day 35. **(B)** Body weight differences across lines and sexes at day 35. **(C)** Body weight progression of the three experiments, with detailed overview of days 7, 21 and 35. **(D)** Corticosterone (COR) levels measured in feathers at different days, sexes, and genetic lines. **(E)** Linear correlation between COR levels and body weight at the three time points. **(F)** C-reactive protein (CRP), avian haptoglobin-like protein (PIT54) and lipopolysaccharide (LPS) levels in plasma across time points in different dietary treatments. **(G)** CRP, PIT54 and LPS levels in plasma across time points in different genetic lines. **(H)** CRP, PIT54 and LPS levels in plasma across time points in different sexes. **(I)** CRP, PIT54 and LPS levels in plasma across time points in different experiments. **(J)** Hierarchical decomposition of observed body weight variation within pens, among pens and among experiments.

No significant effect of administered dietary additives was observed in the performance of the animals (Figure 2A; Supplementary Tables S2-S12). Dietary treatments neither induced any significant change in the acute phase protein values measured in plasma (Figure 2F, Supplementary Table S7). The only parameter affected by dietary treatments was COR (Supplementary Table S6), which was increased with the PH diet at day 7, but these levels were not prolonged over time nor decreased compared to the BD.

### Effect of broiler line

Broiler line X reached significantly higher BW, ADG, and ADFI and lower FCR than line Y at day 35 (2232 g and 1878 g for BW (standard error (SE) = 18.8), 62.6 and 52.4 g for ADG (SE = 0.54), 96.6 and 81.1 g for ADFI (SE = 0.80), and 1.497 and 1.542 for FCR (SE = 0.015), respectively; Figure 2B and Supplementary Table S2).

The accumulation of COR in feathers was significantly higher for line X (13.91 pg/mg) than for line Y (11.54 pg/mg) (SE = 0.914; *p* < 0.001) (Figure 2D, Supplementary Table S6) and individual COR levels were also higher in animals with higher iBW (Figure 2E). PIT54 levels in plasma were also higher in line X at days 7 (*p* = 0.0687) and 21 (*p* = 0.001) than in line Y (+35.5% and +25.4%, respectively) (Figure 2G, Supplementary Table S7B) and for the whole experiment (*p* = 0.0354; +27.5%).

### Effect of biological sex

Male chickens exhibited larger average BW than females at all time points, reaching an 8.7% larger BW at day 35 (2157 and 1953 g, respectively (SE = 13.8); Figure 2B). The rest of performance traits were also improved in males compared to females (ADG, ADFI, FCR, and EPEF) (Supplementary Table S2).

Males showed higher COR levels than females in the period 0–35 days (13.8 vs. 11.7 pg/mg; *p* < 0.001; SE = 0.91) and also at day 7 and at day 35 (*p* < 0.001 and *p* = 0.028, respectively) (Figure 2D, Supplementary Table S6). In the last period of the experiment (at day 35), the levels of CRP in plasma were higher in females than males (1.31 vs. 1.18 ng/mg; SE = 0.08; *p* = 0.045) (Figure 2H, Supplementary Table S7B).

### Effect of age and development

Corticosterone accumulation in feathers increased as animals grew (Supplementary Table S6; Figure 2E), while the levels of PIT54 and CRP in plasma peaked at day 7, and decreased through time (Supplementary Table S7B; Figures 2F,G).

### Effect of zoonotic pathogens

Targeted detection of three common zoonotic pathogens, namely *Salmonella*, *Clostridium* and *Campylobacter* spp. was performed to detect whether natural colonisation of the chicken intestines occurred during the trials. Only one animal in trial A and another in trial B were positive for Clostridium, being all the animals negative for Salmonella and Campylobacter. However, in trial C, the analyses revealed that 13.7% of the sampled animals at day 7 were positive for *Salmonella*, but the colonisation vanished as the animals grew. Moreover, 99% of the birds sampled from day 21 onwards presented a *Campylobacter* colonisation, with no prevalence differences between dietary treatments. The detection of *Campylobacter* was correlated with a drop in BW from day 21 onwards compared to the two previous trials (Figure 2C). In addition, a peak of CRP values in plasma was detected in *Campylobacter* positive animals at day 21 (Figure 2I).

### Hierarchical variance decomposition of chicken body weight

Due to our interest in generating systemic characterisation of individual chickens, we explored how the variance of iBW data for each combination of factors (i.e., treatment, biological sex and genetic line) was distributed across the three hierarchical levels of the study: 1) variation across the six animals sampled in each pen at each time point, 2) variation between the two pen replicates within each experiment, and 3) variation among the three experiments.

The average coefficient of variation for iBW of the six sampled animals within each pen was 11.08%, with maximum values reaching 22.5% (Supplementary Table S12). At day 35, the average BW difference between the largest and smallest animal sampled in each pen was over 31% of the mean value. The intrapen variability explained most (76.3%) of the variance observed within combinations of factors (Figure 2J). However, its weight with respect to experiment factor decreased with the age of the animal.

## Discussion

### Effect of dietary treatments

*Bacillus* spp. are commonly used as additives in broiler production (Irta, 2015), and the specific probiotic strains tested in our study have been previously shown to improve performance and physiological traits in broilers (Molnár et al., 2011; Goodarzi Boroojeni et al., 2018). However, contrasting observations that align with our results have also been reported (Li et al., 2018), which could be explained by varying experimental conditions, such as the specific *Bacillus* strain employed, the dose, or the basal diet. The supplementation of poultry diets with multi-strain *Bacillus* probiotic products is in general associated with competitive exclusion of common pathogens, improved nutrient digestion and absorption through the production of exogenous enzymes, enhanced intestinal morphology, and the modulation of relevant immune system pathways (Ramlucken et al., 2020; Tarradas et al., 2020). Regarding the phytobiotic, multiple modes of action have been attributed to additives containing polyphenols and procyanidins, including antioxidant and anti-inflammatory properties, promotion of beneficial bacteria in the gut, and enhancement of nutrient absorption through binding of dietary proteins and carbohydrates (Chamorro et al., 2019; Hasted et al., 2021).

As the beneficial effect of feed additives is usually not evident when animals grow under optimal conditions (Vilà et al., 2010), we deliberately induced a challenging condition through increasing the amount of dietary soluble NSP. These compounds are known to have deleterious effects on the bird’s health and performance through increasing intestinal viscosity and hampering nutrient digestibility (Raza et al., 2019), and can thus maximise the beneficial effects of feed additives (Bortoluzzi et al., 2019; Whelan et al., 2019). Accordingly, the overall performance was 13.8% lower than the expected from reference performance tables (Cobb-Vantress, 2018; Aviagen, 2019), yet with no differences between treatments.

COR measured in feathers is used as a biomarker of accumulative stress (Bortolotti et al., 2008), as chronic levels of COR are associated with detrimental effects on growth and related biological traits (Scanes, 2016). The increase of COR with the PH diet at day 7, and the disappearance of this effect throughout the study contrasts with previous studies that reported a reduction of serum and feather COR in broilers fed with polyphenol extracts (Gong et al., 2018; Gopi et al., 2020).

To delve into the reasons under, among other questions, the lack of positive effect of the feed additives on commonly assessed nutritional parameters, HoloFood will generate deep genome-resolved metagenomic datasets (Almeida et al., 2019; Pasolli et al., 2019) from the ileal and caecal content. While most chicken-associated microbiota research is being conducted using targeted sequencing (Mohd Shaufi et al., 2015; Jurburg et al., 2019; Ocejo et al., 2019), the first shotgun-sequencing based studies have recently been published (Glendinning et al., 2020; Gilroy et al., 2021). The metagenome-assembled genome (MAG) catalogue of bacteria associated with broiler chickens generated in HoloFood, will not only complement such efforts for the high-resolution characterisation of chicken-associated microbiomes, but will also enable in-depth study of strain-level microbiota dynamics in the analysed production context through combining taxonomic and direct functional inferences. The reconstructed bacterial genomes will be functionally annotated, thus directly inferring the metabolic capacities (e.g., complex polysaccharide degradation, short-chain fatty acid (SCFA) production) of strains, and acknowledging the aggregated functional landscape of the entire community present in each animal (Shaffer et al., 2020). HoloFood will also generate whole genome sequences of the probiotic strains through hybrid short- and long-read DNA sequencing to ensure highest-quality genome reconstructions of the tested probiotics (Wick et al., 2017), and perform a pangenome analysis with other sequenced and annotated Bacillus strains to identify bacterial genes that could confer beneficial functional capabilities each of the strains. This will enable us to understand the specific means of action through which each strain can interact with the microbiota and various intestinal features of the host. All these analyses will enable ascertaining the relative abundances of *Bacillus* probiotics in different intestinal segments, and measuring whether the additives trigger broad-as well as fine-scale taxonomic and/or functional changes in the microbiota of broilers, that could contribute to explain the observed results.

### Effect of broiler line

Although theoretically both lines tend to perform similarly in terms of growth and final BW (Cobb-Vantress, 2018; Aviagen, 2019; Livingston et al., 2020), line X reached significantly higher BW, ADG, and ADFI and lower FCR than line Y at day 35. The differences observed on performance between lines could partially be explained by the higher initial BW of line X (44.5 g) at day 0 than line Y (40.8 g) (SE = 0.31; Supplementary Table S2). A retrospective analysis of the breeders’ features showed that the age of breeders was higher for line X (49.6 ± 10.1 weeks) than for line Y (44.1 ± 11.4 weeks), which probably caused the observed difference between the initial BW (Iqbal et al., 2017). However, the differences between lines at day 35 did not disappear even when BW at day 0 was included as a covariate in the statistical model, suggesting that other factors contributed to shape the differences observed between both lines. Therefore, performance results suggest that line X could exhibit a higher resistance to NSP or a more active feeding behaviour than line Y.

The higher accumulation of COR in feathers in the broiler line X or in the animals with a higher iBW could be explained by an increased growth rate promoting deposition of COR in feathers (Jimeno et al., 2018), in contrast to what would be expected from a stress indicator (Carbajal et al., 2014). On the other hand, PIT54 is an acute phase protein with an important inhibitory role in inflammation processes (Wicher and Fries, 2006), which is rapidly increased in the blood as a response to infectious agents or physiological stressors (O’reilly and Eckersall, 2014). PIT54 in the chicken plasma binds free haemoglobin to inhibit haemoglobin-mediated oxidation of lipid and protein (Ahn et al., 2019). Antinutritional effects of NSP are related to a reduction of BW and FCR, and can trigger a mild chronic inflammation in the gut (Cardoso Dal Pont et al., 2020). Host gut inflammatory response produces reactive oxygen species (ROS), which cause oxidative stress and have the potential to damage host tissue (Costantini and Møller, 2009). The increased antioxidant activity through the high levels of PIT54 in line X could explain the better performance compared with line Y through the amelioration of antinutritional effects of NSP.

The observed differences between broiler lines point to differences in systemic responses to the pro-inflammatory diet, which probably yielded the BW and associated KPI differences between the two genetic lines. The whole-genome analyses we will conduct in HoloFood will enable deepening into these observations through unveiling genome-wide differences between both lines. We will perform whole genome resequencing to generate single nucleotide polymorphism (SNP) profiles of all individuals (Li et al., 2009). This will enable testing whether both lines have genetic differences in key genes related to inflammatory responses induced by a high dietary concentration of NSP, as well as key metabolic pathways such as steroid hormone biosynthesis (Kanehisa et al., 2021). In addition, we will also generate *de-novo* reference genomes (Baker, 2012) and Hi-C maps (Lieberman-Aiden et al., 2009) of both lines, to explore the effects of structural genome variants (e.g., copy number variations (CNVs), translocations, inversions) in the performance differences observed. Such chicken genomic data will be coupled with the aforementioned microbial metagenomic information, which will allow exploring whether and how host-microbiota interactions orchestrate different physiological responses to nutritional stress in the two broiler lines, as previously reported in other taxa (Ma et al., 2019).

### Effect of biological sex

The differences between biological sex among chickens observed in the current experiment are expected (Cobb-Vantress, 2018; Aviagen, 2019), as growth patterns of male chickens outperform that of females (Aggrey, 2002; Hausman et al., 2014; Livingston et al., 2020). However, unlike in previous studies (Carbajal et al., 2014), males showed higher COR levels than females, supporting again that COR cannot directly be negatively associated with welfare and performance of animals (Jimeno et al., 2018). On the other hand, females showed higher levels of CRP than in males at day 35. CRP is an acute phase protein used as a highly sensitive marker of inflammation and tissue damage (O’reilly and Eckersall, 2014). Some immune biomarkers including CRP are influenced by biological sex in other production animals such as pigs (Gutiérrez et al., 2018). However, the reasons behind biological sex differences for inflammatory markers are still unknown.

In HoloFood we will aim at further understanding these differences in growth between both sexes, as well as other questions mentioned above, by generating whole-genome transcriptomic data (RNAseq) to identify gene expression differences in the intestinal tissues. While intestinal expression of targeted genes is routinely measured in animal sciences (Slawinska et al., 2019; Farahat et al., 2021), how genome-wide gene expression varies across sexes and intestinal sections is still largely unknown. As intestinal gene expression is known to be modulated by the microbiota (Volf et al., 2017), and biological sex also contributes to shaping microbial communities (Lee et al., 2017), we will aim at detecting associations between host expression patterns and microbial communities to ascertain host-microbiota interactions related to sex differences. For instance, we will analyse expression of host genes involved in cholesterol (precursor of steroids, and thus related to COR) absorption, such as NPC1L1 and ABCG5/ABCG8, which have been shown to be modulated by the microbiota in rodents (Zhong et al., 2015). Sample collection in HoloFood was extended to organs beyond the gastrointestinal tract, including liver and brain, which are involved in appetite regulation and other processes related to the gut-brain axis (Cryan et al., 2019). The analysis of gene expression in such organs along with gut processes, will enable delving into the relationship between host genetics and microbiota factors with nutrient metabolism in shaping feeding behaviour and related performance differences in broiler chickens.

### Effect of age and development

Animal development is linked to multiple changes in analysed metrics. For instance, COR in feathers increased as animals grew, mirroring previous observations (Nordquist et al., 2020). The pattern observed by the levels of PIT54 and CRP in plasma (peaked at day 7 and decreased through time), exhibit a trend that could be linked to vaccination as well as to microbiota development. On the one hand, vaccines are known to increase concentration of acute phase proteins and stress markers during the first days after administration (Kaab et al., 2018), and all chickens in the experiment were vaccinated against Avian Infectious Bronchitis and Gumboro diseases at hatch. On the other hand, early microbial colonisation of the intestine is also known to boost the development of the immune system (Broom and Kogut, 2018) through, for example, the production of the intestinal mucus layer (Duangnumsawang et al., 2021), which provides the first protective shield preventing a direct access of pathogenic bacteria to the epithelial surface.

HoloFood will also generate and analyse microbiota-wide gene expression in the chicken intestine, as well as metabolites that play essential roles in the host-microbiota interplay, such as SCFAs (van der Hee and Wells, 2021). Our study design, which entails euthanising animals at each sampling point, prioritises spatial resolution of intestinal sections over temporal development, which complicates tracking the temporal development of individual animals. However, sampling at three different time points will provide an overview of how much microbiota development varies across individuals (Sprockett et al., 2018; Debray et al., 2021; Ballou et al., 2016; Jurburg et al., 2019). Shotgun metatranscriptomic data will complement the metagenomic information, thus providing not only an overview of the relative abundances of different bacterial taxa in different time points, but also displaying the gene expression patterns of the bacteria. In addition, metabolomic data will enable validating whether the activated metabolic pathways are translated into different levels of SCFA concentrations. We will measure how the expression of microbial genes involved in carbohydrate metabolism and SCFA production vary across development, and how these changes are associated with animal growth and changes observed in acute phase protein levels.

### Effect of zoonotic pathogens

*Campylobacter* and *Salmonella* are usually considered mere commensals in poultry, but they cause the highest numbers of foodborne diseases in humans globally (European Food Safety Authorityand European Centre for Disease Prevention and Control, 2021). Recent studies have nevertheless shown that *Campylobacter* colonisation in chickens can cause gut microbiota alterations and intestinal damage that occasionally facilitates bacterial colonisation of extraintestinal organs, which may eventually lead to a reduced animal performance and welfare (Awad et al., 2015, 2018). In accordance with these observations, the detection of *Campylobacter* was correlated with a drop in BW from day 21 onwards. In addition, the peak of CRP detected in *Campylobacter* positive animals at day 21, would most probably indicate systemic reaction to the colonisation (Liu et al., 2019; Zhang et al., 2020). Other opportunistic pathogens from the *Campylobacterales* order, such as *Helicobacter brantae,* which may be present along with *Campylobacter* but undetected with targeted approaches, might likewise be involved in performance drop (Kollarcikova et al., 2019).

The bacterial genome catalogue we will build in HoloFood will not only enable us to ascertain whether the natural *Campylobacter* colonisation was due to a single or multiple strains (Chaloner et al., 2014), but also to characterise the entire catalogues of genes of these strains and thus identify potential virulence factors that seem to be inducing an inflammatory response. We will be able to measure whether the *Campylobacter* colonisation correlated with any systemic change in the microbiota and in the intestinal response of the animals, through combining gene expression data of chickens and microorganisms as well as metabolomic information. *Campylobacter* induces the expression of various host pro-inflammatory cytokines through the activation of Toll-like receptor 4 (TLR4) and TLR21 signalling pathways (de Zoete et al., 2010). Our analyses will enable measuring changes in the expression levels of genes involved in these signalling pathways between Campylobacter positive and negative animals, to deepen into the effect of *Campylobacter* in chicken welfare and performance.

### Hierarchical variance decomposition of chicken body weight

In the current study, the mentioned individual variability was higher than previously reported (Vasdal et al., 2019; Lundberg et al., 2021), although chicken BW variation can increase if animals are subjected to challenging diets (Gous, 2018). The reduction of the intrapen variability with the age of animals, would probably be due to augmenting environmental effects and potentially microbiota development factors. Although the three experiment replicates were identical, and abiotic conditions were controlled in the farm, these were conducted in spring, summer, and autumn, which could have entailed slight differences in the temperature and humidity of the barn.

The whole-genome analyses we will perform in HoloFood will enable ascertaining whether the interindividual genetic variability could be related to the observed dispersion of the data. Genotype-phenotype association studies in commercial chicken lines have identified several important genomic regions that explain a percentage of the BW variation (Tarsani et al., 2019; Wang et al., 2020; Dadousis et al., 2021). In addition, the variability in the intestinal microbiota can intensify differences in performance between individuals from the same population (Yan et al., 2017; Shah et al., 2019; Wen et al., 2021). Ultimately, we will aim at identifying chicken genetic variants associated with microbiota changes with noteworthy impact on performance. These chicken genomic and microbial metagenomic analyses will enable ascertaining to which degree the observed intrapen and interexperiment variation can be attributed to differences in the genetic features of chickens and microbial communities.

## Conclusion

**FIGURE 3:**
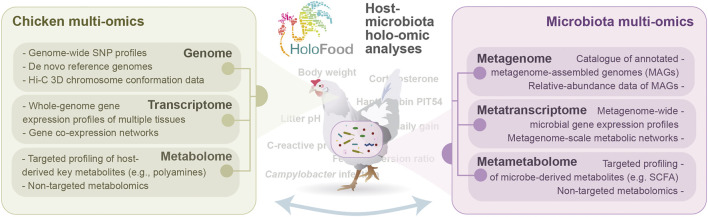
Overview of holo-omic analyses that will be conducted in the H2020 project HoloFood to deepen into the results outlined in this manuscript and address the questions raised and beyond.

To conclude, the range of analytical approaches outlined in this article will give us the opportunity to showcase the strength of implementing new multi-omic approaches to address relevant questions for farming practices. Many questions that would remain unanswered by employing traditional techniques can now be addressed using these new technologies, and most importantly, new questions that were not so far set out (e.g., the reasons for intra-pen variability) can be now proposed. We believe that the hologenomic approach being implemented in HoloFood will help us move from “Does factor X affect KPI Y?” to “How and why does factor X affect KPI Y?”. That is to say, transitioning from a trial-and-error approach to a knowledge-based strategy in which understanding biological processes that underlie the administration of feeds, additives or drugs, as well as the observed interindividual variation, is prioritised. We will address all the questions outlined in this article and more through the collaboration of multiple academic and industrial partners, aimed at pioneering the large-scale implementation of hologenomics in animal farming (Figure 3). Although HoloFood will generate and analyse one of the largest multi-omic datasets in farm animals with characterisation of hundreds of specimens, we acknowledge the mathematical challenges of analysing such a complex and hyper-dimensional dataset. The dimensionality of the data will be reduced by leveraging the hierarchical structure of biology itself (e.g., enzymes embedded within metabolic pathways), as well as using the most advanced feature selection approaches to identify the most relevant molecular elements. Ongoing data analyses, which will be published in upcoming articles, will show us how far we can reach, what are the limitations of this novel approach, and how the field can best advance to make the most of the new technologies for a more secure, ethical and sustainable food production.

## Data Availability

The original contributions presented in the study are included in the article/Supplementary Material, further inquiries can be directed to the corresponding author.
