# Supplementary material for: Novel strategies to improve chicken performance and welfare by unveiling host-microbiota interactions through hologenomics
Source: Front Physiol. 2022 Sep 6;13:884925. doi: 10.3389/fphys.2022.884925 (PMC9485813; doi:10.3389/fphys.2022.884925)
Supplement: Supplementary file 1 [file Table1.DOCX]

Supplementary Material

**Supplementary Table S1:** Ingredient and nutrient composition of the basal diets (as fed basis).

|  | **1^st^ Experiment (A)** | | | **2^nd^ Experiment (B)** | | | **3^rd^ Experiment (C)** | | |
| --- | --- | --- | --- | --- | --- | --- | --- | --- | --- |
| **Period (days)** | **0-7** | **7-21** | **21-35** | **0-7** | **7-21** | **21-35** | **0-7** | **7-21** | **21-35** |
| **Ingredient (g/kg)** |  |  |  |  |  |  |  |  |  |
| Wheat | 52.75 | 61.24 | 61.97 | 52.31 | 60.52 | 61.05 | 54.3 | 62.19 | 62.15 |
| Soybean 48% | 39.4 | 30.5 | 15.8 | 39.88 | 31.24 | 16.79 | 38.02 | 29.66 | 15.75 |
| Extruded Soyabean | - | - | 15 | - | - | 15 | - | - | 15 |
| Soyabean oil | 4.16 | 4.8 | - | 4.18 | 4.85 | - | 4.01 | 4.71 | - |
| Animal fat (5 Sysfeed)^1^ | - | - | 4.01 | - | - | 4.08 | - | - | 3.98 |
| Dicalcium phosphate | 1.84 | 1.66 | 1.5 | 1.84 | 1.66 | 1.5 | 1.86 | 1.67 | 1.5 |
| Calcium carbonate | 0.53 | 0.48 | 0.44 | 0.48 | 0.44 | 0.39 | 0.49 | 0.44 | 0.39 |
| Vitamin-Mineral premix^2^ | 0.4 | 0.4 | 0.4 | 0.4 | 0.4 | 0.4 | 0.4 | 0.4 | 0.4 |
| Sodium chloride | 0.37 | 0.37 | 0.53 | 0.37 | 0.38 | 0.36 | 0.37 | 0.35 | 0.35 |
| DL-methionine | 0.27 | 0.23 | 0.19 | 0.28 | 0.24 | 0.2 | 0.28 | 0.23 | 0.19 |
| L-lysine HCL | 0.16 | 0.19 | 0.15 | 0.15 | 0.17 | 0.14 | 0.16 | 0.18 | 0.15 |
| Choline Chloride | 0.03 | 0.05 | 0.05 | 0.03 | 0.05 | 0.05 | 0.05 | 0.06 | 0.06 |
| L-threonine | 0.05 | 0.05 | 0.04 | 0.05 | 0.05 | 0.04 | 0.05 | 0.05 | 0.04 |
| Sodium bicarbonate | - | 0.01 | - | - | - | - | - | 0.04 | - |
| Antioxidant (Noxyfeed 56P)^3^ | 0.02 | 0.02 | 0.02 | 0.02 | 0.02 | 0.02 | 0.02 | 0.02 | 0.02 |
| **Nutrients (g/kg)** |  |  |  |  |  |  |  |  |  |
| Metabolizable Energy (Kcal/kg) | 2900 | 3000 | 3100 | 2900 | 3000 | 3100 | 2900 | 3000 | 3100 |
| Dry Matter | 879 | 881 | 882 | 879 | 881 | 882 | 871 | 874 | 878 |
| Crude Protein | 255 | 223 | 206 | 241 | 209 | 194 | 241 | 210 | 195 |
| Ether Extract | 57.0 | 63.4 | 81.5 | 57.0 | 64.0 | 82.0 | 56.8 | 63.8 | 82.6 |

^1^Product of Sysfeed SLU (Granollers, Spain). It contains myristic acid (C14:0) 1.50%, palmitic acid (C16:0) 18.0%, palmitoleic acid (C16:1 n-7) 2.00%, stearic acid (C18:0) 14.0%, oleic acid (C18:1 n-9 cis) 28.0%, linoleic acid (C18:2 n-6 cis) 12.0%, α-linolenic acid (C18:3 n-3 cis) 6.00%, saturated–unsaturated 0.7%.

^2^Vitamin-Mineral premix: Product of TecnoVit S.L. (Alforja, Spain). Supplied per kilogram of feed: Vitamin A: 10 000 IU; Vitamin D3: 4 800 IU; Vitamin E: 45 mg; Vitamin K3: 3 mg; Vitamin B1: 3 mg; Vitamin B_2_: 9 mg; Vitamin B_6_: 4.5 mg: Vitamin B_12_: 40 ug; Folic acid: 1.8 mg; Biotin: 150 ug; Calcium pantothenate: 16.5 mg; Niacin: 65 mg; Mn (as MnSO_4_.H_2_O): 90 mg; Zn (as ZnO): 66 mg; I (as KI): 1.2 mg; Fe (as FeSO_4_.H_2_O): 54 mg; Cu (as CuSO_4_.5H_2_0): 12 mg; Se (as NaSeO_3_): 0.18 mg; BHT: 25 mg; Calcium formiate, 5 mg; Silicic acid, dry and precipitated, 25 mg; Calcium stearate , 25 mg; Calcium carbonate to 4 g.

^3^Product of Itpsa (Barcelona, Spain). It contains 56% of antioxidant substances (butylated hydroxytoluene + propyl gallate) and synergistic (Citric acid 14% + authorised support).

**Supplementary Table S2:** Performance 0-35 days^1^.

|  | | | **N** | **Initial BW (g)** | | **Final BW (g)** | | **ADG (g)** | | **ADFI (g)** | | **FCR (g/g)** | | **Mortality (%)** | | **EPEF** | |
| --- | --- | --- | --- | --- | --- | --- | --- | --- | --- | --- | --- | --- | --- | --- | --- | --- | --- |
|  |  |  |  | **LSmeans** | **SE** | **LSmeans** | **SE** | **LSmeans** | **SE** | **LSmeans** | **SE** | **LSmeans** | **SE** | **LSmeans** | **SE** | **LSmeans** | **SE** |
| **Treatment (TRT)** | | |  |  |  |  |  |  |  |  |  |  |  |  |  |  |  |
| BD | | | 24 | 42.9 | 0.31 | 2063.3 | 16.86 | 57.7 | 0.48 | 87.9 | 0.73 | 1.523 | 0.013 | 2.64 | 0.469 | 369.1 | 5.37 |
| PR | | | 24 | 42.5 | 0.31 | 2042.0 | 16.84 | 57.1 | 0.48 | 87.0 | 0.73 | 1.523 | 0.013 | 1.75 | 0.468 | 368.6 | 5.37 |
| PH | | | 24 | 42.6 | 0.31 | 2060.0 | 16.80 | 57.6 | 0.48 | 87.1 | 0.73 | 1.512 | 0.013 | 2.82 | 0.467 | 371.2 | 5.36 |
| *p-value* | | |  | *0.6932* | | *0.6326* | | *0.6323* | | *0.6143* | | *0.7210* | | *0.2343* | | *0.9356* | |
| **Genetic line (GL)** | | |  |  |  |  |  |  |  |  |  |  |  |  |  |  |  |
| X | | | 36 | 44.5 | 0.26 | 2232.0 | 18.84 | 62.6 | 0.54 | 93.6 | 0.80 | 1.497 | 0.015 | 2.53 | 0.524 | 407.7 | 6.01 |
| Y | | | 36 | 40.8 | 0.26 | 1878.2 | 18.84 | 52.4 | 0.54 | 81.1 | 0.80 | 1_._542 | 0.015 | 2.27 | 0.524 | 331.6 | 6.01 |
| *p-value* | | |  | *<0.0001* | | *<0.0001* | | *<0.0001* | | *<0.0001* | | *0.0467* | | *0.7754* | | *<0.0001* | |
| **Sex** | | |  |  |  |  |  |  |  |  |  |  |  |  |  |  |  |
| F | | | 36 | 42.4 | 0.26 | 1953.1 | 13.82 | 54.6 | 0.39 | 83.9 | 0.61 | 1.535 | 0.012 | 2.05 | 0.384 | 348.2 | 4.41 |
| M | | | 36 | 42.9 | 0.26 | 2157.1 | 13.82 | 60.4 | 0.39 | 90.7 | 0.61 | 1.503 | 0.012 | 2.76 | 0.384 | 391.2 | 4.41 |
| *p-value* | | |  | *0.1786* | | *<0.0001* | | *<0.0001* | | *<0.0001* | | *0.0207* | | *0.2003* | | *<0.0001* | |
| **Experiment (EXP)** | | |  |  |  |  |  |  |  |  |  |  |  |  |  |  |  |
| A | | | 24 | 41.3^b^ | 0.31 | 2114.3^a^ | 19.33 | 59.2^a^ | 0.55 | 93.1^a^ | 0.89 | 1.577^a^ | 0.018 | 1.55 | 0.538 | 371.5 | 6.16 |
| B | | | 24 | 42.1^b^ | 0.31 | 2064.6^b^ | 17.20 | 57.8^b^ | 0.49 | 88.1^b^ | 0.81 | 1.524^b^ | 0.017 | 2.41 | 0.478 | 368.3 | 5.49 |
| C | | | 24 | 44.5^a^ | 0.31 | 1986.4^c^ | 21.41 | 55.5^c^ | 0.61 | 80.8^c^ | 0.97 | 1.457^c^ | 0.019 | 3.25 | 0.596 | 369.3 | 6.83 |
| *p-value* | | |  | *<0.0001* | | *0.0013* | | *0.0013* | | *<0.0001* | | *0.0004* | | *0.1753* | | *0.9173* | |
| **TRT** | | **GL** |  |  |  |  |  |  |  |  |  |  |  |  |  |  |  |
| BD | | X | 12 | 45.0 | 0.44 | 2238.2 | 28.94 | 62.7 | 0.83 | 93.7 | 1.20 | 1.493 | 0.021 | 2.90 | 0.805 | 407.9 | 9.23 |
|  |  | Y | 12 | 40.7 | 0.44 | 1888.4 | 27.41 | 52.7 | 0.78 | 82.1 | 1.14 | 1.552 | 0.020 | 2.39 | 0.762 | 330.3 | 8.74 |
| PR | | X | 12 | 44.2 | 0.44 | 2217.7 | 26.02 | 62.1 | 0.74 | 93.6 | 1.09 | 1.508 | 0.019 | 1.08 | 0.724 | 407.9 | 8.30 |
|  |  | Y | 12 | 40.8 | 0.44 | 1866.2 | 27.11 | 52.1 | 0.77 | 80.4 | 1.13 | 1.539 | 0.020 | 2.42 | 0.754 | 329.4 | 8.64 |
| PH | | X | 12 | 44.3 | 0.44 | 2240.0 | 26.43 | 62.8 | 0.76 | 93.5 | 1.10 | 1.489 | 0.019 | 3.61 | 0.735 | 407.3 | 8.43 |
|  |  | Y | 12 | 40.9 | 0.44 | 1880.0 | 26.61 | 52.5 | 0.76 | 80.7 | 1.11 | 1.534 | 0.019 | 2.02 | 0.740 | 335.2 | 8.49 |
| *p-value* | | |  | *0.4816* | | *0.9741* | | *0.9735* | | *0.6812* | | *0.6819* | | *0.0911* | | *0.9009* | |
| **TRT** | | **Sex** |  |  |  |  |  |  |  |  |  |  |  |  |  |  |  |
| BD | | F | 12 | 42.6 | 0.44 | 1969.6 | 23.75 | 55.1 | 0.68 | 84.3 | 1.00 | 1.530 | 0.018 | 2.08 | 0.661 | 351.6 | 7.57 |
|  |  | M | 12 | 43.1 | 0.44 | 2157.0 | 23.93 | 60.4 | 0.68 | 91.4 | 1.00 | 1.516 | 0.018 | 3.21 | 0.666 | 386.7 | 7.63 |
| PR | | F | 12 | 42.2 | 0.44 | 1936.2 | 23.99 | 54.1 | 0.69 | 84.1 | 1.01 | 1.553 | 0.018 | 1.81 | 0.667 | 342.3 | 7.65 |
|  |  | M | 12 | 42.8 | 0.44 | 2147.7 | 23.77 | 60.1 | 0.68 | 89.9 | 1.00 | 1.494 | 0.018 | 1.69 | 0.661 | 395.0 | 7.58 |
| PH | | F | 12 | 42.4 | 0.44 | 1953.6 | 23.81 | 54.6 | 0.68 | 83.3 | 1.00 | 1.524 | 0.018 | 2.25 | 0.662 | 350.6 | 7.59 |
|  |  | M | 12 | 42.8 | 0.44 | 2166.5 | 23.79 | 60.7 | 0.68 | 90.9 | 1.00 | 1.500 | 0.018 | 3.38 | 0.662 | 391.9 | 7.59 |
| *p-value* | | |  | *0.9718* | | *0.8345* | | *0.8347* | | *0.6253* | | *0.3558* | | *0.5551* | | *0.5074* | |
| **GL** | | **Sex** |  |  |  |  |  |  |  |  |  |  |  |  |  |  |  |
| X | | F | 18 | 44.5 | 0.36 | 2119.4 | 23.21 | 59.3 | 0.66 | 90.4 | 0.98 | 1.523 | 0.017 | 2.80^a^ | 0.646 | 379.3^b^ | 7.40 |
|  |  | M | 18 | 44.5 | 0.36 | 2344.6 | 23.39 | 65.8 | 0.67 | 96.8 | 0.98 | 1.471 | 0.017 | 2.26^a^ | 0.651 | 436.2^a^ | 7.46 |
| Y | | F | 18 | 40.3 | 0.36 | 1786.8 | 25.28 | 49.8 | 0.72 | 77.5 | 1.06 | 1.547 | 0.019 | 1.30^b^ | 0.703 | 317.0^d^ | 8.06 |
|  |  | M | 18 | 41.3 | 0.36 | 1969.6 | 21.64 | 55.1 | 0.62 | 84.7 | 0.91 | 1.536 | 0.016 | 3.25^a^ | 0.602 | 346.2^c^ | 6.90 |
| *p-value* | | |  | *0.2232* | | *0.2852* | | *0.2850* | | *0.6351* | | *0.1323* | | *0.0264* | | *0.0313* | |
| **GL** | **Sex** | **TRT** |  |  |  |  |  |  |  |  |  |  |  |  |  |  |  |
| X | F | BD | 6 | 45.2 | 0.63 | 2134.4 | 38.02 | 59.8 | 1.09 | 90.3 | 1.57 | 1.510 | 0.0268 | 3.34 | 1.057 | 383.0 | 12.12 |
|  |  | PR | 6 | 44.2 | 0.63 | 2097.1 | 35.21 | 58.7 | 1.01 | 90.5 | 1.46 | 1.543 | 0.0249 | 1.92 | 0.979 | 373.7 | 11.23 |
|  |  | PH | 6 | 44.1 | 0.63 | 2126.8 | 35.01 | 59.5 | 1.00 | 90.3 | 1.45 | 1.517 | 0.0248 | 3.15 | 0.974 | 381.1 | 11.16 |
|  | M | BD | 6 | 44.8 | 0.63 | 2342.0 | 36.90 | 65.7 | 1.05 | 97.0 | 1.52 | 1.477 | 0.0260 | 2.46 | 1.026 | 432.9 | 11.76 |
|  |  | PR | 6 | 44.2 | 0.63 | 2338.4 | 35.25 | 65.6 | 1.01 | 96.7 | 1.46 | 1.474 | 0.0250 | 0.25 | 0.980 | 442.1 | 11.24 |
|  |  | PH | 6 | 44.5 | 0.63 | 2353.3 | 36.13 | 66.0 | 1.03 | 96.6 | 1.49 | 1.462 | 0.0255 | 4.07 | 1.005 | 433.5 | 11.52 |
| Y | F | BD | 6 | 40.1 | 0.63 | 1804.7 | 38.10 | 50.3 | 1.09 | 78.4 | 1.57 | 1.549 | 0.0268 | 0.83 | 1.060 | 320.1 | 12.15 |
|  |  | PR | 6 | 40.2 | 0.63 | 1775.4 | 37.80 | 49.5 | 1.08 | 77.7 | 1.56 | 1.562 | 0.0266 | 1.71 | 1.051 | 310.9 | 12.02 |
|  |  | PH | 6 | 40.8 | 0.63 | 1780.4 | 36.14 | 49.6 | 1.03 | 76.3 | 1.49 | 1.531 | 0.0256 | 1.35 | 1.005 | 320.1 | 11.53 |
|  | M | BD | 6 | 41.3 | 0.63 | 1972.0 | 34.87 | 55.1 | 1.00 | 85.9 | 1.44 | 1.555 | 0.0247 | 3.95 | 0.970 | 340.6 | 11.12 |
|  |  | PR | 6 | 41.4 | 0.63 | 1957.1 | 34.72 | 54.7 | 0.99 | 83.1 | 1.44 | 1.515 | 0.0246 | 3.13 | 0.966 | 347.8 | 11.07 |
|  |  | PH | 6 | 41.1 | 0.63 | 1979.6 | 35.24 | 55.3 | 1.01 | 85.1 | 1.46 | 1.538 | 0.0250 | 2.69 | 0.980 | 350.3 | 11.24 |
| *p-value* | | |  | *0.6113* | | *0.9439* | | *0.9439* | | *0.6821* | | *0.8117* | | *0.3836* | | *0.9477* | |

^1^Values are presented as least squares means (LSmeans). ^a–d^Within a column, values without a common superscript differ, P < 0.05. X and Y correspond to two fast growing commercial poultry genetic lines.

SE: Standard error. BD: Basal diet; PR: Basal diet + probiotic; PH: Basal diet + phytobiotic. F: female; M: male. BW: Body weight; ADG: Average daily gain; ADFI: Average daily feed intake; FCR: Feed conversion ratio; EPEF: European production efficiency factor.

**Supplementary Table S3:** Performance 0-7 days^1^.

|  | | | **N** | **Initial BW (g)** | | **Final BW (g)** | | **ADG (g)** | | **ADFI (g)** | | **FCR (g/g)** | | **Mortality (%)** | | **EPEF** | |
| --- | --- | --- | --- | --- | --- | --- | --- | --- | --- | --- | --- | --- | --- | --- | --- | --- | --- |
|  |  |  |  | **LSmeans** | **SE** | **LSmeans** | **SE** | **LSmeans** | **SE** | **LSmeans** | **SE** | **LSmeans** | **SE** | **LSmeans** | **SE** | **LSmeans** | **SE** |
| **Treatment (TRT)** | | |  |  |  |  |  |  |  |  |  |  |  |  |  |  |  |
| BD | | | 24 | 42.9 | 0.31 | 178.4 | 0.91 | 19.4 | 0.13 | 20.4 | 0.18 | 1.056 | 0.008 | 0.93 | 0.329 | 182.9 | 1.95 |
| PR | | | 24 | 42.5 | 0.31 | 179.4 | 0.91 | 19.5 | 0.13 | 20.3 | 0.18 | 1.043 | 0.008 | 0.84 | 0.329 | 186.5 | 1.95 |
| PH | | | 24 | 42.6 | 0.31 | 178.9 | 0.90 | 19.5 | 0.13 | 20.3 | 0.18 | 1.050 | 0.008 | 1.36 | 0.328 | 183.9 | 1.94 |
| *p-value* | | |  | *0.6932* | | *0.7366* | | *0.7368* | | *0.8873* | | *0.4955* | | *0.4894* | | *0.4025* | |
| **Genetic line (GL)** | | |  |  |  |  |  |  |  |  |  |  |  |  |  |  |  |
| X | | | 36 | 44.5 | 0.26 | 182.0 | 1.01 | 19.9 | 0.14 | 20.5 | 0.20 | 1.035 | 0.009 | 0.76 | 0.368 | 192.3 | 2.18 |
| Y | | | 36 | 40.8 | 0.26 | 175.9 | 1.01 | 19.0 | 0.14 | 20.2 | 0.20 | 1.064 | 0.009 | 1.33 | 0.368 | 176.6 | 2.18 |
| *p-value* | | |  | *<0.0001* | | *0.0007* | | *0.0007* | | *0.4283* | | *0.0652* | | *0.3691* | | *<0.0001* | |
| **Sex** | | |  |  |  |  |  |  |  |  |  |  |  |  |  |  |  |
| F | | | 36 | 42.4 | 0.26 | 177.5 | 0.75 | 19.3 | 0.11 | 20.5 | 0.15 | 1.068 | 0.007 | 0.91 | 0.270 | 179.9 | 1.6 |
| M | | | 36 | 42.9 | 0.26 | 180.3 | 0.75 | 19.7 | 0.11 | 20.2 | 0.15 | 1.031 | 0.007 | 1.18 | 0.270 | 189.0 | 1.6 |
| *p-value* | | |  | *0.1786* | | *0.0096* | | *0.0096* | | *0.1834* | | *0.0003* | | *0.4999* | | *0.0002* | |
| **Experiment (EXP)** | | |  |  |  |  |  |  |  |  |  |  |  |  |  |  |  |
| A | | | 24 | 41.3^b^ | 0.31 | 166.0^c^ | 1.07 | 17.6^c^ | 0.15 | 19.4^c^ | 0.22 | 1.100^a^ | 0.009 | 0.58 | 0.377 | 158.8^c^ | 2.24 |
| B | | | 24 | 42.1^b^ | 0.31 | 175.2^b^ | 0.97 | 18.9^b^ | 0.14 | 20.4^b^ | 0.20 | 1.078^a^ | 0.008 | 0.96 | 0.336 | 174.0^b^ | 1.99 |
| C | | | 24 | 44.5^a^ | 0.31 | 195.5^a^ | 1.18 | 21.8^a^ | 0.17 | 21.2^a^ | 0.24 | 0.971^b^ | 0.01 | 1.6 | 0.418 | 220.6^a^ | 2.48 |
| *p-value* | | |  | *<0.0001* | | *<0.0001* | | *<0.0001* | | *<0.0001* | | *<0.0001* | | *0.2903* | | *<0.0001* | |
| **TRT** | | **GL** |  |  |  |  |  |  |  |  |  |  |  |  |  |  |  |
| BD | | X | 12 | 45.0 | 0.44 | 181.8 | 1.53 | 19.9 | 0.22 | 20.7 | 0.30 | 1.046 | 0.014 | 0.74 | 0.565 | 190.4 | 3.35 |
|  |  | Y | 12 | 40.7 | 0.44 | 175.0 | 1.45 | 18.9 | 0.21 | 20.2 | 0.29 | 1.066 | 0.013 | 1.12 | 0.535 | 175.3 | 3.17 |
| PR | | X | 12 | 44.2 | 0.44 | 181.3 | 1.38 | 19.8 | 0.2 | 20.4 | 0.27 | 1.036 | 0.012 | 0.15 | 0.508 | 192.5 | 3.01 |
|  |  | Y | 12 | 40.8 | 0.44 | 177.5 | 1.44 | 19.3 | 0.21 | 20.2 | 0.28 | 1.05 | 0.013 | 1.54 | 0.529 | 180.6 | 3.14 |
| PH | | X | 12 | 44.3 | 0.44 | 182.7 | 1.40 | 20.0 | 0.2 | 20.4 | 0.28 | 1.023 | 0.013 | 1.40 | 0.516 | 194.0 | 3.06 |
|  |  | Y | 12 | 40.9 | 0.44 | 175.0 | 1.41 | 18.9 | 0.2 | 20.3 | 0.28 | 1.076 | 0.013 | 1.33 | 0.519 | 173.7 | 3.08 |
| *p-value* | | |  | *0.4816* | | *0.2687* | | *0.2677* | | *0.6184* | | *0.1925* | | *0.2820* | | *0.3125* | |
| **TRT** | | **Sex** |  |  |  |  |  |  |  |  |  |  |  |  |  |  |  |
| BD | | F | 12 | 42.6 | 0.44 | 178.2 | 1.26 | 19.4 | 0.18 | 20.8 | 0.25 | 1.077 | 0.011 | 1.25 | 0.464 | 178.6 | 2.75 |
|  |  | M | 12 | 43.1 | 0.44 | 178.7 | 1.27 | 19.4 | 0.18 | 20.1 | 0.25 | 1.035 | 0.011 | 0.61 | 0.467 | 187.2 | 2.77 |
| PR | | F | 12 | 42.2 | 0.44 | 177.1 | 1.27 | 19.2 | 0.18 | 20.2 | 0.25 | 1.053 | 0.011 | 0.65 | 0.468 | 182.3 | 2.78 |
|  |  | M | 12 | 42.8 | 0.44 | 181.7 | 1.26 | 19.9 | 0.18 | 20.4 | 0.25 | 1.032 | 0.011 | 1.04 | 0.464 | 190.7 | 2.75 |
| PH | | F | 12 | 42.4 | 0.44 | 177.2 | 1.26 | 19.2 | 0.18 | 20.5 | 0.25 | 1.073 | 0.011 | 0.84 | 0.465 | 178.7 | 2.76 |
|  |  | M | 12 | 42.8 | 0.44 | 180.5 | 1.26 | 19.7 | 0.18 | 20.1 | 0.25 | 1.026 | 0.011 | 1.88 | 0.464 | 189.0 | 2.75 |
| *p-value* | | |  | *0.9718* | | *0.2511* | | *0.2516* | | *0.1355* | | *0.4740* | | *0.1970* | | *0.9276* | |
| **GL** | | **Sex** |  |  |  |  |  |  |  |  |  |  |  |  |  |  |  |
| X | | F | 18 | 44.5 | 0.36 | 180.9 | 1.23 | 19.7 | 0.18 | 20.6 | 0.24 | 1.050 | 0.011 | 0.90 | 0.453 | 188.5 | 2.69 |
|  |  | M | 18 | 44.5 | 0.36 | 183.0 | 1.24 | 20.1 | 0.18 | 20.4 | 0.25 | 1.020 | 0.011 | 0.62 | 0.457 | 196.1 | 2.71 |
| Y | | F | 18 | 40.3 | 0.36 | 174.2 | 1.34 | 18.8 | 0.19 | 20.4 | 0.26 | 1.085 | 0.012 | 0.93 | 0.493 | 171.2 | 2.92 |
|  |  | M | 18 | 41.3 | 0.36 | 177.6 | 1.15 | 19.3 | 0.16 | 20.1 | 0.23 | 1.042 | 0.010 | 1.73 | 0.422 | 181.9 | 2.50 |
| *p-value* | | |  | *0.2232* | | *0.5397* | | *0.5377* | | *0.7905* | | *0.4562* | | *0.1666* | | *0.5135* | |
| **GL** | **Sex** | **TRT** |  |  |  |  |  |  |  |  |  |  |  |  |  |  |  |
| X | F | BD | 6 | 45.2 | 0.63 | 182.1 | 2.00 | 19.9 | 0.29 | 20.9 | 0.39 | 1.059 | 0.0179 | 1.56 | 0.742 | 187.7 | 4.40 |
|  |  | PR | 6 | 44.2 | 0.63 | 179.0 | 1.86 | 19.5 | 0.27 | 20.2 | 0.36 | 1.044 | 0.0166 | 0.36 | 0.687 | 187.8 | 4.07 |
|  |  | PH | 6 | 44.1 | 0.63 | 181.6 | 1.85 | 19.8 | 0.26 | 20.7 | 0.36 | 1.046 | 0.0165 | 0.78 | 0.683 | 189.9 | 4.05 |
|  | M | BD | 6 | 44.8 | 0.63 | 181.6 | 1.94 | 19.8 | 0.28 | 20.4 | 0.38 | 1.033 | 0.0174 | -0.09 | 0.720 | 193.2 | 4.27 |
|  |  | PR | 6 | 44.2 | 0.63 | 183.6 | 1.86 | 20.1 | 0.27 | 20.6 | 0.36 | 1.027 | 0.0166 | -0.06 | 0.688 | 197.1 | 4.08 |
|  |  | PH | 6 | 44.5 | 0.63 | 183.9 | 1.90 | 20.2 | 0.27 | 20.1 | 0.37 | 1.001 | 0.0170 | 2.02 | 0.705 | 198.1 | 4.18 |
| Y | F | BD | 6 | 40.1 | 0.63 | 174.3 | 2.01 | 18.8 | 0.29 | 20.6 | 0.39 | 1.095 | 0.0180 | 0.94 | 0.744 | 169.4 | 4.41 |
|  |  | PR | 6 | 40.2 | 0.63 | 175.3 | 1.99 | 18.9 | 0.28 | 20.1 | 0.39 | 1.062 | 0.0178 | 0.94 | 0.738 | 176.8 | 4.37 |
|  |  | PH | 6 | 40.8 | 0.63 | 172.9 | 1.91 | 18.6 | 0.27 | 20.4 | 0.37 | 1.100 | 0.0170 | 0.91 | 0.705 | 167.5 | 4.18 |
|  | M | BD | 6 | 41.3 | 0.63 | 175.7 | 1.84 | 19.0 | 0.26 | 19.7 | 0.36 | 1.037 | 0.0164 | 1.30 | 0.681 | 181.2 | 4.03 |
|  |  | PR | 6 | 41.4 | 0.63 | 179.8 | 1.83 | 19.6 | 0.26 | 20.3 | 0.36 | 1.038 | 0.0164 | 2.13 | 0.678 | 184.3 | 4.02 |
|  |  | PH | 6 | 41.1 | 0.63 | 177.2 | 1.86 | 19.2 | 0.27 | 20.2 | 0.36 | 1.052 | 0.0166 | 1.75 | 0.688 | 180.0 | 4.08 |
| *p-value* | | |  | *0.6113* | | *0.8955* | | *0.8942* | | *0.7976* | | *0.7934* | | *0.3898* | | *0.7461* | |

^1^Values are presented as least squares means (LSmeans). ^a–d^Within a column, values without a common superscript differ, P < 0.05. X and Y correspond to two fast growing commercial poultry genetic lines.

SE: Standard error. BD: Basal diet; PR: Basal diet + probiotic; PH: Basal diet + phytobiotic. F: female; M: male. BW: Body weight; ADG: Average daily gain; ADFI: Average daily feed intake; FCR: Feed conversion ratio; EPEF: European production efficiency factor.

**Supplementary Table S4:** Performance 7-21 days^1^.

|  | | | **N** | **Initial BW(g)** | | **Final BW (g)** | | **ADG (g)** | | **ADFI (g)** | | **FCR (g/g)** | | **Mortality (%)** | | **EPEF** | |
| --- | --- | --- | --- | --- | --- | --- | --- | --- | --- | --- | --- | --- | --- | --- | --- | --- | --- |
|  |  |  |  | **LSmeans** | **SE** | **LSmeans** | **SE** | **LSmeans** | **SE** | **LSmeans** | **SE** | **LSmeans** | **SE** | **LSmeans** | **SE** | **LSmeans** | **SE** |
| **Treatment (TRT)** | | |  |  |  |  |  |  |  |  |  |  |  |  |  |  |  |
| BD | | | 24 | 178.4 | 0.91 | 898.1 | 5.01 | 51.4 | 0.32 | 70.2 | 1.06 | 1.367 | 0.0205 | 0.79 | 0.291 | 374.9 | 5.79 |
| PR | | | 24 | 179.4 | 0.91 | 890.6 | 5.01 | 50.8 | 0.32 | 70.1 | 1.06 | 1.380 | 0.0205 | 0.38 | 0.291 | 368.7 | 5.79 |
| PH | | | 24 | 178.9 | 0.90 | 897.6 | 4.99 | 51.3 | 0.32 | 69.4 | 1.06 | 1.353 | 0.0204 | 0.74 | 0.291 | 378.0 | 5.78 |
| *p-value* | | |  | *0.7366* | | *0.5002* | | *0.3502* | | *0.7723* | | *0.4920* | | *0.5408* | | *0.3937* | |
| **Genetic line (GL)** | | |  |  |  |  |  |  |  |  |  |  |  |  |  |  |  |
| X | | | 36 | 182.0 | 1.01 | 970.2 | 5.60 | 56.3 | 0.36 | 75.8 | 1.14 | 1.341 | 0.0220 | 0.96 | 0.323 | 415.3 | 6.29 |
| Y | | | 36 | 175.9 | 1.01 | 820.7 | 5.60 | 46.1 | 0.36 | 64.0 | 1.14 | 1.392 | 0.0220 | 0.32 | 0.323 | 332.4 | 6.29 |
| *p-value* | | |  | *0.0007* | | *<0.0001* | | *<0.0001* | | *<0.0001* | | *0.1035* | | *0.2359* | | *<0.0001* | |
| **Sex** | | |  |  |  |  |  |  |  |  |  |  |  |  |  |  |  |
| F | | | 36 | 177.5 | 0.75 | 869.5 | 4.11 | 49.4 | 0.26 | 67.3 | 0.95 | 1.363 | 0.0183 | 0.51 | 0.244 | 362.7 | 5.07 |
| M | | | 36 | 180.3 | 0.75 | 921.3 | 4.11 | 52.9 | 0.26 | 72.4 | 0.95 | 1.371 | 0.0183 | 0.77 | 0.244 | 385.0 | 5.07 |
| *p-value* | | |  | *0.0096* | | *<0.0001* | | *<0.0001* | | *<0.0001* | | *0.6784* | | *0.4287* | | *0.0003* | |
| **Experiment (EXP)** | | |  |  |  |  |  |  |  |  |  |  |  |  |  |  |  |
| A | | | 24 | 166.0^c^ | 1.07 | 868.1^c^ | 5.75 | 50.1^b^ | 0.37 | 65.5^b^ | 1.49 | 1.309 | 0.0287 | 0.08 | 0.352 | 384.7 | 7.78 |
| B | | | 24 | 175.2^b^ | 0.97 | 898.4^b^ | 5.11 | 51.7^a^ | 0.33 | 71.8^a^ | 1.42 | 1.390 | 0.0274 | 1.02 | 0.320 | 368.0 | 7.35 |
| C | | | 24 | 195.5^a^ | 1.18 | 919.7^a^ | 6.37 | 51.7^a^ | 0.41 | 72.4^a^ | 1.56 | 1.402 | 0.0300 | 0.81 | 0.384 | 368.9 | 8.23 |
| *p-value* | | |  | *<0.0001* | | *<0.0001* | | *0.0055* | | *0.0036* | | *0.0651* | | *0.1263* | | *0.2474* | |
| **TRT** | | **GL** |  |  |  |  |  |  |  |  |  |  |  |  |  |  |  |
| BD | | X | 12 | 181.8 | 1.53 | 974.9 | 8.61 | 56.6 | 0.55 | 75.7 | 1.57 | 1.330 | 0.0304 | 1.01 | 0.486 | 419.8 | 8.95 |
|  |  | Y | 12 | 175.0 | 1.45 | 821.3 | 8.15 | 46.2 | 0.52 | 64.6 | 1.50 | 1.404 | 0.0291 | 0.56 | 0.461 | 330.1 | 8.54 |
| PR | | X | 12 | 181.3 | 1.38 | 962.9 | 7.74 | 55.8 | 0.49 | 76.7 | 1.44 | 1.371 | 0.0279 | 0.59 | 0.439 | 406.0 | 8.16 |
|  |  | Y | 12 | 177.5 | 1.44 | 818.3 | 8.06 | 45.8 | 0.52 | 63.4 | 1.49 | 1.390 | 0.0288 | 0.18 | 0.457 | 331.3 | 8.46 |
| PH | | X | 12 | 182.7 | 1.40 | 972.8 | 7.86 | 56.4 | 0.50 | 74.9 | 1.46 | 1.323 | 0.0282 | 1.28 | 0.445 | 420.2 | 8.27 |
|  |  | Y | 12 | 175.0 | 1.41 | 822.4 | 7.91 | 46.2 | 0.51 | 63.9 | 1.46 | 1.383 | 0.0284 | 0.20 | 0.448 | 335.7 | 8.32 |
| *p-value* | | |  | *0.2687* | | *0.8137* | | *0.8897* | | *0.5361* | | *0.4683* | | *0.6329* | | *0.5525* | |
| **TRT** | | **Sex** |  |  |  |  |  |  |  |  |  |  |  |  |  |  |  |
| BD | | F | 12 | 178.2 | 1.26 | 874.5 | 7.06 | 49.7 | 0.45 | 66.8 | 1.34 | 1.343 | 0.0259 | 0.42 | 0.402 | 370.1 | 7.55 |
|  |  | M | 12 | 178.7 | 1.27 | 921.7 | 7.12 | 53.1 | 0.46 | 73.6 | 1.35 | 1.391 | 0.0261 | 1.15 | 0.405 | 379.7 | 7.60 |
| PR | | F | 12 | 177.1 | 1.27 | 863.8 | 7.13 | 49.0 | 0.46 | 68.6 | 1.35 | 1.399 | 0.0262 | 0.30 | 0.406 | 352.2 | 7.62 |
|  |  | M | 12 | 181.7 | 1.26 | 917.4 | 7.07 | 52.6 | 0.45 | 71.5 | 1.34 | 1.361 | 0.0260 | 0.47 | 0.402 | 385.1 | 7.56 |
| PH | | F | 12 | 177.2 | 1.26 | 870.3 | 7.08 | 49.5 | 0.45 | 66.6 | 1.34 | 1.346 | 0.0260 | 0.81 | 0.403 | 365.7 | 7.57 |
|  |  | M | 12 | 180.5 | 1.26 | 924.8 | 7.07 | 53.2 | 0.45 | 72.2 | 1.34 | 1.360 | 0.0260 | 0.68 | 0.403 | 390.2 | 7.56 |
| *p-value* | | |  | *0.2511* | | *0.8547* | | *0.9421* | | *0.2427* | | *0.1737* | | *0.5425* | | *0.2381* | |
| **GL** | | **Sex** |  |  |  |  |  |  |  |  |  |  |  |  |  |  |  |
| X | | F | 18 | 180.9 | 1.23 | 939.6 | 6.90 | 54.2 | 0.44 | 72.9 | 1.32 | 1.340 | 0.0255 | 1.17^a^ | 0.393 | 400.5 | 7.42 |
|  |  | M | 18 | 183.0 | 1.24 | 1000.7 | 6.96 | 58.4 | 0.44 | 78.7 | 1.33 | 1.343 | 0.0257 | 0.74^ab^ | 0.396 | 430.2 | 7.47 |
| Y | | F | 18 | 174.2 | 1.34 | 799.4 | 7.52 | 44.7 | 0.48 | 61.8 | 1.41 | 1.386 | 0.0273 | -0.16^b^ | 0.427 | 324.9 | 7.97 |
|  |  | M | 18 | 177.6 | 1.15 | 841.9 | 6.44 | 47.5 | 0.41 | 66.2 | 1.25 | 1.399 | 0.0242 | 0.79^a^ | 0.368 | 339.9 | 7.01 |
| *p-value* | | |  | *0.5397* | | *0.1163* | | *0.0617* | | *0.4591* | | *0.7983* | | *0.0390* | | *0.2013* | |
| **GL** | **Sex** | **TRT** |  |  |  |  |  |  |  |  |  |  |  |  |  |  |  |
| X | F | BD | 6 | 182.1 | 2.00 | 947.1 | 11.30 | 54.6 | 0.72 | 72.1 | 1.98 | 1.314 | 0.0384 | 1.05 | 0.635 | 410.5 | 11.46 |
|  |  | PR | 6 | 179.0 | 1.86 | 925.8 | 10.47 | 53.3 | 0.67 | 74.3 | 1.85 | 1.388 | 0.0359 | 0.79 | 0.589 | 383.6 | 10.67 |
|  |  | PH | 6 | 181.6 | 1.85 | 945.9 | 10.41 | 54.6 | 0.67 | 72.1 | 1.84 | 1.318 | 0.0357 | 1.67 | 0.586 | 407.3 | 10.62 |
|  | M | BD | 6 | 181.6 | 1.94 | 1002.7 | 10.97 | 58.7 | 0.70 | 79.3 | 1.93 | 1.347 | 0.0374 | 0.96 | 0.616 | 429.0 | 11.14 |
|  |  | PR | 6 | 183.6 | 1.86 | 999.9 | 10.48 | 58.3 | 0.67 | 79.2 | 1.85 | 1.353 | 0.0359 | 0.38 | 0.589 | 428.4 | 10.68 |
|  |  | PH | 6 | 183.9 | 1.90 | 999.6 | 10.74 | 58.3 | 0.69 | 77.7 | 1.89 | 1.328 | 0.0367 | 0.89 | 0.604 | 433.1 | 10.93 |
| Y | F | BD | 6 | 174.3 | 2.01 | 801.8 | 11.33 | 44.8 | 0.72 | 61.4 | 1.98 | 1.373 | 0.0385 | -0.21 | 0.636 | 329.8 | 11.48 |
|  |  | PR | 6 | 175.3 | 1.99 | 801.7 | 11.24 | 44.7 | 0.72 | 62.9 | 1.97 | 1.411 | 0.0382 | -0.20 | 0.631 | 320.8 | 11.39 |
|  |  | PH | 6 | 172.9 | 1.91 | 794.7 | 10.75 | 44.4 | 0.69 | 61.0 | 1.89 | 1.374 | 0.0367 | -0.06 | 0.604 | 324.1 | 10.93 |
|  | M | BD | 6 | 175.7 | 1.84 | 840.7 | 10.37 | 47.5 | 0.66 | 67.9 | 1.83 | 1.434 | 0.0356 | 1.34 | 0.583 | 330.4 | 10.58 |
|  |  | PR | 6 | 179.8 | 1.83 | 834.9 | 10.32 | 46.8 | 0.66 | 63.9 | 1.83 | 1.369 | 0.0354 | 0.56 | 0.581 | 341.8 | 10.53 |
|  |  | PH | 6 | 177.2 | 1.86 | 850.0 | 10.48 | 48.1 | 0.67 | 66.8 | 1.85 | 1.392 | 0.0359 | 0.46 | 0.589 | 347.3 | 10.68 |
| *p-value* | | |  | *0.8955* | | *0.3292* | | *0.2888* | | *0.6690* | | *0.9266* | | *0.9532* | | *0.7353* | |

^1^Values are presented as least squares means (LSmeans). ^a–d^Within a column, values without a common superscript differ, P < 0.05. X and Y correspond to two fast growing commercial poultry genetic lines.

SE: Standard error. BD: Basal diet; PR: Basal diet + probiotic; PH: Basal diet + phytobiotic. F: female; M: male. BW: Body weight; ADG: Average daily gain; ADFI: Average daily feed intake; FCR: Feed conversion ratio; EPEF: European production efficiency factor.

**Supplementary Table S5:** Performance 21-35 days^1^.

|  | | | **N** | **Initial BW (g)** | | | **Final BW (g)** | | **ADG (g)** | | **ADFI (g)** | | **FCR (g/g)** | | **Mortality (%)** | | **EPEF** | |
| --- | --- | --- | --- | --- | --- | --- | --- | --- | --- | --- | --- | --- | --- | --- | --- | --- | --- | --- |
|  |  |  |  | **LSmeans** | | **SE** | **LSmeans** | **SE** | **LSmeans** | **SE** | **LSmeans** | **SE** | **LSmeans** | **SE** | **LSmeans** | **SE** | **LSmeans** | **SE** |
| **Treatment (TRT)** | | |  |  | |  |  |  |  |  |  |  |  |  |  |  |  |  |
| BD | | | 24 | 898.1 | | 5.01 | 2063.3 | 16.86 | 83.2 | 1.06 | 143.3 | 1.65 | 1.719 | 0.022 | 1.15 | 0.508 | 480.1 | 10.53 |
| PR | | | 24 | 890.6 | | 5.01 | 2042.0 | 16.84 | 82.2 | 1.06 | 140.9 | 1.64 | 1.711 | 0.022 | 0.69 | 0.507 | 480.1 | 10.52 |
| PH | | | 24 | 897.6 | | 4.99 | 2060.0 | 16.80 | 83.0 | 1.06 | 142.0 | 1.64 | 1.710 | 0.022 | 0.90 | 0.507 | 482.9 | 10.50 |
| *p-value* | | |  | *0.5002* | | | *0.6326* | | *0.7787* | | *0.6045* | | *0.9385* | | *0.7090* | | *0.9757* | |
| **Genetic line (GL)** | | |  |  | |  |  |  |  |  |  |  |  |  |  |  |  |  |
| X | | | 36 | 970.2 | | 5.60 | 2232.0 | 18.84 | 90.1 | 1.18 | 151.9 | 1.84 | 1.691 | 0.024 | 1.04 | 0.545 | 531.4 | 11.77 |
| Y | | | 36 | 820.7 | | 5.60 | 1878.2 | 18.84 | 75.5 | 1.18 | 132.3 | 1.84 | 1.736 | 0.024 | 0.78 | 0.545 | 430.7 | 11.77 |
| *p-value* | | |  | *<0.0001* | | | *<0.0001* | | *<0.0001* | | *<0.0001* | | *0.2331* | | *0.7358* | | *<0.0001* | |
| **Sex** | | |  |  | |  |  |  |  |  |  |  |  |  |  |  |  |  |
| F | | | 36 | 869.5 | | 4.11 | 1953.1 | 13.82 | 77.4 | 0.88 | 136.1 | 1.35 | 1.750 | 0.019 | 0.80 | 0.455 | 439.6 | 8.64 |
| M | | | 36 | 921.3 | | 4.11 | 2157.1 | 13.82 | 88.3 | 0.88 | 148.0 | 1.35 | 1.677 | 0.019 | 1.02 | 0.455 | 522.5 | 8.64 |
| *p-value* | | |  | *<0.0001* | | | *<0.0001* | | *<0.0001* | | *<0.0001* | | *0.0023* | | *0.6295* | | *<0.0001* | |
| **Experiment (EXP)** | | |  |  | |  |  |  |  |  |  |  |  |  |  |  |  |  |
| A | | | 24 | 868.1^c^ | | 5.75 | 2114.3^a^ | 19.33 | 89.0^a^ | 1.26 | 162.4^a^ | 1.89 | 1.834^a^ | 0.029 | 1.04 | 0.717 | 486.2 | 12.08 |
| B | | | 24 | 898.4^b^ | | 5.11 | 2064.6^b^ | 17.20 | 83.3^b^ | 1.13 | 141.7^b^ | 1.68 | 1.700^b^ | 0.027 | 0.53 | 0.687 | 487.3 | 10.75 |
| C | | | 24 | 919.7^a^ | | 6.37 | 1986.4^c^ | 21.41 | 76.2^c^ | 1.38 | 122.2^c^ | 2.09 | 1.607^c^ | 0.030 | 1.17 | 0.748 | 469.5 | 13.38 |
| *p-value* | | |  | *<0.0001* | | | *0.0013* | | *<0.0001* | | *<0.0001* | | *<0.0001* | | *0.7893* | | *0.6146* | |
| **TRT** | | **GL** |  |  | |  |  |  |  |  |  |  |  |  |  |  |  |  |
| BD | | X | 12 | 974.9 | | 8.61 | 2238.2 | 28.94 | 90.2 | 1.8 | 152.3 | 2.83 | 1.694 | 0.035 | 1.44 | 0.748 | 529.1 | 18.08 |
|  |  | Y | 12 | 821.3 | | 8.15 | 1888.4 | 27.41 | 76.2 | 1.71 | 134.3 | 2.68 | 1.744 | 0.033 | 0.86 | 0.715 | 431.0 | 17.13 |
| PR | | X | 12 | 962.9 | | 7.74 | 2217.7 | 26.02 | 89.6 | 1.62 | 150.7 | 2.54 | 1.687 | 0.032 | 0.47 | 0.686 | 533.1 | 16.26 |
|  |  | Y | 12 | 818.3 | | 8.06 | 1866.2 | 27.11 | 74.9 | 1.69 | 131.2 | 2.65 | 1.736 | 0.033 | 0.91 | 0.709 | 427.1 | 16.94 |
| PH | | X | 12 | 972.8 | | 7.86 | 2240.0 | 26.43 | 90.5 | 1.65 | 152.7 | 2.58 | 1.693 | 0.032 | 1.21 | 0.695 | 531.9 | 16.51 |
|  |  | Y | 12 | 822.4 | | 7.91 | 1880.0 | 26.61 | 75.5 | 1.66 | 131.4 | 2.60 | 1.728 | 0.032 | 0.59 | 0.699 | 433.9 | 16.63 |
| *p-value* | | |  | *0.8137* | | | *0.9741* | | *0.9425* | | *0.7739* | | *0.9494* | | *0.5563* | | *0.9534* | |
| **TRT** | | **Sex** |  |  | |  |  |  |  |  |  |  |  |  |  |  |  |  |
| BD | | F | 12 | 874.5 | | 7.06 | 1969.6 | 23.75 | 78.2 | 1.48 | 137.7 | 2.32 | 1.751 | 0.029 | 0.49 | 0.640 | 444.8 | 14.84 |
|  |  | M | 12 | 921.7 | | 7.12 | 2157.0 | 23.93 | 88.2 | 1.49 | 148.9 | 2.34 | 1.688 | 0.029 | 1.81 | 0.644 | 515.4 | 14.95 |
| PR | | F | 12 | 863.8 | | 7.13 | 1936.2 | 23.99 | 76.6 | 1.50 | 135.4 | 2.34 | 1.757 | 0.029 | 1.14 | 0.645 | 432.1 | 14.99 |
|  |  | M | 12 | 917.4 | | 7.07 | 2147.7 | 23.77 | 87.9 | 1.48 | 146.5 | 2.32 | 1.666 | 0.029 | 0.24 | 0.640 | 528.1 | 14.85 |
| PH | | F | 12 | 870.3 | | 7.08 | 1953.6 | 23.81 | 77.4 | 1.49 | 135.3 | 2.33 | 1.742 | 0.029 | 0.78 | 0.641 | 441.9 | 14.88 |
|  |  | M | 12 | 924.8 | | 7.07 | 2166.5 | 23.79 | 88.7 | 1.48 | 148.8 | 2.32 | 1.679 | 0.029 | 1.02 | 0.641 | 524.0 | 14.86 |
| *p-value* | | |  | *0.8547* | | | *0.8345* | | *0.8804* | | *0.8525* | | *0.8411* | | *0.1448* | | *0.6938* | |
| **GL** | | **Sex** |  |  | |  |  |  |  |  |  |  |  |  |  |  |  |  |
| X | | F | 18 | 939.6 | | 6.90 | 2119.4 | 23.21 | 84.3 | 1.45 | 147.0 | 2.27 | 1.745 | 0.029 | 0.93 | 0.630 | 481.1 | 14.50 |
|  |  | M | 18 | 1000.7 | | 6.96 | 2344.6 | 23.39 | 96.0 | 1.46 | 156.9 | 2.29 | 1.638 | 0.029 | 1.15 | 0.633 | 581.7 | 14.62 |
| Y | | F | 18 | 799.4 | | 7.52 | 1786.8 | 25.28 | 70.5 | 1.58 | 125.3 | 2.47 | 1.755 | 0.031 | 0.67 | 0.672 | 398.1 | 15.80 |
|  |  | M | 18 | 841.9 | | 6.44 | 1969.6 | 21.64 | 80.6 | 1.35 | 139.2 | 2.11 | 1.717 | 0.027 | 0.90 | 0.598 | 463.3 | 13.52 |
| *p-value* | | |  | *0.1163* | | | *0.2852* | | *0.4861* | | *0.2958* | | *0.1325* | | *0.9926* | | *0.1548* | |
| **GL** | **Sex** | **TRT** |  |  | |  |  |  |  |  |  |  |  |  |  |  |  |  |
| X | F | BD | 6 | 947.1 | 11.30 | | 2134.4 | 38.02 | 84.8 | 2.35 | 147.5 | 3.71 | 1.740 | 0.045 | 0.86 | 0.943 | 485.6 | 23.76 |
|  |  | PR | 6 | 925.8 | 10.47 | | 2097.1 | 35.21 | 83.7 | 2.18 | 145.7 | 3.44 | 1.741 | 0.042 | 1.00 | 0.881 | 478.1 | 22.00 |
|  |  | PH | 6 | 945.9 | 10.41 | | 2126.8 | 35.01 | 84.3 | 2.17 | 147.8 | 3.42 | 1.753 | 0.042 | 0.93 | 0.877 | 479.5 | 21.88 |
|  | M | BD | 6 | 1002.7 | 10.97 | | 2342.0 | 36.90 | 95.7 | 2.29 | 157.1 | 3.60 | 1.648 | 0.044 | 2.02 | 0.918 | 572.6 | 23.05 |
|  |  | PR | 6 | 999.9 | 10.48 | | 2338.4 | 35.25 | 95.6 | 2.18 | 155.8 | 3.44 | 1.632 | 0.042 | -0.07 | 0.882 | 588.1 | 22.02 |
|  |  | PH | 6 | 999.6 | 10.74 | | 2353.3 | 36.13 | 96.7 | 2.24 | 157.7 | 3.53 | 1.633 | 0.043 | 1.50 | 0.901 | 584.4 | 22.57 |
| Y | F | BD | 6 | 801.8 | 11.33 | | 1804.7 | 38.10 | 71.6 | 2.36 | 127.8 | 3.72 | 1.761 | 0.045 | 0.12 | 0.944 | 404.0 | 23.81 |
|  |  | PR | 6 | 801.7 | 11.24 | | 1775.4 | 37.80 | 69.6 | 2.34 | 125.2 | 3.69 | 1.773 | 0.045 | 1.27 | 0.938 | 386.0 | 23.62 |
|  |  | PH | 6 | 794.7 | 10.75 | | 1780.4 | 36.14 | 70.4 | 2.24 | 122.9 | 3.53 | 1.731 | 0.043 | 0.63 | 0.902 | 404.2 | 22.58 |
|  | M | BD | 6 | 840.7 | 10.37 | | 1972.0 | 34.87 | 80.8 | 2.16 | 140.7 | 3.41 | 1.727 | 0.041 | 1.60 | 0.874 | 458.1 | 21.79 |
|  |  | PR | 6 | 834.9 | 10.32 | | 1957.1 | 34.72 | 80.2 | 2.15 | 137.2 | 3.39 | 1.700 | 0.041 | 0.55 | 0.870 | 468.1 | 21.70 |
|  |  | PH | 6 | 850.0 | 10.48 | | 1979.6 | 35.24 | 80.7 | 2.18 | 139.8 | 3.44 | 1.724 | 0.042 | 0.55 | 0.882 | 463.6 | 22.02 |
| *p-value* | | |  | *0.3292* | | | *0.9439* | | *0.9922* | | *0.8588* | | *0.7714* | | *0.8769* | | *0.9561* | |

^1^Values are presented as least squares means (LSmeans). ^a–d^Within a column, values without a common superscript differ, P < 0.05. X and Y correspond to two fast growing commercial poultry genetic lines.

SE: Standard error. BD: Basal diet; PR: Basal diet + probiotic; PH: Basal diet + phytobiotic. F: female; M: male. BW: Body weight; ADG: Average daily gain; ADFI: Average daily feed intake; FCR: Feed conversion ratio; EPEF: European production efficiency factor.

**Supplementary Table S6:** Concentration of corticosterone in feathers (pg/mg)^1^.

|  | | | **N** | **Corticosterone 0-35 days** | | **N** | **Corticosterone day 7** | | **Corticosterone day 21** | | **Corticosterone day 35** | |
| --- | --- | --- | --- | --- | --- | --- | --- | --- | --- | --- | --- | --- |
|  |  |  |  | **LSmeans** | **SE** |  | **LSmeans** | **SE** | **LSmeans** | **SE** | **LSmeans** | **SE** |
| **Treatment (TRT)** | | |  |  |  |  |  |  |  |  |  |  |
| BD | | | 72 | 12.18 | 0.962 | 24 | 9.80^b^ | 0.625 | 11.81 | 1.129 | 14.94 | 1.749 |
| PR | | | 72 | 13.09 | 0.962 | 24 | 10.59^ab^ | 0.625 | 11.43 | 1.129 | 17.27 | 1.749 |
| PH | | | 72 | 12.89 | 0.962 | 24 | 11.59^a^ | 0.625 | 12.77 | 1.129 | 14.31 | 1.749 |
| *p-value* | | |  | *0.4325* | |  | *0.0347* | | *0.4555* | | *0.2032* | |
| **Genetic line (GL)** | | |  |  |  |  |  |  |  |  |  |  |
| X | | | 108 | 13.91 | 0.914 | 36 | 9.91 | 0.562 | 13.66 | 1.037 | 18.15 | 1.601 |
| Y | | | 108 | 11.54 | 0.914 | 36 | 11.41 | 0.562 | 10.35 | 1.037 | 12.86 | 1.601 |
| p-value | | |  | *0.0001* | |  | *0.0082* | | *0.0005* | | *0.0004* | |
| **Sex** | | |  |  |  |  |  |  |  |  |  |  |
| F | | | 108 | 11.68 | 0.914 | 36 | 9.73 | 0.562 | 11.40 | 1.037 | 13.92 | 1.601 |
| M | | | 108 | 13.76 | 0.914 | 36 | 11.59 | 0.562 | 12.61 | 1.037 | 17.09 | 1.601 |
| p-value | | |  | *0.0008* | |  | *0.0013* | | *0.1811* | | *0.0281* | |
| **Experiment (EXP)** | | |  |  |  |  |  |  |  |  |  |  |
| A | | | 72 | 13.08 | 1.496 | 24 | 12.62^a^ | 0.850 | 11.67 | 1.621 | 14.96 | 2.491 |
| B | | | 72 | 13.95 | 1.496 | 24 | 11.13^a^ | 0.850 | 14.25 | 1.621 | 16.46 | 2.491 |
| C | | | 72 | 11.14 | 1.496 | 24 | 8.22^b^ | 0.850 | 10.08 | 1.621 | 15.10 | 2.491 |
| *p-value* | | |  | *0.3989* | |  | *0.0021* | | *0.1950* | | *0.8958* | |
| **TRT** | | **GL** |  |  |  |  |  |  |  |  |  |  |
| BD | | X | 36 | 13.41 | 1.093 | 12 | 8.60 | 0.785 | 13.46 | 1.369 | 18.17 | 2.131 |
|  |  | Y | 36 | 10.96 | 1.093 | 12 | 11.00 | 0.785 | 10.15 | 1.369 | 11.71 | 2.131 |
| PR | | X | 36 | 14.22 | 1.093 | 12 | 10.26 | 0.785 | 12.22 | 1.369 | 20.18 | 2.131 |
|  |  | Y | 36 | 11.97 | 1.093 | 12 | 10.91 | 0.785 | 10.64 | 1.369 | 14.36 | 2.131 |
| PH | | X | 36 | 14.09 | 1.093 | 12 | 10.86 | 0.785 | 15.29 | 1.369 | 16.11 | 2.131 |
|  |  | Y | 36 | 11.69 | 1.093 | 12 | 12.32 | 0.785 | 10.25 | 1.369 | 12.50 | 2.131 |
| *P-value* | | |  | *0.9890* | |  | *0.4363* | | *0.2944* | | *0.6880* | |
| **TRT** | | **Sex** |  |  |  |  |  |  |  |  |  |  |
| BD | | F | 36 | 10.53 | 1.093 | 12 | 9.00 | 0.785 | 9.26^b^ | 1.369 | 13.34 | 2.131 |
|  |  | M | 36 | 13.84 | 1.093 | 12 | 10.61 | 0.785 | 14.36^a^ | 1.369 | 16.54 | 2.131 |
| PR | | F | 36 | 12.15 | 1.093 | 12 | 9.27 | 0.785 | 11.57^ab^ | 1.369 | 15.60 | 2.131 |
|  |  | M | 36 | 14.04 | 1.093 | 12 | 11.91 | 0.785 | 11.28^ab^ | 1.369 | 18.93 | 2.131 |
| PH | | F | 36 | 12.36 | 1.093 | 12 | 10.92 | 0.785 | 13.36^a^ | 1.369 | 12.81 | 2.131 |
|  |  | M | 36 | 13.41 | 1.093 | 12 | 12.26 | 0.785 | 12.18^ab^ | 1.369 | 15.80 | 2.131 |
| *P-value* | | |  | *0.3042* | |  | *0.5939* | | *0.0120* | | *0.9949* | |
| **GL** | | **Sex** |  |  |  |  |  |  |  |  |  |  |
| X | | F | 54 | 12.37 | 1.008 | 18 | 9.85^b^ | 0.682 | 12.74 | 1.215 | 14.53 | 1.885 |
|  |  | M | 54 | 15.44 | 1.008 | 18 | 9.96^b^ | 0.682 | 14.57 | 1.215 | 21.78 | 1.885 |
| Y | | F | 54 | 10.99 | 1.008 | 18 | 9.61^b^ | 0.682 | 10.05 | 1.215 | 13.31 | 1.885 |
|  |  | M | 54 | 12.09 | 1.008 | 18 | 13.21^a^ | 0.682 | 10.64 | 1.215 | 12.40 | 1.885 |
| *P-value* | | |  | *0.1030* | |  | *0.0023* | | *0.4878* | | *0.0053* | |
| **GL** | **Sex** | **TRT** |  |  |  |  |  |  |  |  |  |  |
| X | F | BD | 18 | 11.14 | 1.317 | 6 | 8.58 | 1.032 | 10.03 | 1.753 | 14.82 | 2.740 |
|  |  | PR | 18 | 12.51 | 1.317 | 6 | 8.63 | 1.032 | 16.89 | 1.753 | 21.53 | 2.740 |
|  |  | PH | 18 | 13.47 | 1.317 | 6 | 9.42 | 1.032 | 8.49 | 1.753 | 11.86 | 2.740 |
|  | M | BD | 18 | 15.68 | 1.317 | 6 | 12.58 | 1.032 | 11.82 | 1.753 | 11.56 | 2.740 |
|  |  | PR | 18 | 15.93 | 1.317 | 6 | 10.17 | 1.032 | 12.50 | 1.753 | 14.85 | 2.740 |
|  |  | PH | 18 | 14.71 | 1.317 | 6 | 10.35 | 1.032 | 11.93 | 1.753 | 25.51 | 2.740 |
| Y | F | BD | 18 | 9.92 | 1.317 | 6 | 8.36 | 1.032 | 10.64 | 1.753 | 16.36 | 2.740 |
|  |  | PR | 18 | 11.79 | 1.317 | 6 | 13.46 | 1.032 | 10.64 | 1.753 | 12.36 | 2.740 |
|  |  | PH | 18 | 11.26 | 1.317 | 6 | 10.81 | 1.032 | 15.68 | 1.753 | 13.91 | 2.740 |
|  | M | BD | 18 | 11.99 | 1.317 | 6 | 10.91 | 1.032 | 14.90 | 1.753 | 18.31 | 2.740 |
|  |  | PR | 18 | 12.15 | 1.317 | 6 | 11.04 | 1.032 | 11.03 | 1.753 | 11.71 | 2.740 |
|  |  | PH | 18 | 12.12 | 1.317 | 6 | 13.60 | 1.032 | 9.46 | 1.753 | 13.29 | 2.740 |
| *P-value* | | |  | *0.6362* | |  | *0.6353* | | *0.6390* | | *0.2286* | |
| **Day** | | |  |  |  |  |  |  |  |  |  |  |
| 7 | | | 72 | 10.66^b^ | 0.96 |  |  |  |  |  |  |  |
| 21 | | | 72 | 12.00^b^ | 0.96 |  |  |  |  |  |  |  |
| 35 | | | 72 | 15.51^a^ | 0.96 |  |  |  |  |  |  |  |
| *P-value* | | |  | *<0.0001* | |  |  |  |  |  |  |  |
| **TRT** | | **Day** |  |  |  |  |  |  |  |  |  |  |
| BD | | 7 | 24 | 9.80 | 1.210 |  |  |  |  |  |  |  |
|  |  | 21 | 24 | 11.81 | 1.210 |  |  |  |  |  |  |  |
|  |  | 35 | 24 | 14.94 | 1.210 |  |  |  |  |  |  |  |
| PR | | 7 | 24 | 10.59 | 1.210 |  |  |  |  |  |  |  |
|  |  | 21 | 24 | 11.43 | 1.210 |  |  |  |  |  |  |  |
|  |  | 35 | 24 | 17.27 | 1.210 |  |  |  |  |  |  |  |
| PH | | 7 | 24 | 11.59 | 1.210 |  |  |  |  |  |  |  |
|  |  | 21 | 24 | 12.77 | 1.210 |  |  |  |  |  |  |  |
|  |  | 35 | 24 | 14.31 | 1.210 |  |  |  |  |  |  |  |
| *P-value* | | |  | *0.1203* | |  |  |  |  |  |  |  |
| **GL** | | **Day** |  |  |  |  |  |  |  |  |  |  |
| X | | 7 | 36 | 9.91^d^ | 1.093 |  |  |  |  |  |  |  |
|  |  | 21 | 36 | 13.66^b^ | 1.093 |  |  |  |  |  |  |  |
|  |  | 35 | 36 | 18.15^a^ | 1.093 |  |  |  |  |  |  |  |
| Y | | 7 | 36 | 11.41^cd^ | 1.093 |  |  |  |  |  |  |  |
|  |  | 21 | 36 | 10.35^d^ | 1.093 |  |  |  |  |  |  |  |
|  |  | 35 | 36 | 12.86^bc^ | 1.093 |  |  |  |  |  |  |  |
| *P-value* | | |  | *<0.0001* | |  |  |  |  |  |  |  |
| **Sex** | | **Day** |  |  |  |  |  |  |  |  |  |  |
| F | | 7 | 36 | 9.73 | 1.093 |  |  |  |  |  |  |  |
|  |  | 21 | 36 | 11.40 | 1.093 |  |  |  |  |  |  |  |
|  |  | 35 | 36 | 13.92 | 1.093 |  |  |  |  |  |  |  |
| M | | 7 | 36 | 11.59 | 1.093 |  |  |  |  |  |  |  |
|  |  | 21 | 36 | 12.61 | 1.093 |  |  |  |  |  |  |  |
|  |  | 35 | 36 | 17.09 | 1.093 |  |  |  |  |  |  |  |
| *P-value* | | |  | *0.3997* | |  |  |  |  |  |  |  |

^1^Values are presented as least squares means (LSmeans). SE: Standard error. BD: Basal diet; PR: Basal diet + probiotic; PH: Basal diet + phytobiotic. F: female; M: male.

X and Y correspond to two fast growing commercial poultry genetic lines.

^a–d^Within a column, values without a common superscript differ, P < 0.05

**Supplementary Table S7a:** Concentration of C-reactive protein (CRP), chicken haptoglobin-like protein (PIT54) and Lipopolysaccharides (LPS) in blood 0-35 days^1^.

|  | | **N** | **CRP (pg/mL)** | | **PIT54 (ng/mL)** | | **LPS (ng/L)** | |
| --- | --- | --- | --- | --- | --- | --- | --- | --- |
|  |  |  | **LSmeans** | **SE** | **LSmeans** | **SE** | **LSmeans** | **SE** |
| **Treatment (TRT)** | |  |  |  |  |  |  |  |
| BD | | 72 | 2.40 | 0.18 | 1.01 | 0.15 | 25.43 | 2.72 |
| PR | | 72 | 2.57 | 0.18 | 1.11 | 0.15 | 24.79 | 2.72 |
| PH | | 72 | 2.47 | 0.18 | 1.26 | 0.15 | 25.20 | 2.72 |
| *p-value* | |  | *0.8031* | | *0.4608* | | *0.9258* | |
| **Genetic line (GL)** | |  |  |  |  |  |  |  |
| X | | 108 | 2.55 | 0.15 | 1.31 | 0.12 | 25.42 | 2.64 |
| Y | | 108 | 2.41 | 0.15 | 0.95 | 0.12 | 24.86 | 2.64 |
| *p-value* | |  | *0.4871* | | *0.0354* | | *0.6718* | |
| **Sex** | |  |  |  |  |  |  |  |
| F | | 108 | 2.44 | 0.15 | 1.09 | 0.12 | 25.18 | 2.64 |
| M | | 108 | 2.51 | 0.15 | 1.17 | 0.12 | 25.10 | 2.64 |
| *p-value* | |  | *0.7346* | | *0.6528* | | *0.9466* | |
| **Day** | |  |  |  |  |  |  |  |
| 7 | | 72 | 3.27^a^ | 0.18 | 2.10^a^ | 0.15 | 29.25^a^ | 2.72 |
| 21 | | 72 | 2.92^a^ | 0.18 | 0.59^b^ | 0.15 | 19.23^b^ | 2.72 |
| 35 | | 72 | 1.24^b^ | 0.18 | 0.70^b^ | 0.15 | 26.94^a^ | 2.72 |
| *p-value* | |  | *<0.0001* | | *<0.0001* | | *<0.0001* | |
| **Experiment (EXP)** | |  |  |  |  |  |  |  |
| A | | 72 | 1.28^c^ | 0.19 | 1.07^b^ | 0.15 | 15.06^b^ | 4.43 |
| B | | 72 | 2.06^b^ | 0.19 | 1.71^a^ | 0.15 | 35.15^a^ | 4.43 |
| C | | 72 | 4.10^a^ | 0.19 | 0.61^c^ | 0.15 | 25.21^ab^ | 4.43 |
| *p-value* | |  | *<0.0001* | | *<0.0001* | | *0.0073* | |
| **TRT** | **GL** |  |  |  |  |  |  |  |
| 1 | X | 36 | 2.61 | 0.25 | 1.11 | 0.21 | 26.69 | 2.96 |
|  | Y | 36 | 2.19 | 0.25 | 0.90 | 0.21 | 24.17 | 2.96 |
| 2 | X | 36 | 2.51 | 0.25 | 1.39 | 0.21 | 24.58 | 2.96 |
|  | Y | 36 | 2.62 | 0.25 | 0.84 | 0.21 | 25.01 | 2.96 |
| 3 | X | 36 | 2.52 | 0.25 | 1.42 | 0.21 | 25.00 | 2.96 |
|  | Y | 36 | 2.41 | 0.25 | 1.10 | 0.21 | 25.39 | 2.96 |
| *p-value* | |  | *0.5744* | | *0.7157* | | *0.5851* | |
| **TRT** | **Sex** |  |  |  |  |  |  |  |
| BD | F | 36 | 2.26 | 0.25 | 1.10 | 0.21 | 24.50 | 2.96 |
|  | M | 36 | 2.54 | 0.25 | 0.91 | 0.21 | 26.35 | 2.96 |
| PR | F | 36 | 2.50 | 0.25 | 0.95 | 0.21 | 26.28 | 2.96 |
|  | M | 36 | 2.63 | 0.25 | 1.27 | 0.21 | 23.31 | 2.96 |
| PH | F | 36 | 2.56 | 0.25 | 1.22 | 0.21 | 24.78 | 2.96 |
|  | M | 36 | 2.37 | 0.25 | 1.31 | 0.21 | 25.62 | 2.96 |
| *p-value* | |  | *0.6451* | | *0.4737* | | *0.3029* | |
| **GL** | **Sex** |  |  |  |  |  |  |  |
| X | F | 54 | 2.62 | 0.21 | 1.23 | 0.17 | 25.41 | 2.8 |
|  | M | 54 | 2.48 | 0.21 | 1.38 | 0.17 | 25.44 | 2.8 |
| Y | F | 54 | 2.27 | 0.21 | 0.95 | 0.17 | 24.96 | 2.8 |
|  | M | 54 | 2.54 | 0.21 | 0.95 | 0.17 | 24.75 | 2.8 |
| *p-value* | |  | *0.3259* | | *0.6622* | | *0.9315* | |
| **TRT** | **Day** |  |  |  |  |  |  |  |
| BD | 7 | 24 | 3.09 | 0.31 | 1.74 | 0.25 | 29.73 | 3.18 |
|  | 21 | 24 | 2.80 | 0.31 | 0.58 | 0.25 | 18.87 | 3.18 |
|  | 35 | 24 | 1.32 | 0.31 | 0.71 | 0.25 | 27.68 | 3.18 |
| PR | 7 | 24 | 3.31 | 0.31 | 2.20 | 0.25 | 28.05 | 3.18 |
|  | 21 | 24 | 3.17 | 0.31 | 0.56 | 0.25 | 20.59 | 3.18 |
|  | 35 | 24 | 1.23 | 0.31 | 0.58 | 0.25 | 25.74 | 3.18 |
| PH | 7 | 24 | 3.41 | 0.31 | 2.38 | 0.25 | 29.96 | 3.18 |
|  | 21 | 24 | 2.81 | 0.31 | 0.62 | 0.25 | 18.22 | 3.18 |
|  | 35 | 24 | 1.18 | 0.31 | 0.80 | 0.25 | 27.41 | 3.18 |
| *p-value* | |  | *0.8808* | | *0.6893* | | *0.7924* | |
| **GL** | **Day** |  |  |  |  |  |  |  |
| X | 7 | 36 | 3.54 | 0.25 | 2.56 | 0.21 | 28.74 | 2.96 |
|  | 21 | 36 | 2.89 | 0.25 | 0.67 | 0.21 | 20.65 | 2.96 |
|  | 35 | 36 | 1.21 | 0.25 | 0.70 | 0.21 | 26.88 | 2.96 |
| Y | 7 | 36 | 2.99 | 0.25 | 1.65 | 0.21 | 29.76 | 2.96 |
|  | 21 | 36 | 2.96 | 0.25 | 0.50 | 0.21 | 17.80 | 2.96 |
|  | 35 | 36 | 1.27 | 0.25 | 0.69 | 0.21 | 27.01 | 2.96 |
| *p-value* | |  | *0.3632* | | *0.0757* | | *0.4658* | |
| **Sex** | **Day** |  |  |  |  |  |  |  |
| F | 7 | 36 | 3.17 | 0.25 | 2.01 | 0.21 | 28.90 | 2.96 |
|  | 21 | 36 | 2.85 | 0.25 | 0.59 | 0.21 | 19.45 | 2.96 |
|  | 35 | 36 | 1.31 | 0.25 | 0.68 | 0.21 | 27.21 | 2.96 |
| M | 7 | 36 | 3.37 | 0.25 | 2.20 | 0.21 | 29.60 | 2.96 |
|  | 21 | 36 | 2.99 | 0.25 | 0.58 | 0.21 | 19.00 | 2.96 |
|  | 35 | 36 | 1.18 | 0.25 | 0.71 | 0.21 | 26.68 | 2.96 |
| *p-value* | |  | *0.7807* | | *0.8717* | | *0.915* | |
| **TRT** | **EXP** |  |  |  |  |  |  |  |
| BD | A | 24 | 1.25 | 0.31 | 1.09 | 0.25 | 15.62 | 4.72 |
|  | B | 24 | 2.02 | 0.31 | 1.23 | 0.25 | 34.87 | 4.72 |
|  | C | 24 | 3.92 | 0.31 | 0.70 | 0.25 | 25.80 | 4.72 |
| PR | A | 24 | 1.37 | 0.31 | 1.00 | 0.25 | 15.27 | 4.72 |
|  | B | 24 | 1.96 | 0.31 | 1.84 | 0.25 | 34.32 | 4.72 |
|  | C | 24 | 4.36 | 0.31 | 0.51 | 0.25 | 24.80 | 4.72 |
| PH | A | 24 | 1.20 | 0.31 | 1.13 | 0.25 | 14.28 | 4.72 |
|  | B | 24 | 2.18 | 0.31 | 2.06 | 0.25 | 36.27 | 4.72 |
|  | C | 24 | 4.02 | 0.31 | 0.61 | 0.25 | 25.05 | 4.72 |
| *p-value* | |  | *0.8927* | | *0.3365* | | *0.9475* | |
| **GL** | **EXP** |  |  |  |  |  |  |  |
| X | A | 36 | 1.36 | 0.26 | 1.33 | 0.21 | 14.44 | 4.57 |
|  | B | 36 | 2.14 | 0.26 | 1.88 | 0.21 | 35.88 | 4.57 |
|  | C | 36 | 4.15 | 0.26 | 0.72 | 0.21 | 25.95 | 4.57 |
| Y | A | 36 | 1.20 | 0.26 | 0.81 | 0.21 | 15.67 | 4.57 |
|  | B | 36 | 1.98 | 0.26 | 1.54 | 0.21 | 34.42 | 4.57 |
|  | C | 36 | 4.05 | 0.26 | 0.49 | 0.21 | 24.48 | 4.57 |
| *p-value* | |  | *0.9926* | | *0.7714* | | *0.6357* | |
| **Sex** | **EXP** |  |  |  |  |  |  |  |
| F | A | 36 | 1.32 | 0.26 | 1.14 | 0.21 | 15.45 | 4.57 |
|  | B | 36 | 1.93 | 0.26 | 1.51 | 0.21 | 35.25 | 4.57 |
|  | C | 36 | 4.07 | 0.26 | 0.63 | 0.21 | 24.86 | 4.57 |
| M | A | 36 | 1.23 | 0.26 | 1.00 | 0.21 | 14.66 | 4.57 |
|  | B | 36 | 2.18 | 0.26 | 1.91 | 0.21 | 35.05 | 4.57 |
|  | C | 36 | 4.13 | 0.26 | 0.58 | 0.21 | 25.57 | 4.57 |
| *p-value* | |  | *0.7838* | | *0.3832* | | *0.898* | |
| **Day** | **EXP** |  |  |  |  |  |  |  |
| 7 | A | 24 | 1.47^c^ | 0.31 | 2.09^b^ | 0.25 | 11.72^d^ | 4.72 |
|  | B | 24 | 3.90^b^ | 0.31 | 3.48^a^ | 0.25 | 46.70^a^ | 4.72 |
|  | C | 24 | 4.43^b^ | 0.31 | 0.74^c^ | 0.25 | 29.33^b^ | 4.72 |
| 21 | A | 24 | 1.11^c^ | 0.31 | 0.54^c^ | 0.25 | 12.83^cd^ | 4.72 |
|  | B | 24 | 1.14^c^ | 0.31 | 0.72^c^ | 0.25 | 25.33^bc^ | 4.72 |
|  | C | 24 | 6.52^a^ | 0.31 | 0.50^c^ | 0.25 | 19.52^cd^ | 4.72 |
| 35 | A | 24 | 1.25 | 0.31 | 0.58^c^ | 0.25 | 20.62^bc^ | 4.72 |
|  | B | 24 | 1.12^c^ | 0.31 | 0.93^c^ | 0.25 | 33.43^b^ | 4.72 |
|  | C | 24 | 1.36^c^ | 0.31 | 0.58^c^ | 0.25 | 26.79^b^ | 4.72 |
| *p-value* | |  | *<0.0001* | | *<0.0001* | | *<0.0001* | |

^1^Values are presented as least squares means (LSmeans). SE: Standard error.

BD: Basal diet; PR: Basal diet + probiotic; PH: Basal diet + phytobiotic. F: female; M: male.

X and Y correspond to two fast growing commercial poultry genetic lines.

^a–d^Within a column, values without a common superscript differ, P < 0.05.

**Supplementary Table S7b:** Concentration of C-reactive protein (CRP), chicken haptoglobin-like protein (PIT54) and Lipopolysaccharides (LPS) in blood per day^1^.

|  | | | **N** | **CRP d7 (pg/mL)** | | **CRP d21 (pg/mL)** | | **CRP d35 (pg/mL)** | | **PIT54 d7 (ng/ml)** | | **PIT54 d21 (ng/ml)** | | **PIT54 d35 (ng/ml)** | | **LPS d7 (ng/L)** | | **LPS d21 (ng/L)** | | **LPS d 35 (ng/L)** | |
| --- | --- | --- | --- | --- | --- | --- | --- | --- | --- | --- | --- | --- | --- | --- | --- | --- | --- | --- | --- | --- | --- |
|  |  |  |  | **LS** | **SE** | **LS** | **SE** | **LS** | **SE** | **LS** | **SE** | **LS** | **SE** | **LS** | **SE** | **LS** | **SE** | **LS** | **SE** | **LS** | **SE** |
| **Treatment (TRT)** | | |  |  |  |  |  |  |  |  |  |  |  |  |  |  |  |  |  |  |  |
| BD | | | 24 | 3.09 | 0.22 | 2.80 | 0.57 | 1.32 | 0.08 | 1.74 | 0.42 | 0.58 | 0.04 | 0.71 | 0.08 | 29.73 | 2.96 | 18.87 | 2.38 | 27.68 | 5.95 |
| PR | | | 24 | 3.31 | 0.22 | 3.17 | 0.57 | 1.23 | 0.08 | 2.20 | 0.42 | 0.56 | 0.04 | 0.58 | 0.08 | 28.05 | 2.96 | 20.59 | 2.38 | 25.74 | 5.95 |
| PH | | | 24 | 3.41 | 0.22 | 2.81 | 0.57 | 1.18 | 0.08 | 2.38 | 0.42 | 0.62 | 0.04 | 0.80 | 0.08 | 29.96 | 2.96 | 18.22 | 2.38 | 27.41 | 5.95 |
| *P-value* | | |  | *0.5406* | | *0.7581* | | *0.2094* | | *0.5406* | | *0.6850* | | *0.1543* | | *0.1774* | | *0.5088* | | *0.7140* | |
| **Genetic line (GL)** | | |  |  |  |  |  |  |  |  |  |  |  |  |  |  |  |  |  |  |  |
| X | | | 36 | 3.54 | 0.18 | 2.89 | 0.53 | 1.21 | 0.08 | 2.56 | 0.34 | 0.67 | 0.04 | 0.70 | 0.06 | 28.74 | 2.93 | 20.65 | 2.23 | 26.88 | 5.86 |
| Y | | | 36 | 2.99 | 0.18 | 2.96 | 0.53 | 1.27 | 0.08 | 1.65 | 0.34 | 0.50 | 0.04 | 0.69 | 0.06 | 29.76 | 2.93 | 17.80 | 2.23 | 27.01 | 5.86 |
| *P-value* | | |  | *0.0687* | | *0.8870* | | *0.3300* | | *0.0687* | | *0.0010* | | *0.9519* | | *0.2657* | | *0.1007* | | *0.9477* | |
| **Sex** | | |  |  |  |  |  |  |  |  |  |  |  |  |  |  |  |  |  |  |  |
| F | | | 36 | 3.17 | 0.18 | 2.85 | 0.53 | 1.31 | 0.08 | 2.01 | 0.34 | 0.59 | 0.04 | 0.68 | 0.06 | 28.9 | 2.93 | 19.45 | 2.23 | 27.21 | 5.86 |
| M | | | 36 | 3.37 | 0.18 | 2.99 | 0.53 | 1.18 | 0.08 | 2.20 | 0.34 | 0.58 | 0.04 | 0.71 | 0.06 | 29.6 | 2.93 | 19.00 | 2.23 | 26.68 | 5.86 |
| *P-value* | | |  | *0.6824* | | *0.7601* | | *0.0454* | | *0.6824* | | *0.9344* | | *0.7171* | | *0.4384* | | *0.7964* | | *0.8000* | |
| **Experiment (EXP)** | | |  |  |  |  |  |  |  |  |  |  |  |  |  |  |  |  |  |  |  |
| A | | | 24 | 1.47^b^ | 0.24 | 1.11^b^ | 0.82 | 1.25 | 0.12 | 2.09^b^ | 0.42 | 0.54^b^ | 0.04 | 0.58^b^ | 0.08 | 11.72^c^ | 5.01 | 12.83 | 3.56 | 20.62 | 9.99 |
| B | | | 24 | 3.90^a^ | 0.24 | 1.14^b^ | 0.82 | 1.12 | 0.12 | 3.48^a^ | 0.42 | 0.72^a^ | 0.04 | 0.93^a^ | 0.08 | 46.70^a^ | 5.01 | 25.33 | 3.56 | 33.43 | 9.99 |
| C | | | 24 | 4.43^a^ | 0.24 | 6.52^a^ | 0.82 | 1.36 | 0.12 | 0.74^c^ | 0.42 | 0.50^b^ | 0.04 | 0.58^b^ | 0.08 | 29.33^b^ | 5.01 | 19.52 | 3.56 | 26.79 | 9.99 |
| *P-value* | | |  | *0.0001* | | *<0.0001* | | *0.4057* | | *0.0001* | | *0.0012* | | *0.0032* | | *<0.0001* | | *0.0535* | | *0.6646* | |
| **TRT** | | **GL** |  |  |  |  |  |  |  |  |  |  |  |  |  |  |  |  |  |  |  |
| BD | | X | 12 | 3.49 | 0.3 | 3.11 | 0.7 | 1.23 | 0.1 | 1.88 | 0.59 | 0.65 | 0.06 | 0.81 | 0.11 | 29.99^a^ | 3.06 | 20.22 | 2.8 | 29.86 | 6.22 |
|  |  | Y | 12 | 2.68 | 0.3 | 2.49 | 0.7 | 1.40 | 0.1 | 1.59 | 0.59 | 0.50 | 0.06 | 0.60 | 0.11 | 29.47^a^ | 3.06 | 17.52 | 2.8 | 25.51 | 6.22 |
| PR | | X | 12 | 3.55 | 0.3 | 2.73 | 0.7 | 1.25 | 0.1 | 2.95 | 0.59 | 0.65 | 0.06 | 0.56 | 0.11 | 25.74^b^ | 3.06 | 22.11 | 2.8 | 25.88 | 6.22 |
|  |  | Y | 12 | 3.06 | 0.3 | 3.60 | 0.7 | 1.20 | 0.1 | 1.45 | 0.59 | 0.48 | 0.06 | 0.60 | 0.11 | 30.36^a^ | 3.06 | 19.06 | 2.8 | 25.61 | 6.22 |
| PH | | X | 12 | 3.59 | 0.3 | 2.84 | 0.7 | 1.14 | 0.1 | 2.84 | 0.59 | 0.71 | 0.06 | 0.72 | 0.11 | 30.49^a^ | 3.06 | 19.62 | 2.8 | 24.89 | 6.22 |
|  |  | Y | 12 | 3.23 | 0.3 | 2.79 | 0.7 | 1.22 | 0.1 | 1.92 | 0.59 | 0.52 | 0.06 | 0.88 | 0.11 | 29.44^a^ | 3.06 | 16.82 | 2.8 | 29.92 | 6.22 |
| *P-value* | | |  | *0.5998* | | *0.4133* | | *0.3487* | | *0.5998* | | *0.9325* | | *0.2629* | | *0.0235* | | *0.9965* | | *0.1919* | |
| **TRT** | | **Sex** |  |  |  |  |  |  |  |  |  |  |  |  |  |  |  |  |  |  |  |
| BD | | F | 12 | 2.71 | 0.3 | 2.64 | 0.7 | 1.44 | 0.1 | 2 | 0.59 | 0.62 | 0.06 | 0.69 | 0.11 | 30.52 | 3.06 | 16.79 | 2.8 | 26.2 | 6.22 |
|  |  | M | 12 | 3.46 | 0.3 | 2.95 | 0.7 | 1.20 | 0.1 | 1.48 | 0.59 | 0.54 | 0.06 | 0.73 | 0.11 | 28.95 | 3.06 | 20.95 | 2.8 | 29.17 | 6.22 |
| PR | | F | 12 | 3.36 | 0.3 | 2.89 | 0.7 | 1.27 | 0.1 | 1.80 | 0.59 | 0.54 | 0.06 | 0.52 | 0.11 | 27.72 | 3.06 | 21.53 | 2.8 | 29.58 | 6.22 |
|  |  | M | 12 | 3.25 | 0.3 | 3.44 | 0.7 | 1.18 | 0.1 | 2.60 | 0.59 | 0.59 | 0.06 | 0.64 | 0.11 | 28.39 | 3.06 | 19.64 | 2.8 | 21.91 | 6.22 |
| PH | | F | 12 | 3.44 | 0.3 | 3.03 | 0.7 | 1.22 | 0.1 | 2.21 | 0.59 | 0.61 | 0.06 | 0.83 | 0.11 | 28.46 | 3.06 | 20.01 | 2.8 | 25.85 | 6.22 |
|  |  | M | 12 | 3.38 | 0.3 | 2.59 | 0.7 | 1.15 | 0.1 | 2.54 | 0.59 | 0.62 | 0.06 | 0.77 | 0.11 | 31.47 | 3.06 | 16.43 | 2.8 | 28.96 | 6.22 |
| *P-value* | | |  | *0.5366* | | *0.6567* | | *0.4872* | | *0.5366* | | *0.5615* | | *0.7481* | | *0.1260* | | *0.1606* | | *0.0613* | |
| **GL** | | **Sex** |  |  |  |  |  |  |  |  |  |  |  |  |  |  |  |  |  |  |  |
| X | | F | 18 | 3.49 | 0.25 | 3.10 | 0.62 | 1.25 | 0.09 | 2.32 | 0.49 | 0.68 | 0.05 | 0.70 | 0.09 | 28.11 | 3 | 20.89 | 2.53 | 27.23 | 6.04 |
|  |  | M | 18 | 3.60 | 0.25 | 2.68 | 0.62 | 1.17 | 0.09 | 2.79 | 0.49 | 0.66 | 0.05 | 0.70 | 0.09 | 29.37 | 3 | 20.42 | 2.53 | 26.52 | 6.04 |
| Y | | F | 18 | 2.84 | 0.25 | 2.60 | 0.62 | 1.36 | 0.09 | 1.69 | 0.49 | 0.49 | 0.05 | 0.66 | 0.09 | 29.68 | 3 | 18.01 | 2.53 | 27.19 | 6.04 |
|  |  | M | 18 | 3.13 | 0.25 | 3.31 | 0.62 | 1.18 | 0.09 | 1.62 | 0.49 | 0.50 | 0.05 | 0.73 | 0.09 | 29.83 | 3 | 17.59 | 2.53 | 26.84 | 6.04 |
| *P-value* | | |  | *0.5776* | | *0.2219* | | *0.4209* | | *0.5776* | | *0.8065* | | *0.6819* | | *0.5440* | | *0.9883* | | *0.9311* | |
| **GL** | **Sex** | **TRT** |  |  |  |  |  |  |  |  |  |  |  |  |  |  |  |  |  |  |  |
| X | F | BD | 6 | 3.14 | 0.42 | 3.44 | 0.89 | 1.29 | 0.13 | 2.30 | 0.84 | 0.72 | 0.09 | 0.78 | 0.16 | 31.13 | 3.26 | 16.20 | 3.5 | 29.58 | 6.72 |
|  |  | PR | 6 | 3.77 | 0.42 | 2.91 | 0.89 | 1.33 | 0.13 | 2.59 | 0.84 | 0.61 | 0.09 | 0.53 | 0.16 | 25.38 | 3.26 | 22.34 | 3.5 | 29.48 | 6.72 |
|  |  | PH | 6 | 3.56 | 0.42 | 2.97 | 0.89 | 1.13 | 0.13 | 2.07 | 0.84 | 0.71 | 0.09 | 0.78 | 0.16 | 27.83 | 3.26 | 24.12 | 3.5 | 22.63 | 6.72 |
|  | M | BD | 6 | 3.84 | 0.42 | 2.78 | 0.89 | 1.18 | 0.13 | 1.46 | 0.84 | 0.58 | 0.09 | 0.84 | 0.16 | 28.85 | 3.26 | 24.25 | 3.5 | 30.13 | 6.72 |
|  |  | PR | 6 | 3.33 | 0.42 | 2.56 | 0.89 | 1.18 | 0.13 | 3.30 | 0.84 | 0.69 | 0.09 | 0.60 | 0.16 | 26.11 | 3.26 | 21.88 | 3.5 | 22.28 | 6.72 |
|  |  | PH | 6 | 3.62 | 0.42 | 2.71 | 0.89 | 1.15 | 0.13 | 3.61 | 0.84 | 0.72 | 0.09 | 0.66 | 0.16 | 33.15 | 3.26 | 15.12 | 3.5 | 27.15 | 6.72 |
| Y | F | BD | 6 | 2.28 | 0.42 | 1.85 | 0.89 | 1.59 | 0.13 | 1.69 | 0.84 | 0.51 | 0.09 | 0.59 | 0.16 | 29.90 | 3.26 | 17.39 | 3.5 | 22.81 | 6.72 |
|  |  | PR | 6 | 2.94 | 0.42 | 2.87 | 0.89 | 1.20 | 0.13 | 1.02 | 0.84 | 0.46 | 0.09 | 0.51 | 0.16 | 30.05 | 3.26 | 20.73 | 3.5 | 29.67 | 6.72 |
|  |  | PH | 6 | 3.31 | 0.42 | 3.09 | 0.89 | 1.30 | 0.13 | 2.36 | 0.84 | 0.51 | 0.09 | 0.87 | 0.16 | 29.08 | 3.26 | 15.91 | 3.5 | 29.08 | 6.72 |
|  | M | BD | 6 | 3.08 | 0.42 | 3.12 | 0.89 | 1.22 | 0.13 | 1.50 | 0.84 | 0.50 | 0.09 | 0.62 | 0.16 | 29.04 | 3.26 | 17.64 | 3.5 | 28.21 | 6.72 |
|  |  | PR | 6 | 3.18 | 0.42 | 4.33 | 0.89 | 1.19 | 0.13 | 1.89 | 0.84 | 0.49 | 0.09 | 0.68 | 0.16 | 30.67 | 3.26 | 17.40 | 3.5 | 21.54 | 6.72 |
|  |  | PH | 6 | 3.15 | 0.42 | 2.48 | 0.89 | 1.14 | 0.13 | 1.47 | 0.84 | 0.53 | 0.09 | 0.89 | 0.16 | 29.79 | 3.26 | 17.74 | 3.5 | 30.76 | 6.72 |
| *P-value* | | |  | *0.3871* | | *0.5218* | | *0.4079* | | *0.3871* | | *0.7641* | | *0.9213* | | *0.3720* | | *0.0785* | | *0.7356* | |

^1^Values are presented as least squares means (LS). SE: Standard error. BD: Basal diet; PR: Basal diet + probiotic; PH: Basal diet + phytobiotic. F: female; M: male. X and Y correspond to two fast growing commercial poultry genetic lines.

^a–d^Within a column, values without a common superscript differ, P < 0.05.

**Supplementary Table S8a:** Punctuation of footpad dermatitis lesions (%) 0-35 days^1^.

|  | | **N** | **Punctuation 0** | | **Punctuation 1** | | **Punctuation 2** | | **Punctuation 3** | | **Punctuation 4** | |
| --- | --- | --- | --- | --- | --- | --- | --- | --- | --- | --- | --- | --- |
|  |  |  | **LSmeans** | **SE** | **LSmeans** | **SE** | **LSmeans** | **SE** | **LSmeans** | **SE** | **LSmeans** | **SE** |
| **Treatment (TRT)** | |  |  |  |  |  |  |  |  |  |  |  |
| BD | | 72 | 83.29 | 2.217 | 9.21 | 1.046 | 6.11 | 1.086 | 1.31 | 0.580 | 0.08 | 0.088 |
| PR | | 72 | 86.34 | 2.217 | 9.03 | 1.046 | 4.19 | 1.086 | 0.40 | 0.580 | 0.04 | 0.088 |
| PH | | 72 | 87.70 | 2.217 | 7.48 | 1.046 | 3.50 | 1.086 | 1.13 | 0.580 | 0.19 | 0.088 |
| *p-value* | |  | *0.0959* | | *0.2274* | | *0.0508* | | *0.3575* | | *0.3649* | |
| **Genetic line (GL)** | |  |  |  |  |  |  |  |  |  |  |  |
| X | | 108 | 81.70 | 2.051 | 10.19 | 0.946 | 6.25 | 0.989 | 1.65 | 0.512 | 0.21 | 0.076 |
| Y | | 108 | 89.86 | 2.051 | 6.95 | 0.946 | 2.94 | 0.989 | 0.24 | 0.512 | 0.00 | 0.076 |
| *p-value* | |  | *<0.0001* | | *0.0005* | | *0.0003* | | *0.0109* | | *0.0212* | |
| **Sex** | |  |  |  |  |  |  |  |  |  |  |  |
| F | | 108 | 85.56 | 2.051 | 8.56 | 0.946 | 4.91 | 0.989 | 0.88 | 0.512 | 0.10 | 0.076 |
| M | | 108 | 86.00 | 2.051 | 8.58 | 0.946 | 4.29 | 0.989 | 1.01 | 0.512 | 0.12 | 0.076 |
| *p-value* | |  | *0.7928* | | *0.9769* | | *0.4902* | | *0.8112* | | *0.8320* | |
| **Day** | |  |  |  |  |  |  |  |  |  |  |  |
| 7 | | 72 | 99.90^a^ | 2.217 | 0.11^c^ | 1.046 | 0.00^c^ | 1.086 | 0.00^b^ | 0.580 | 0.00 | 0.088 |
| 21 | | 72 | 85.08^b^ | 2.217 | 9.29^b^ | 1.046 | 4.38^b^ | 1.086 | 1.01^ab^ | 0.580 | 0.25 | 0.088 |
| 35 | | 72 | 72.36^c^ | 2.217 | 16.32^a^ | 1.046 | 9.42^a^ | 1.086 | 1.84^a^ | 0.580 | 0.06 | 0.088 |
| *p-value* | |  | *<0.0001* | | *<0.0001* | | *<0.0001* | | *0.0254* | | *0.0626* | |
| **Experiment (EXP)** | |  |  |  |  |  |  |  |  |  |  |  |
| A | | 72 | 73.82^b^ | 3.238 | 16.08^a^ | 1.442 | 8.84^a^ | 1.528 | 1.01 | 0.752 | 0.25 | 0.106 |
| B | | 72 | 94.08^a^ | 3.238 | 4.74^b^ | 1.442 | 1.13^b^ | 1.528 | 0.05 | 0.752 | 0.00 | 0.106 |
| C | | 72 | 89.44^a^ | 3.238 | 4.88^b^ | 1.442 | 3.83^b^ | 1.528 | 1.79 | 0.752 | 0.06 | 0.106 |
| *p-value* | |  | *<0.0001* | | *<0.0001* | | *0.0021* | | *0.2673* | | *0.2151* | |
| **TRT** | **GL** |  |  |  |  |  |  |  |  |  |  |  |
| BD | X | 36 | 79.13 | 2.655 | 10.94 | 1.303 | 7.65 | 1.334 | 2.12 | 0.748 | 0.16 | 0.118 |
|  | Y | 36 | 87.44 | 2.655 | 7.48 | 1.303 | 4.58 | 1.334 | 0.50 | 0.748 | 0.00 | 0.118 |
| PR | X | 36 | 82.61 | 2.655 | 10.61 | 1.303 | 6.02 | 1.334 | 0.67 | 0.748 | 0.08 | 0.118 |
|  | Y | 36 | 90.07 | 2.655 | 7.45 | 1.303 | 2.35 | 1.334 | 0.13 | 0.748 | 0.00 | 0.118 |
| PH | X | 36 | 83.34 | 2.655 | 9.02 | 1.303 | 5.09 | 1.334 | 2.17 | 0.748 | 0.39 | 0.118 |
|  | Y | 36 | 92.07 | 2.655 | 5.94 | 1.303 | 1.90 | 1.334 | 0.09 | 0.748 | 0.00 | 0.118 |
| *p-value* | |  | *0.9517* | | *0.9831* | | *0.9572* | | *0.4995* | | *0.3649* | |
| **TRT** | **Sex** |  |  |  |  |  |  |  |  |  |  |  |
| BD | F | 36 | 82.02 | 2.655 | 9.85 | 1.303 | 7.19 | 1.334 | 0.86 | 0.748 | 0.16 | 0.118 |
|  | M | 36 | 84.56 | 2.655 | 8.56 | 1.303 | 5.04 | 1.334 | 1.76 | 0.748 | 0.00 | 0.118 |
| PR | F | 36 | 87.90 | 2.655 | 8.63 | 1.303 | 3.48 | 1.334 | 0.00 | 0.748 | 0.08 | 0.118 |
|  | M | 36 | 84.78 | 2.655 | 9.43 | 1.303 | 4.89 | 1.334 | 0.80 | 0.748 | 0.00 | 0.118 |
| PH | F | 36 | 86.75 | 2.655 | 7.19 | 1.303 | 4.06 | 1.334 | 1.79 | 0.748 | 0.39 | 0.118 |
|  | M | 36 | 88.66 | 2.655 | 7.76 | 1.303 | 2.93 | 1.334 | 0.47 | 0.748 | 0.00 | 0.118 |
| *p-value* | |  | *0.3279* | | *0.5796* | | *0.2495* | | *0.1757* | | *0.8696* | |
| **GL** | **Sex** |  |  |  |  |  |  |  |  |  |  |  |
| X | F | 54 | 81.41 | 2.372 | 10.13 | 1.138 | 6.79 | 1.174 | 1.49 | 0.641 | 0.19 | 0.099 |
|  | M | 54 | 81.98 | 2.372 | 10.24 | 1.138 | 5.72 | 1.174 | 1.82 | 0.641 | 0.23 | 0.099 |
| Y | F | 54 | 89.70 | 2.372 | 6.99 | 1.138 | 3.03 | 1.174 | 0.28 | 0.641 | 0.00 | 0.099 |
|  | M | 54 | 90.02 | 2.372 | 6.92 | 1.138 | 2.85 | 1.174 | 0.20 | 0.641 | 0.00 | 0.099 |
| *p-value* | |  | *0.9398* | | *0.9208* | | *0.6227* | | *0.1999* | | *0.8320* | |
| **TRT** | **Day** |  |  |  |  |  |  |  |  |  |  |  |
| BD | 7 | 24 | 99.79 | 3.030 | 0.21 | 1.517 | 0.00 | 1.543 | 0.00 | 0.884 | 0.00 | 0.141 |
|  | 21 | 24 | 81.61 | 3.030 | 10.12 | 1.517 | 6.65 | 1.543 | 1.38 | 0.884 | 0.24 | 0.141 |
|  | 35 | 24 | 68.46 | 3.030 | 17.28 | 1.517 | 11.70 | 1.543 | 2.56 | 0.884 | 0.00 | 0.141 |
| PR | 7 | 24 | 99.89 | 3.030 | 0.11 | 1.517 | 0.00 | 1.543 | 0.00 | 0.884 | 0.00 | 0.141 |
|  | 21 | 24 | 85.10 | 3.030 | 10.49 | 1.517 | 3.67 | 1.543 | 0.62 | 0.884 | 0.13 | 0.141 |
|  | 35 | 24 | 74.03 | 3.030 | 16.50 | 1.517 | 8.89 | 1.543 | 0.59 | 0.884 | 0.00 | 0.141 |
| PH | 7 | 24 | 100.00 | 3.030 | 0.00 | 1.517 | 0.00 | 1.543 | 0.00 | 0.884 | 0.00 | 0.141 |
|  | 21 | 24 | 88.52 | 3.030 | 7.25 | 1.517 | 2.81 | 1.543 | 1.02 | 0.884 | 0.39 | 0.141 |
|  | 35 | 24 | 74.59 | 3.030 | 15.18 | 1.517 | 7.68 | 1.543 | 2.37 | 0.884 | 0.19 | 0.141 |
| *p-value* | |  | *0.6399* | | *0.7844* | | *0.5467* | | *0.7555* | | *0.8764* | |
| **GL** | **Day** |  |  |  |  |  |  |  |  |  |  |  |
| X | 7 | 36 | 99.86^a^ | 2.655 | 0.14^d^ | 1.303 | 0.00^c^ | 1.334 | 0.00^b^ | 0.748 | 0.00 | 0.118 |
|  | 21 | 36 | 81.75^c^ | 2.655 | 11.13^b^ | 1.303 | 5.19^b^ | 1.334 | 1.42^b^ | 0.748 | 0.51 | 0.118 |
|  | 35 | 36 | 63.48^d^ | 2.655 | 19.29^a^ | 1.303 | 13.57^a^ | 1.334 | 3.54^a^ | 0.748 | 0.13 | 0.118 |
| Y | 7 | 36 | 99.93^a^ | 2.655 | 0.07^d^ | 1.303 | 0.00^c^ | 1.334 | 0.00^b^ | 0.748 | 0.00 | 0.118 |
|  | 21 | 36 | 88.41^b^ | 2.655 | 7.44^c^ | 1.303 | 3.56^b^ | 1.334 | 0.59^b^ | 0.748 | 0.00 | 0.118 |
|  | 35 | 36 | 81.25^c^ | 2.655 | 13.35^b^ | 1.303 | 5.27^b^ | 1.334 | 0.13^b^ | 0.748 | 0.00 | 0.118 |
| *p-value* | |  | *0.0002* | | *0.0297* | | *0.0006* | | *0.0320* | | *0.0626* | |
| **Sex** | **Day** |  |  |  |  |  |  |  |  |  |  |  |
| F | 7 | 36 | 99.79 | 2.655 | 0.21 | 1.303 | 0.00 | 1.334 | 0.00 | 0.748 | 0.00 | 0.118 |
|  | 21 | 36 | 84.27 | 2.655 | 9.97 | 1.303 | 4.52 | 1.334 | 1.08 | 0.748 | 0.16 | 0.118 |
|  | 35 | 36 | 72.61 | 2.655 | 15.49 | 1.303 | 10.21 | 1.334 | 1.57 | 0.748 | 0.13 | 0.118 |
| M | 7 | 36 | 100.00 | 2.655 | 0.00 | 1.303 | 0.00 | 1.334 | 0.00 | 0.748 | 0.00 | 0.118 |
|  | 21 | 36 | 85.89 | 2.655 | 8.60 | 1.303 | 4.23 | 1.334 | 0.93 | 0.748 | 0.35 | 0.118 |
|  | 35 | 36 | 72.11 | 2.655 | 17.15 | 1.303 | 8.64 | 1.334 | 2.11 | 0.748 | 0.00 | 0.118 |
| *p-value* | |  | *0.8722* | | *0.3848* | | *0.7483* | | *0.8612* | | *0.3719* | |
| **TRT** | **EXP** |  |  |  |  |  |  |  |  |  |  |  |
| BD | A | 24 | 68.39 | 3.840 | 17.19 | 1.813 | 12.80^a^ | 1.881 | 1.38 | 1.005 | 0.24 | 0.153 |
|  | B | 24 | 93.68 | 3.840 | 4.91 | 1.813 | 1.25^c^ | 1.881 | 0.15 | 1.005 | 0.00 | 0.153 |
|  | C | 24 | 87.80 | 3.840 | 5.51 | 1.813 | 4.29^bc^ | 1.881 | 2.40 | 1.005 | 0.00 | 0.153 |
| PR | A | 24 | 72.83 | 3.840 | 17.57 | 1.813 | 8.86^b^ | 1.881 | 0.62 | 1.005 | 0.13 | 0.153 |
|  | B | 24 | 93.60 | 3.840 | 5.31 | 1.813 | 1.10^c^ | 1.881 | 0.00 | 1.005 | 0.00 | 0.153 |
|  | C | 24 | 92.60 | 3.840 | 4.21 | 1.813 | 2.60^c^ | 1.881 | 0.59 | 1.005 | 0.00 | 0.153 |
| PH | A | 24 | 80.25 | 3.840 | 13.49 | 1.813 | 4.86^bc^ | 1.881 | 1.02 | 1.005 | 0.39 | 0.153 |
|  | B | 24 | 94.95 | 3.840 | 4.01 | 1.813 | 1.04^c^ | 1.881 | 0.00 | 1.005 | 0.00 | 0.153 |
|  | C | 24 | 87.92 | 3.840 | 4.93 | 1.813 | 4.59^bc^ | 1.881 | 2.37 | 1.005 | 0.19 | 0.153 |
| *p-value* | |  | *0.0696* | | *0.4692* | | *0.0167* | | *0.8069* | | *0.8764* | |
| **GL** | **EXP** |  |  |  |  |  |  |  |  |  |  |  |
| X | A | 36 | 68.45^c^ | 3.552 | 18.46^a^ | 1.638 | 11.16^a^ | 1.713 | 1.42^ab^ | 0.887 | 0.51 | 0.131 |
|  | B | 36 | 94.33^a^ | 3.552 | 4.45^cd^ | 1.638 | 1.12^b^ | 1.713 | 0.10^b^ | 0.887 | 0.00 | 0.131 |
|  | C | 36 | 82.31^b^ | 3.552 | 7.65^c^ | 1.638 | 6.48^a^ | 1.713 | 3.44^a^ | 0.887 | 0.13 | 0.131 |
| Y | A | 36 | 79.19^b^ | 3.552 | 13.70^b^ | 1.638 | 6.52^a^ | 1.713 | 0.59^b^ | 0.887 | 0.00 | 0.131 |
|  | B | 36 | 93.82^a^ | 3.552 | 5.04^cd^ | 1.638 | 1.14^b^ | 1.713 | 0.00^b^ | 0.887 | 0.00 | 0.131 |
|  | C | 36 | 96.57^a^ | 3.552 | 2.12^d^ | 1.638 | 1.17^b^ | 1.713 | 0.13^b^ | 0.887 | 0.00 | 0.131 |
| *p-value* | |  | *0.0014* | | *0.0121* | | *0.0333* | | *0.0459* | | *0.0626* | |
| **Sex** | **EXP** |  |  |  |  |  |  |  |  |  |  |  |
| F | A | 36 | 72.43 | 3.552 | 16.74 | 1.638 | 9.59 | 1.713 | 1.08 | 0.887 | 0.16 | 0.131 |
|  | B | 36 | 94.57 | 3.552 | 4.41 | 1.638 | 1.01 | 1.713 | 0.00 | 0.887 | 0.00 | 0.131 |
|  | C | 36 | 89.66 | 3.552 | 4.52 | 1.638 | 4.12 | 1.713 | 1.57 | 0.887 | 0.13 | 0.131 |
| M | A | 36 | 75.21 | 3.552 | 15.43 | 1.638 | 8.09 | 1.713 | 0.93 | 0.887 | 0.35 | 0.131 |
|  | B | 36 | 93.58 | 3.552 | 5.08 | 1.638 | 1.25 | 1.713 | 0.10 | 0.887 | 0.00 | 0.131 |
|  | C | 36 | 89.22 | 3.552 | 5.25 | 1.638 | 3.53 | 1.713 | 2.01 | 0.887 | 0.00 | 0.131 |
| *p-value* | |  | *0.6161* | | *0.5756* | | *0.7336* | | *0.9060* | | *0.3719* | |
| **Day** | **EXP** |  |  |  |  |  |  |  |  |  |  |  |
| 7 | A | 24 | 99.69^a^ | 3.840 | 0.32^d^ | 1.813 | 0.00^b^ | 1.881 | 0.00^b^ | 1.005 | 0.00^b^ | 0.153 |
|  | B | 24 | 100.00^a^ | 3.840 | 0.00^d^ | 1.813 | 0.00^b^ | 1.881 | 0.00^b^ | 1.005 | 0.00^b^ | 0.153 |
|  | C | 24 | 100.00^a^ | 3.840 | 0.00^d^ | 1.813 | 0.00^b^ | 1.881 | 0.00^b^ | 1.005 | 0.00^b^ | 0.153 |
| 21 | A | 24 | 62.93^cd^ | 3.840 | 20.16^b^ | 1.813 | 13.13^a^ | 1.881 | 3.02^a^ | 1.005 | 0.76^a^ | 0.153 |
|  | B | 24 | 95.34^ab^ | 3.840 | 4.66^d^ | 1.813 | 0.00^b^ | 1.881 | 0.00^b^ | 1.005 | 0.00^b^ | 0.153 |
|  | C | 24 | 96.96^ab^ | 3.840 | 3.04^d^ | 1.813 | 0.00^b^ | 1.881 | 0.00^b^ | 1.005 | 0.00^b^ | 0.153 |
| 35 | A | 24 | 58.84^d^ | 3.840 | 27.77^a^ | 1.813 | 13.39^a^ | 1.881 | 0.00^b^ | 1.005 | 0.00^b^ | 0.153 |
|  | B | 24 | 86.89^b^ | 3.840 | 9.57^c^ | 1.813 | 3.39^b^ | 1.881 | 0.15^b^ | 1.005 | 0.00^b^ | 0.153 |
|  | C | 24 | 71.35^c^ | 3.840 | 11.62^c^ | 1.813 | 11.48^a^ | 1.881 | 5.36^a^ | 1.005 | 0.19^b^ | 0.153 |
| *p-value* | |  | *<0.0001* | | *<0.0001* | | *<0.0001* | | *<0.0001* | | *0.0036* | |

^1^Values are presented as least squares means (LSmeans). SE: Standard error. BD: Basal diet; PR: Basal diet + probiotic; PH: Basal diet + phytobiotic. F: female; M: male.

X and Y correspond to two fast growing commercial poultry genetic lines.

^a–d^Within a column, values without a common superscript differ, P < 0.05.

**Supplementary Table S8b:** Punctuation of footpad dermatitis (FPD) lesions (%) per day^1^.

|  |  |  |  | **Day 7** | | | | **Day 21** | | | | | | | | | | **Day 35** | | | | | | | | | |
| --- | --- | --- | --- | --- | --- | --- | --- | --- | --- | --- | --- | --- | --- | --- | --- | --- | --- | --- | --- | --- | --- | --- | --- | --- | --- | --- | --- |
| **Punctuation of FPD (%)** | | | **N** | **0** | | **1** | | **0** | | **1** | | **2** | | **3** | | **4** | | **0** | | **1** | | **2** | | **3** | | **4** | |
|  |  |  |  | **LS** | **SE** | **LS** | **SE** | **LS** | **SE** | **LS** | **SE** | **LS** | **SE** | **LS** | **SE** | **LS** | **SE** | **LS** | **SE** | **LS** | **SE** | **LS** | **SE** | **LS** | **SE** | **LS** | **SE** |
| **Treatment (TRT)** | | |  |  |  |  |  |  |  |  |  |  |  |  |  |  |  |  |  |  |  |  |  |  |  |  |  |
| BD | | | 24 | 99.79 | 0.094 | 0.21 | 0.094 | 81.61 | 3.824 | 10.12 | 1.827 | 6.65 | 2.249 | 1.38 | 0.587 | 0.24 | 0.232 | 68.46 | 4.612 | 17.28 | 1.856 | 11.70 | 2.040 | 2.56 | 1.637 | 0.00 | 0.110 |
| PR | | | 24 | 99.89 | 0.094 | 0.11 | 0.094 | 85.10 | 3.824 | 10.49 | 1.827 | 3.67 | 2.249 | 0.62 | 0.587 | 0.13 | 0.232 | 74.03 | 4.612 | 16.50 | 1.856 | 8.89 | 2.040 | 0.59 | 1.637 | 0.00 | 0.110 |
| PH | | | 24 | 100.00 | 0.094 | 0.00 | 0.094 | 88.52 | 3.824 | 7.25 | 1.827 | 2.81 | 2.249 | 1.02 | 0.587 | 0.39 | 0.232 | 74.59 | 4.612 | 15.18 | 1.856 | 7.68 | 2.040 | 2.37 | 1.637 | 0.19 | 0.110 |
| *P-value* | | |  | *0.2980* | | *0.2980* | | *0.2519* | | *0.2132* | | *0.2040* | | *0.5662* | | *0.6179* | | *0.3837* | | *0.7121* | | *0.2383* | | *0.5123* | | *0.3745* | |
| **Genetic line (GL)** | | |  |  |  |  |  |  |  |  |  |  |  |  |  |  |  |  |  |  |  |  |  |  |  |  |  |
| X | | | 36 | 99.86 | 0.076 | 0.14 | 0.076 | 81.75 | 3.437 | 11.13 | 1.637 | 5.19 | 2.058 | 1.42 | 0.512 | 0.51 | 0.204 | 63.48 | 4.164 | 19.29 | 1.529 | 13.57 | 1.789 | 3.54 | 1.448 | 0.13 | 0.089 |
| Y | | | 36 | 99.93 | 0.076 | 0.07 | 0.076 | 88.41 | 3.437 | 7.44 | 1.637 | 3.56 | 2.058 | 0.59 | 0.512 | 0.00 | 0.204 | 81.25 | 4.164 | 13.35 | 1.529 | 5.27 | 1.789 | 0.13 | 1.448 | 0.00 | 0.089 |
| *P-value* | | |  | *0.5334* | | *0.5334* | | *0.0523* | | *0.0270* | | *0.3714* | | *0.1574* | | *0.0253* | | *<0.0001* | | *0.0067* | | *<0.0001* | | *0.0295* | | *0.3217* | |
| **Sex** | | |  |  |  |  |  |  |  |  |  |  |  |  |  |  |  |  |  |  |  |  |  |  |  |  |  |
| F | | | 36 | 99.79 | 0.076 | 0.21 | 0.076 | 84.27 | 3.437 | 9.97 | 1.637 | 4.52 | 2.058 | 1.08 | 0.512 | 0.16 | 0.204 | 72.61 | 4.164 | 15.49 | 1.529 | 10.21 | 1.789 | 1.57 | 1.448 | 0.13 | 0.089 |
| M | | | 36 | 100.00 | 0.076 | 0.00 | 0.076 | 85.89 | 3.437 | 8.60 | 1.637 | 4.23 | 2.058 | 0.93 | 0.512 | 0.35 | 0.204 | 72.11 | 4.164 | 17.15 | 1.529 | 8.64 | 1.789 | 2.11 | 1.448 | 0.00 | 0.089 |
| *P-value* | | |  | *0.0573* | | *0.0573* | | *0.6307* | | *0.4032* | | *0.8742* | | *0.7934* | | *0.4071* | | *0.9000* | | *0.4351* | | *0.4269* | | *0.7235* | | *0.3217* | |
| **Experiment (EXP)** | | |  |  |  |  |  |  |  |  |  |  |  |  |  |  |  |  |  |  |  |  |  |  |  |  |  |
| A | | | 24 | 99.69^b^ | 0.094 | 0.32^a^ | 0.094 | 62.93^b^ | 5.195 | 20.16^a^ | 2.463 | 13.13^a^ | 3.198 | 3.02^a^ | 0.733 | 0.76 | 0.297 | 58.84^b^ | 6.343 | 27.77^a^ | 1.920 | 13.39^a^ | 2.590 | 0.00 | 2.132 | 0.00 | 0.110 |
| B | | | 24 | 100.00^a^ | 0.094 | 0.00^b^ | 0.094 | 95.34^a^ | 5.195 | 4.66^b^ | 2.463 | 0.00^b^ | 3.198 | 0.00^b^ | 0.733 | 0.00 | 0.297 | 86.89^a^ | 6.343 | 9.57^b^ | 1.920 | 3.39^b^ | 2.590 | 0.15 | 2.132 | 0.00 | 0.110 |
| C | | | 24 | 100.00^a^ | 0.094 | 0.00^b^ | 0.094 | 96.96^a^ | 5.195 | 3.04^b^ | 2.463 | 0.00^b^ | 3.198 | 0.00^b^ | 0.733 | 0.00 | 0.297 | 71.3^ab^ | 6.343 | 11.62^b^ | 1.920 | 11.48^a^ | 2.590 | 5.36 | 2.132 | 0.19 | 0.110 |
| *P-value* | | |  | *0.0292* | | *0.0292* | | *<0.0001* | | *<0.0001* | | *0.0060* | | *0.0059* | | *0.1227* | | *0.0110* | | *<0.0001* | | *0.0200* | | *0.1390* | | *0.3745* | |
| **TRT** | | **GL** |  |  |  |  |  |  |  |  |  |  |  |  |  |  |  |  |  |  |  |  |  |  |  |  |  |
| 1 | | X | 12 | 99.58 | 0.132 | 0.42 | 0.132 | 81.77 | 4.803 | 10.51 | 2.305 | 5.99 | 2.745 | 1.25 | 0.770 | 0.48 | 0.300 | 56.04 | 5.749 | 21.89 | 2.602 | 16.96 | 2.655 | 5.11 | 2.104 | 0.00 | 0.155 |
|  |  | Y | 12 | 100.00 | 0.132 | 0.00 | 0.132 | 81.45 | 4.803 | 9.74 | 2.305 | 7.31 | 2.745 | 1.50 | 0.770 | 0.00 | 0.300 | 80.88 | 5.749 | 12.68 | 2.602 | 6.44 | 2.655 | 0.00 | 2.104 | 0.00 | 0.155 |
| 2 | | X | 12 | 100.00 | 0.132 | 0.00 | 0.132 | 81.70 | 4.803 | 12.27 | 2.305 | 4.53 | 2.745 | 1.24 | 0.770 | 0.25 | 0.300 | 66.14 | 5.749 | 19.54 | 2.602 | 13.54 | 2.655 | 0.78 | 2.104 | 0.00 | 0.155 |
|  |  | Y | 12 | 99.79 | 0.132 | 0.21 | 0.132 | 88.49 | 4.803 | 8.70 | 2.305 | 2.81 | 2.745 | 0.00 | 0.770 | 0.00 | 0.300 | 81.93 | 5.749 | 13.45 | 2.602 | 4.23 | 2.655 | 0.40 | 2.104 | 0.00 | 0.155 |
| 3 | | X | 12 | 100.00 | 0.132 | 0.00 | 0.132 | 81.78 | 4.803 | 10.61 | 2.305 | 5.07 | 2.745 | 1.76 | 0.770 | 0.78 | 0.300 | 68.24 | 5.749 | 16.44 | 2.602 | 10.20 | 2.655 | 4.74 | 2.104 | 0.38 | 0.155 |
|  |  | Y | 12 | 100.00 | 0.132 | 0.00 | 0.132 | 95.27 | 4.803 | 3.89 | 2.305 | 0.56 | 2.745 | 0.28 | 0.770 | 0.00 | 0.300 | 80.93 | 5.749 | 13.92 | 2.602 | 5.15 | 2.655 | 0.00 | 2.104 | 0.00 | 0.155 |
| *P-value* | | |  | *0.0620* | | *0.0620* | | *0.2520* | | *0.3329* | | *0.4291* | | *0.4164* | | *0.6179* | | *0.4346* | | *0.4367* | | *0.4936* | | *0.3777* | | *0.3745* | |
| **TRT** | | **Sex** |  |  |  |  |  |  |  |  |  |  |  |  |  |  |  |  |  |  |  |  |  |  |  |  |  |
| 1 | | F | 12 | 99.58 | 0.132 | 0.42 | 0.132 | 78.75 | 4.803 | 11.33 | 2.305 | 7.95 | 2.745 | 1.74 | 0.770 | 0.24 | 0.300 | 67.73 | 5.749 | 17.82 | 2.602 | 13.62 | 2.655 | 0.84 | 2.104 | 0.00 | 0.155 |
|  |  | M | 12 | 100.00 | 0.132 | 0.00 | 0.132 | 84.48 | 4.803 | 8.92 | 2.305 | 5.34 | 2.745 | 1.01 | 0.770 | 0.25 | 0.300 | 69.20 | 5.749 | 16.75 | 2.602 | 9.78 | 2.655 | 4.28 | 2.104 | 0.00 | 0.155 |
| 2 | | F | 12 | 99.79 | 0.132 | 0.21 | 0.132 | 88.41 | 4.803 | 9.24 | 2.305 | 2.36 | 2.745 | 0.00 | 0.770 | 0.00 | 0.300 | 75.51 | 5.749 | 16.42 | 2.602 | 8.07 | 2.655 | 0.00 | 2.104 | 0.00 | 0.155 |
|  |  | M | 12 | 100.00 | 0.132 | 0.00 | 0.132 | 81.79 | 4.803 | 11.73 | 2.305 | 4.98 | 2.745 | 1.24 | 0.770 | 0.25 | 0.300 | 72.56 | 5.749 | 16.57 | 2.602 | 9.70 | 2.655 | 1.17 | 2.104 | 0.00 | 0.155 |
| 3 | | F | 12 | 100.00 | 0.132 | 0.00 | 0.132 | 85.65 | 4.803 | 9.35 | 2.305 | 3.25 | 2.745 | 1.50 | 0.770 | 0.25 | 0.300 | 74.60 | 5.749 | 12.23 | 2.602 | 8.93 | 2.655 | 3.86 | 2.104 | 0.38 | 0.155 |
|  |  | M | 12 | 100.00 | 0.132 | 0.00 | 0.132 | 91.40 | 4.803 | 5.15 | 2.305 | 2.37 | 2.745 | 0.54 | 0.770 | 0.54 | 0.300 | 74.57 | 5.749 | 18.12 | 2.602 | 6.43 | 2.655 | 0.88 | 2.104 | 0.00 | 0.155 |
| *P-value* | | |  | *0.2980* | | *0.2980* | | *0.2305* | | *0.2281* | | *0.4912* | | *0.2374* | | *0.8485* | | *0.8987* | | *0.3608* | | *0.4990* | | *0.2276* | | *0.3745* | |
| **GL** | | **Sex** |  |  |  |  |  |  |  |  |  |  |  |  |  |  |  |  |  |  |  |  |  |  |  |  |  |
| X | | F | 18 | 99.72 | 0.108 | 0.28 | 0.108 | 81.19 | 4.176 | 12.19 | 1.999 | 4.98 | 2.426 | 1.32 | 0.654 | 0.32 | 0.256 | 63.32 | 5.019 | 17.92 | 2.134 | 15.38 | 2.264 | 3.13 | 1.806 | 0.25 | 0.126 |
|  |  | M | 18 | 100.00 | 0.108 | 0.00 | 0.108 | 82.31 | 4.176 | 10.07 | 1.999 | 5.41 | 2.426 | 1.51 | 0.654 | 0.69 | 0.256 | 63.63 | 5.019 | 20.66 | 2.134 | 11.76 | 2.264 | 3.95 | 1.806 | 0.00 | 0.126 |
| Y | | F | 18 | 99.86 | 0.108 | 0.14 | 0.108 | 87.35 | 4.176 | 7.75 | 1.999 | 4.06 | 2.426 | 0.84 | 0.654 | 0.00 | 0.256 | 81.90 | 5.019 | 13.06 | 2.134 | 5.04 | 2.264 | 0.00 | 1.806 | 0.00 | 0.126 |
|  |  | M | 18 | 100.00 | 0.108 | 0.00 | 0.108 | 89.47 | 4.176 | 7.13 | 1.999 | 3.05 | 2.426 | 0.35 | 0.654 | 0.00 | 0.256 | 80.59 | 5.019 | 13.64 | 2.134 | 5.51 | 2.264 | 0.26 | 1.806 | 0.00 | 0.126 |
| *P-value* | | |  | *0.5334* | | *0.5334* | | *0.8836* | | *0.6464* | | *0.6941* | | *0.5573* | | *0.4071* | | *0.8390* | | *0.6085* | | *0.3023* | | *0.8560* | | *0.3217* | |
| **GL** | **Sex** | **TRT** |  |  |  |  |  |  |  |  |  |  |  |  |  |  |  |  |  |  |  |  |  |  |  |  |  |
| X | F | 1 | 6 | 99.17 | 0.187 | 0.83 | 0.187 | 77.82 | 6.322 | 13.16 | 3.044 | 7.02 | 3.534 | 1.52 | 1.044 | 0.48 | 0.403 | 59.44 | 7.524 | 21.05 | 3.663 | 17.84 | 3.582 | 1.67 | 2.815 | 0.00 | 0.219 |
|  |  | 2 | 6 | 100.00 | 0.187 | 0.00 | 0.187 | 85.72 | 6.322 | 7.86 | 3.044 | 4.95 | 3.534 | 0.98 | 1.044 | 0.49 | 0.403 | 52.65 | 7.524 | 22.72 | 3.663 | 16.08 | 3.582 | 8.55 | 2.815 | 0.00 | 0.219 |
|  |  | 3 | 6 | 100.00 | 0.187 | 0.00 | 0.187 | 79.67 | 6.322 | 9.49 | 3.044 | 8.89 | 3.534 | 1.96 | 1.044 | 0.00 | 0.403 | 76.02 | 7.524 | 14.58 | 3.663 | 9.40 | 3.582 | 0.00 | 2.815 | 0.00 | 0.219 |
|  | M | 1 | 6 | 100.00 | 0.187 | 0.00 | 0.187 | 83.24 | 6.322 | 9.99 | 3.044 | 5.73 | 3.534 | 1.04 | 1.044 | 0.00 | 0.403 | 85.74 | 7.524 | 10.79 | 3.663 | 3.47 | 3.582 | 0.00 | 2.815 | 0.00 | 0.219 |
|  |  | 2 | 6 | 100.00 | 0.187 | 0.00 | 0.187 | 87.58 | 6.322 | 9.90 | 3.044 | 2.53 | 3.534 | 0.00 | 1.044 | 0.00 | 0.403 | 65.31 | 7.524 | 20.64 | 3.663 | 14.05 | 3.582 | 0.00 | 2.815 | 0.00 | 0.219 |
|  |  | 3 | 6 | 100.00 | 0.187 | 0.00 | 0.187 | 75.83 | 6.322 | 14.65 | 3.044 | 6.54 | 3.534 | 2.48 | 1.044 | 0.51 | 0.403 | 66.97 | 7.524 | 18.45 | 3.663 | 13.04 | 3.582 | 1.55 | 2.815 | 0.00 | 0.219 |
| Y | F | 1 | 6 | 99.57 | 0.187 | 0.43 | 0.187 | 89.24 | 6.322 | 8.58 | 3.044 | 2.19 | 3.534 | 0.00 | 1.044 | 0.00 | 0.403 | 85.70 | 7.524 | 12.21 | 3.663 | 2.09 | 3.582 | 0.00 | 2.815 | 0.00 | 0.219 |
|  |  | 2 | 6 | 100.00 | 0.187 | 0.00 | 0.187 | 87.75 | 6.322 | 8.82 | 3.044 | 3.43 | 3.534 | 0.00 | 1.044 | 0.00 | 0.403 | 78.15 | 7.524 | 14.69 | 3.663 | 6.37 | 3.582 | 0.79 | 2.815 | 0.00 | 0.219 |
|  |  | 3 | 6 | 100.00 | 0.187 | 0.00 | 0.187 | 78.16 | 6.322 | 13.51 | 3.044 | 5.39 | 3.534 | 2.45 | 1.044 | 0.49 | 0.403 | 65.22 | 7.524 | 12.06 | 3.663 | 14.24 | 3.582 | 7.73 | 2.815 | 0.76 | 0.219 |
|  | M | 1 | 6 | 100.00 | 0.187 | 0.00 | 0.187 | 85.39 | 6.322 | 7.72 | 3.044 | 4.74 | 3.534 | 1.08 | 1.044 | 1.08 | 0.403 | 71.27 | 7.524 | 20.81 | 3.663 | 6.17 | 3.582 | 1.76 | 2.815 | 0.00 | 0.219 |
|  |  | 2 | 6 | 100.00 | 0.187 | 0.00 | 0.187 | 93.14 | 6.322 | 5.19 | 3.044 | 1.11 | 3.534 | 0.56 | 1.044 | 0.00 | 0.403 | 83.98 | 7.524 | 12.40 | 3.663 | 3.62 | 3.582 | 0.00 | 2.815 | 0.00 | 0.219 |
|  |  | 3 | 6 | 100.00 | 0.187 | 0.00 | 0.187 | 97.41 | 6.322 | 2.59 | 3.044 | 0.00 | 3.534 | 0.00 | 1.044 | 0.00 | 0.403 | 77.88 | 7.524 | 15.43 | 3.663 | 6.69 | 3.582 | 0.00 | 2.815 | 0.00 | 0.219 |
| *P-value* | | |  | *0.0620* | | *0.0620* | | *0.6213* | | *0.4094* | | *0.9648* | | *0.4995* | | *0.8485* | | *0.2754* | | *0.5204* | | *0.2823* | | *0.2368* | | *0.3745* | |

^1^Values presented as least squares means (LS). SE: Standard error. BD: Basal diet; PR: BD + probiotic; PH: BD + phytobiotic. F: female; M: male. ^a–d^Within a column, values without a common superscript differ, P<0.05. X and Y: two fast growing commercial poultry genetic lines.

**Supplementary Table S9:** Litter quality (pH)^1^.

|  | | | **N** | **Litter pH 0-35 days** | | **N** | **Litter pH day 7** | | **Litter pH day 21** | | **Litter pH day 35** | |
| --- | --- | --- | --- | --- | --- | --- | --- | --- | --- | --- | --- | --- |
|  |  |  |  | **LSmeans** | **SE** |  | **LSmeans** | **SE** | **LSmeans** | **SE** | **LSmeans** | **SE** |
| **Treatment (TRT)** | | |  |  |  |  |  |  |  |  |  |  |
| BD | | | 72 | 7.27 | 0.086 | 24 | 6.35 | 0.188 | 7.53 | 0.128 | 7.93 | 0.130 |
| PR | | | 72 | 7.15 | 0.086 | 24 | 6.17 | 0.188 | 7.55 | 0.128 | 7.72 | 0.130 |
| PH | | | 72 | 7.22 | 0.086 | 24 | 6.43 | 0.188 | 7.45 | 0.128 | 7.79 | 0.130 |
| *p-value* | | |  | *0.6039* | |  | *0.5461* | | *0.8440* | | *0.4271* | |
| **Genetic line (GL)** | | |  |  |  |  |  |  |  |  |  |  |
| X | | | 108 | 7.13 | 0.070 | 36 | 6.11 | 0.160 | 7.44 | 0.104 | 7.83 | 0.112 |
| Y | | | 108 | 7.30 | 0.070 | 36 | 6.52 | 0.160 | 7.57 | 0.104 | 7.79 | 0.112 |
| p-value | | |  | *0.0896* | |  | *0.0427* | | *0.3813* | | *0.8098* | |
| **Sex** | | |  |  |  |  |  |  |  |  |  |  |
| F | | | 108 | 7.18 | 0.070 | 36 | 6.32 | 0.160 | 7.44 | 0.104 | 7.78 | 0.112 |
| M | | | 108 | 7.24 | 0.070 | 36 | 6.31 | 0.160 | 7.58 | 0.104 | 7.84 | 0.112 |
| p-value | | |  | *0.5088* | |  | *0.9823* | | *0.3479* | | *0.6394* | |
| **Experiment (EXP)** | | |  |  |  |  |  |  |  |  |  |  |
| A | | | 72 | 6.98^b^ | 0.086 | 24 | 6.27 | 0.216 | 7.38 | 0.128 | 7.29^c^ | 0.156 |
| B | | | 72 | 7.34^a^ | 0.086 | 24 | 6.69 | 0.216 | 7.55 | 0.128 | 7.79^b^ | 0.156 |
| C | | | 72 | 7.31^a^ | 0.086 | 24 | 5.98 | 0.216 | 7.59 | 0.128 | 8.35^a^ | 0.156 |
| *p-value* | | |  | *0.0059* | |  | *0.0736* | | *0.4607* | | *<0.0001* | |
| **TRT** | | **GL** |  |  |  |  |  |  |  |  |  |  |
| BD | | X | 36 | 7.21 | 0.122 | 12 | 6.29 | 0.255 | 7.37 | 0.180 | 7.98 | 0.174 |
|  |  | Y | 36 | 7.32 | 0.122 | 12 | 6.40 | 0.255 | 7.68 | 0.180 | 7.88 | 0.174 |
| PR | | X | 36 | 7.00 | 0.122 | 12 | 5.97 | 0.255 | 7.47 | 0.180 | 7.58 | 0.174 |
|  |  | Y | 36 | 7.29 | 0.122 | 12 | 6.37 | 0.255 | 7.63 | 0.180 | 7.86 | 0.174 |
| PH | | X | 36 | 7.17 | 0.122 | 12 | 6.08 | 0.255 | 7.50 | 0.180 | 7.93 | 0.174 |
|  |  | Y | 36 | 7.28 | 0.122 | 12 | 6.79 | 0.255 | 7.40 | 0.180 | 7.65 | 0.174 |
| *P-value* | | |  | *0.7200* | |  | *0.4685* | | *0.5246* | | *0.2298* | |
| **TRT** | | **Sex** |  |  |  |  |  |  |  |  |  |  |
| BD | | F | 36 | 7.33 | 0.122 | 12 | 6.34 | 0.255 | 7.72 | 0.180 | 7.95 | 0.174 |
|  |  | M | 36 | 7.20 | 0.122 | 12 | 6.36 | 0.255 | 7.33 | 0.180 | 7.91 | 0.174 |
| PR | | F | 36 | 7.10 | 0.122 | 12 | 6.28 | 0.255 | 7.38 | 0.180 | 7.63 | 0.174 |
|  |  | M | 36 | 7.20 | 0.122 | 12 | 6.06 | 0.255 | 7.73 | 0.180 | 7.80 | 0.174 |
| PH | | F | 36 | 7.11 | 0.122 | 12 | 6.34 | 0.255 | 7.22 | 0.180 | 7.76 | 0.174 |
|  |  | M | 36 | 7.34 | 0.122 | 12 | 6.53 | 0.255 | 7.68 | 0.180 | 7.82 | 0.174 |
| *P-value* | | |  | *0.3093* | |  | *0.6976* | | *0.0468** | | *0.8095* | |
| **GL** | | **Sex** |  |  |  |  |  |  |  |  |  |  |
| X | | F | 54 | 7.12 | 0.099 | 18 | 6.11 | 0.213 | 7.43 | 0.147 | 7.83 | 0.146 |
|  |  | M | 54 | 7.13 | 0.099 | 18 | 6.11 | 0.213 | 7.46 | 0.147 | 7.83 | 0.146 |
| Y | | F | 54 | 7.24 | 0.099 | 18 | 6.53 | 0.213 | 7.45 | 0.147 | 7.73 | 0.146 |
|  |  | M | 54 | 7.36 | 0.099 | 18 | 6.52 | 0.213 | 7.70 | 0.147 | 7.86 | 0.146 |
| *P-value* | | |  | *0.5725* | |  | *0.9624* | | *0.4383* | | *0.6364* | |
| **GL** | **Sex** | **TRT** |  |  |  |  |  |  |  |  |  |  |
| X | F | BD | 18 | 7.40 | 0.172 | 6 | 6.26 | 0.353 | 7.91^a^ | 0.255 | 8.03 | 0.239 |
|  |  | PR | 18 | 6.98 | 0.172 | 6 | 6.32 | 0.353 | 6.82^c^ | 0.255 | 7.93 | 0.239 |
|  |  | PH | 18 | 6.99 | 0.172 | 6 | 6.41 | 0.353 | 7.53^abc^ | 0.255 | 7.87 | 0.239 |
|  | M | BD | 18 | 7.02 | 0.172 | 6 | 6.39 | 0.353 | 7.84^a^ | 0.255 | 7.89 | 0.239 |
|  |  | PR | 18 | 7.03 | 0.172 | 6 | 6.16 | 0.353 | 7.30^abc^ | 0.255 | 7.49 | 0.239 |
|  |  | PH | 18 | 7.35 | 0.172 | 6 | 5.77 | 0.353 | 7.64^ab^ | 0.255 | 7.67 | 0.239 |
| Y | F | BD | 18 | 7.27 | 0.172 | 6 | 6.40 | 0.353 | 7.45^abc^ | 0.255 | 7.78 | 0.239 |
|  |  | PR | 18 | 7.21 | 0.172 | 6 | 6.35 | 0.353 | 7.82^a^ | 0.255 | 7.94 | 0.239 |
|  |  | PH | 18 | 7.23 | 0.172 | 6 | 5.90 | 0.353 | 7.09^bc^ | 0.255 | 7.97 | 0.239 |
|  | M | BD | 18 | 7.37 | 0.172 | 6 | 6.25 | 0.353 | 7.91^a^ | 0.255 | 7.89 | 0.239 |
|  |  | PR | 18 | 7.37 | 0.172 | 6 | 6.78 | 0.353 | 7.36^abc^ | 0.255 | 7.55 | 0.239 |
|  |  | PH | 18 | 7.34 | 0.172 | 6 | 6.81 | 0.353 | 7.45^abc^ | 0.255 | 7.75 | 0.239 |
| *P-value* | | |  | *0.3237* | |  | *0.7948* | | *0.0149* | | *0.8954* | |
| **Day** | | |  |  |  |  |  |  |  |  |  |  |
| 7 | | | 72 | 6.32^c^ | 0.09 |  |  |  |  |  |  |  |
| 21 | | | 72 | 7.51^b^ | 0.09 |  |  |  |  |  |  |  |
| 35 | | | 72 | 7.81^a^ | 0.09 |  |  |  |  |  |  |  |
| *P-value* | | |  | *<0.0001* | |  |  |  |  |  |  |  |
| **TRT** | | **Day** |  |  |  |  |  |  |  |  |  |  |
| BD | | 7 | 24 | 6.35 | 0.149 |  |  |  |  |  |  |  |
|  |  | 21 | 24 | 7.53 | 0.149 |  |  |  |  |  |  |  |
|  |  | 35 | 24 | 7.93 | 0.149 |  |  |  |  |  |  |  |
| PR | | 7 | 24 | 6.17 | 0.149 |  |  |  |  |  |  |  |
|  |  | 21 | 24 | 7.55 | 0.149 |  |  |  |  |  |  |  |
|  |  | 35 | 24 | 7.72 | 0.149 |  |  |  |  |  |  |  |
| PH | | 7 | 24 | 6.43 | 0.149 |  |  |  |  |  |  |  |
|  |  | 21 | 24 | 7.45 | 0.149 |  |  |  |  |  |  |  |
|  |  | 35 | 24 | 7.79 | 0.149 |  |  |  |  |  |  |  |
| *P-value* | | |  | *0.7528* | |  |  |  |  |  |  |  |
| **GL** | | **Day** |  |  |  |  |  |  |  |  |  |  |
| X | | 7 | 36 | 6.11 | 0.122 |  |  |  |  |  |  |  |
|  |  | 21 | 36 | 7.44 | 0.122 |  |  |  |  |  |  |  |
|  |  | 35 | 36 | 7.83 | 0.122 |  |  |  |  |  |  |  |
| Y | | 7 | 36 | 6.52 | 0.122 |  |  |  |  |  |  |  |
|  |  | 21 | 36 | 7.57 | 0.122 |  |  |  |  |  |  |  |
|  |  | 35 | 36 | 7.79 | 0.122 |  |  |  |  |  |  |  |
| *P-value* | | |  | *0.1851* | |  |  |  |  |  |  |  |
| **Sex** | | **Day** |  |  |  |  |  |  |  |  |  |  |
| F | | 7 | 36 | 6.32 | 0.122 |  |  |  |  |  |  |  |
|  |  | 21 | 36 | 7.44 | 0.122 |  |  |  |  |  |  |  |
|  |  | 35 | 36 | 7.78 | 0.122 |  |  |  |  |  |  |  |
| M | | 7 | 36 | 6.31 | 0.122 |  |  |  |  |  |  |  |
|  |  | 21 | 36 | 7.58 | 0.122 |  |  |  |  |  |  |  |
|  |  | 35 | 36 | 7.84 | 0.122 |  |  |  |  |  |  |  |
| *P-value* | | |  | *0.8399* | |  |  |  |  |  |  |  |

^1^Values are presented as least squares means (LSmeans). SE: Standard error. BD: Basal diet; PR: Basal diet + probiotic; PH: Basal diet + phytobiotic. F: female; M: male.

X and Y correspond to two fast growing commercial poultry genetic lines.

^a–d^Within a column, values without a common superscript differ, P < 0.05.

*Analysis of variance reached significance, but the mean comparisons did not reach statistical significance between groups.

**Supplementary Table S10:** Meat oxidative stability.

|  | | | **N** | **TBARS** | |
| --- | --- | --- | --- | --- | --- |
|  |  |  |  | **LSmeans** | **SE** |
| **Treatment (TRT)** | | |  |  |  |
| BD | | | 24 | 0.06 | 0.004 |
| PR | | | 24 | 0.06 | 0.004 |
| PH | | | 24 | 0.06 | 0.004 |
| *P-value* | | |  | *0.9670* | |
| **Genetic line (GL)** | | |  |  |  |
| X | | | 36 | 0.06 | 0.004 |
| Y | | | 36 | 0.07 | 0.004 |
| *P-value* | | |  | *0.0538* | |
| **Sex** | | |  |  |  |
| F | | | 36 | 0.07 | 0.004 |
| M | | | 36 | 0.06 | 0.004 |
| *P-value* | | |  | *0.3732* | |
| **Experiment (EXP)** | | |  |  |  |
| A | | | 24 | 0.05^b^ | 0.006 |
| B | | | 24 | 0.10^a^ | 0.006 |
| C | | | 24 | 0.05^b^ | 0.006 |
| *P-value* | | |  | *<0.0001* | |
| **TRT** | | **GL** |  |  |  |
| BD | | X | 12 | 0.06 | 0.006 |
|  |  | Y | 12 | 0.07 | 0.006 |
| PR | | X | 12 | 0.06 | 0.006 |
|  |  | Y | 12 | 0.07 | 0.006 |
| PH | | X | 12 | 0.06 | 0.006 |
|  |  | Y | 12 | 0.07 | 0.006 |
| *P-value* | | |  | *0.3879* | |
| **TRT** | | **Sex** |  |  |  |
| BD | | F | 12 | 0.06 | 0.006 |
|  |  | M | 12 | 0.06 | 0.006 |
| PR | | F | 12 | 0.07 | 0.006 |
|  |  | M | 12 | 0.06 | 0.006 |
| PH | | F | 12 | 0.07 | 0.006 |
|  |  | M | 12 | 0.06 | 0.006 |
| *P-value* | | |  | *0.7154* | |
| **GL** | | **Sex** |  |  |  |
| X | | F | 18 | 0.06 | 0.005 |
|  |  | M | 18 | 0.06 | 0.005 |
| Y | | F | 18 | 0.07 | 0.005 |
|  |  | M | 18 | 0.07 | 0.005 |
| *P-value* | | |  | *0.2349* | |
| **GL** | **Sex** | **TRT** |  |  |  |
| X | F | BD | 6 | 0.05 | 0.007 |
|  |  | PR | 6 | 0.06 | 0.007 |
|  |  | PH | 6 | 0.07 | 0.007 |
|  | M | BD | 6 | 0.07 | 0.007 |
|  |  | PR | 6 | 0.07 | 0.007 |
|  |  | PH | 6 | 0.06 | 0.007 |
| Y | F | BD | 6 | 0.07 | 0.007 |
|  |  | PR | 6 | 0.07 | 0.007 |
|  |  | PH | 6 | 0.07 | 0.007 |
|  | M | BD | 6 | 0.05 | 0.007 |
|  |  | PR | 6 | 0.06 | 0.007 |
|  |  | PH | 6 | 0.07 | 0.007 |
| *P-value* | | |  | *0.3027* | |

^1^Values are presented as least squares means (LSmeans). SE: Standard error.

TBARS: Thiobarbituric acid reactive substances (TBARS units). BD: Basal diet; PR: Basal diet + probiotic; PH: Basal diet + phytobiotic. F: female; M: male.

X and Y correspond to two fast growing commercial poultry genetic lines.

^a–d^Within a column, values without a common superscript differ, P < 0.05.

**Supplementary Table S11:** Carcass cuts (%)^1^.

|  | | | **N** | **Carcass yield %** | | **Breast yield %** | | **Thigh yield %** | | **Abdominal Fat yield %** | |
| --- | --- | --- | --- | --- | --- | --- | --- | --- | --- | --- | --- |
|  |  |  |  | **LSmeans** | **SE** | **LSmeans** | **SE** | **LSmeans** | **SE** | **LSmeans** | **SE** |
| **Treatment (TRT)** | | |  |  |  |  |  |  |  |  |  |
| BD | | | 24 | 76.6 | 0.24 | 19.0 | 0.12 | 20.4 | 0.09 | 1.5 | 0.03 |
| PR | | | 24 | 76.4 | 0.24 | 18.9 | 0.12 | 20.5 | 0.09 | 1.5 | 0.03 |
| PH | | | 24 | 76.3 | 0.24 | 18.8 | 0.12 | 20.4 | 0.09 | 1.4 | 0.03 |
| *P-value* | | |  | *0.7134* | | *0.5932* | | *0.5923* | | *0.5720* | |
| **Genetic line (GL)** | | |  |  |  |  |  |  |  |  |  |
| X | | | 36 | 76.7 | 0.24 | 19.6^a^ | 0.12 | 20.2^b^ | 0.09 | 1.5^a^ | 0.03 |
| Y | | | 36 | 76.2 | 0.24 | 18.2^b^ | 0.12 | 20.7^a^ | 0.09 | 1.4^b^ | 0.03 |
| *P-value* | | |  | *0.1676* | | *<0.0001* | | *0.0004* | | *0.0066* | |
| **Sex** | | |  |  |  |  |  |  |  |  |  |
| F | | | 36 | 76.7 | 0.23 | 19.6^a^ | 0.12 | 20.0^b^ | 0.08 | 1.6^a^ | 0.03 |
| M | | | 36 | 76.2 | 0.23 | 18.2^b^ | 0.12 | 20.8^a^ | 0.08 | 1.3^b^ | 0.03 |
| *P-value* | | |  | *0.1534* | | *<0.0001* | | *<0.0001* | | *<0.0001* | |
| **Experiment (EXP)** | | |  |  |  |  |  |  |  |  |  |
| A | | | 36 | 76.7 | 0.29 | 19.6^a^ | 0.15 | 20.2^b^ | 0.11 | 1.5 | 0.04 |
| B | | | 36 | 76.1 | 0.28 | 19.5^a^ | 0.14 | 20.1^b^ | 0.11 | 1.4 | 0.04 |
| C | | |  | 76.6 | 0.39 | 17.7^b^ | 0.20 | 21.0^a^ | 0.14 | 1.4 | 0.05 |
| *P-value* | | |  | *0.2266* | | *<0.0001* | | *0.0001* | | *0.3580* | |
| **TRT** | | **GL** |  |  |  |  |  |  |  |  |  |
| BD | | X | 12 | 77.1 | 0.35 | 19.9^a^ | 0.18 | 20.1 | 0.12 | 1.5 | 0.05 |
|  |  | Y | 12 | 76.1 | 0.35 | 18.1^d^ | 0.18 | 20.8 | 0.12 | 1.4 | 0.05 |
| PR | | X | 12 | 76.5 | 0.39 | 19.3^bc^ | 0.20 | 20.3 | 0.13 | 1.5 | 0.05 |
|  |  | Y | 12 | 76.4 | 0.36 | 18.5^dc^ | 0.19 | 20.6 | 0.12 | 1.4 | 0.05 |
| PH | | X | 12 | 76.5 | 0.36 | 19.6^ab^ | 0.19 | 20.1 | 0.12 | 1.5 | 0.05 |
|  |  | Y | 12 | 76.1 | 0.38 | 18.1^d^ | 0.20 | 20.6 | 0.13 | 1.3 | 0.05 |
| *P-value* | | |  | *0.3510* | | *0.0218* | | *0.1784* | | *0.7382* | |
| **TRT** | | **Sex** |  |  |  |  |  |  |  |  |  |
| BD | | F | 12 | 76.7 | 0.36 | 19.5 | 0.19 | 20.0 | 0.12 | 1.6 | 0.05 |
|  |  | M | 12 | 76.5 | 0.36 | 18.5 | 0.19 | 20.8 | 0.12 | 1.3 | 0.05 |
| PR | | F | 12 | 76.8 | 0.35 | 19.7 | 0.18 | 20.0 | 0.12 | 1.6 | 0.05 |
|  |  | M | 12 | 76.0 | 0.37 | 18.1 | 0.19 | 21.0 | 0.13 | 1.3 | 0.05 |
| PH | | F | 12 | 76.6 | 0.36 | 19.5 | 0.19 | 20.0 | 0.13 | 1.6 | 0.05 |
|  |  | M | 12 | 76.0 | 0.35 | 18.2 | 0.18 | 20.7 | 0.12 | 1.2 | 0.05 |
| *P-value* | | |  | *0.6956* | | *0.2003* | | *0.5371* | | *0.2347* | |
| **GL** | | **Sex** |  |  |  |  |  |  |  |  |  |
| X | | F | 18 | 77.0 | 0.28 | 20.1 | 0.14 | 19.9^c^ | 0.10 | 1.6 | 0.04 |
|  |  | M | 18 | 76.4 | 0.41 | 19.0 | 0.22 | 20.5^b^ | 0.14 | 1.4 | 0.06 |
| Y | | F | 18 | 76.4 | 0.35 | 19.0 | 0.18 | 20.1^cb^ | 0.12 | 1.5 | 0.05 |
|  |  | M | 18 | 76.0 | 0.28 | 17.5 | 0.15 | 21.2^a^ | 0.10 | 1.2 | 0.04 |
| *P-value* | | |  | *0.7640* | | *0.1776* | | *0.0318* | | *0.5458* | |
| **GL** | **Sex** | **TRT** |  |  |  |  |  |  |  |  |  |
| X | F | BD | 6 | 77.2 | 0.47 | 20.2^ab^ | 0.25 | 19.8 | 0.16 | 1.7 | 0.06 |
|  |  | PR | 6 | 76.7 | 0.47 | 19.8^ab^ | 0.25 | 19.8 | 0.16 | 1.6 | 0.06 |
|  |  | PH | 6 | 77.2 | 0.47 | 20.5^a^ | 0.25 | 20.0 | 0.16 | 1.7 | 0.06 |
|  | M | BD | 6 | 77.1 | 0.53 | 19.6^bc^ | 0.28 | 20.4 | 0.18 | 1.4 | 0.07 |
|  |  | PR | 6 | 76.3 | 0.62 | 18.7^de^ | 0.33 | 20.8 | 0.21 | 1.4 | 0.09 |
|  |  | PH | 6 | 75.9 | 0.55 | 18.7^de^ | 0.29 | 20.3 | 0.18 | 1.3 | 0.07 |
| Y | F | BD | 6 | 76.2 | 0.52 | 18.9^cde^ | 0.27 | 20.3 | 0.18 | 1.5 | 0.07 |
|  |  | PR | 6 | 77.0 | 0.51 | 19.6^bcd^ | 0.27 | 20.1 | 0.17 | 1.6 | 0.07 |
|  |  | PH | 6 | 76.0 | 0.54 | 18.6^e^ | 0.28 | 20.0 | 0.18 | 1.5 | 0.07 |
|  | M | BD | 6 | 75.9 | 0.47 | 17.4^f^ | 0.25 | 21.3 | 0.16 | 1.3 | 0.06 |
|  |  | PR | 6 | 75.8 | 0.48 | 17.4^f^ | 0.25 | 21.1 | 0.16 | 1.3 | 0.07 |
|  |  | PH | 6 | 76.2 | 0.48 | 17.6^f^ | 0.25 | 21.2 | 0.16 | 1.1 | 0.07 |
| *P-value* | | |  | *0.1869* | | *0.0181* | | *0.1813* | | *0.5572* | |

^1^Values are presented as least squares means (LSmeans). SE: Standard error. BD: Basal diet; PR: Basal diet + probiotic; PH: Basal diet + phytobiotic. F: female; M: male.

X and Y correspond to two fast growing commercial poultry genetic lines.

^a–d^Within a column, values without a common superscript differ, P < 0.05.

**Supplementary Table S12:** Variability of individual body weight of sampled animals.

|  | | | | **N** | **Individual BW** | | |
| --- | --- | --- | --- | --- | --- | --- | --- |
|  |  |  |  |  | **Mean** | **SD** | **CV** |
| **Genetic line** | | | **Day** | 216 | 194 | 25.6 | 13.2 |
| X | | | 7 |  |  |  |  |
|  |  |  | 21 | 216 | 995 | 110.7 | 11.1 |
|  |  |  | 35 | 216 | 2280 | 302.6 | 13.3 |
| Y | | | 7 | 216 | 189 | 21.5 | 11.4 |
|  |  |  | 21 | 216 | 891 | 119.2 | 13.4 |
|  |  |  | 35 | 216 | 2029 | 322.4 | 15.9 |
| **Experiment** | | **Genetic line** | **Day** | 72 | 194 | 27.4 | 14.2 |
| A | | X | 7 |  |  |  |  |
|  |  |  | 21 | 72 | 996 | 111.2 | 11.2 |
|  |  |  | 35 | 72 | 2403 | 268.1 | 11.2 |
|  |  | Y | 7 | 72 | 189 | 22.7 | 12.0 |
|  |  |  | 21 | 72 | 892 | 124.9 | 14.0 |
|  |  |  | 35 | 72 | 2142 | 228.0 | 10.7 |
| B | | X | 7 | 72 | 201 | 24.9 | 12.4 |
|  |  |  | 21 | 72 | 1004 | 115.1 | 11.5 |
|  |  |  | 35 | 72 | 2361 | 285.6 | 12.1 |
|  |  | Y | 7 | 72 | 195 | 20.7 | 10.6 |
|  |  |  | 21 | 72 | 942 | 94.2 | 10.0 |
|  |  |  | 35 | 72 | 2183 | 280.5 | 12.9 |
| C | | X | 7 | 72 | 189 | 23.3 | 12.3 |
|  |  |  | 21 | 72 | 985 | 106.2 | 10.8 |
|  |  |  | 35 | 72 | 2077 | 245.1 | 11.8 |
|  |  | Y | 7 | 72 | 182 | 19.5 | 10.7 |
|  |  |  | 21 | 72 | 841 | 115.6 | 13.7 |
|  |  |  | 35 | 72 | 1762 | 274.0 | 15.6 |
| **Experiment** | **Pen** | **Genetic line** | **Day** | 6 | 190 | 32.1 | 16.9 |
| A | 1 | X | 7 |  |  |  |  |
|  |  |  | 21 | 6 | 1020 | 72.6 | 7.1 |
|  |  |  | 35 | 6 | 2660 | 186.4 | 7.0 |
|  | 2 | Y | 7 | 6 | 193 | 41.2 | 21.3 |
|  |  |  | 21 | 6 | 848 | 116.0 | 13.7 |
|  |  |  | 35 | 6 | 2238 | 148.3 | 6.6 |
|  | 3 | X | 7 | 6 | 196 | 31.3 | 16.0 |
|  |  |  | 21 | 6 | 994 | 66.0 | 6.6 |
|  |  |  | 35 | 6 | 2227 | 250.1 | 11.2 |
|  | 4 | X | 7 | 6 | 190 | 20.2 | 10.6 |
|  |  |  | 21 | 6 | 965 | 96.4 | 10.0 |
|  |  |  | 35 | 6 | 2244 | 158.2 | 7.1 |
|  | 5 | Y | 7 | 6 | 183 | 21.0 | 11.5 |
|  |  |  | 21 | 6 | 798 | 81.9 | 10.3 |
|  |  |  | 35 | 6 | 2081 | 145.2 | 7.0 |
|  | 6 | X | 7 | 6 | 183 | 24.7 | 13.5 |
|  |  |  | 21 | 6 | 990 | 159.3 | 16.1 |
|  |  |  | 35 | 6 | 2390 | 172.0 | 7.2 |
|  | 7 | X | 7 | 6 | 197 | 25.0 | 12.7 |
|  |  |  | 21 | 6 | 1068 | 98.6 | 9.2 |
|  |  |  | 35 | 6 | 2661 | 265.4 | 10.0 |
|  | 8 | Y | 7 | 6 | 183 | 35.0 | 19.1 |
|  |  |  | 21 | 6 | 1008 | 159.2 | 15.8 |
|  |  |  | 35 | 6 | 2139 | 234.3 | 11.0 |
|  | 9 | X | 7 | 6 | 180 | 34.5 | 19.1 |
|  |  |  | 21 | 6 | 904 | 93.1 | 10.3 |
|  |  |  | 35 | 6 | 2229 | 249.1 | 11.2 |
|  | 10 | Y | 7 | 6 | 186 | 19.4 | 10.4 |
|  |  |  | 21 | 6 | 798 | 104.4 | 13.1 |
|  |  |  | 35 | 6 | 2143 | 144.9 | 6.8 |
|  | 11 | Y | 7 | 6 | 185 | 10.8 | 5.9 |
|  |  |  | 21 | 6 | 878 | 131.9 | 15.0 |
|  |  |  | 35 | 6 | 2161 | 229.8 | 10.6 |
|  | 12 | Y | 7 | 6 | 183 | 19.4 | 10.6 |
|  |  |  | 21 | 6 | 909 | 124.0 | 13.6 |
|  |  |  | 35 | 6 | 2156 | 333.7 | 15.5 |
|  | 13 | X | 7 | 6 | 190 | 22.4 | 11.8 |
|  |  |  | 21 | 6 | 1013 | 97.9 | 9.7 |
|  |  |  | 35 | 6 | 2562 | 368.6 | 14.4 |
|  | 14 | Y | 7 | 6 | 184 | 16.8 | 9.1 |
|  |  |  | 21 | 6 | 929 | 110.0 | 11.8 |
|  |  |  | 35 | 6 | 2127 | 214.6 | 10.1 |
|  | 15 | X | 7 | 6 | 202 | 28.7 | 14.2 |
|  |  |  | 21 | 6 | 1047 | 75.8 | 7.2 |
|  |  |  | 35 | 6 | 2309 | 111.6 | 4.8 |
|  | 16 | X | 7 | 6 | 199 | 20.3 | 10.2 |
|  |  |  | 21 | 6 | 1048 | 157.4 | 15.0 |
|  |  |  | 35 | 6 | 2585 | 289.4 | 11.2 |
|  | 17 | Y | 7 | 6 | 206 | 28.2 | 13.7 |
|  |  |  | 21 | 6 | 974 | 165.2 | 17.0 |
|  |  |  | 35 | 6 | 2221 | 290.5 | 13.1 |
|  | 18 | X | 7 | 6 | 189 | 35.7 | 18.9 |
|  |  |  | 21 | 6 | 961 | 114.5 | 11.9 |
|  |  |  | 35 | 6 | 2234 | 138.3 | 6.2 |
|  | 19 | Y | 7 | 6 | 184 | 19.7 | 10.7 |
|  |  |  | 21 | 6 | 856 | 61.8 | 7.2 |
|  |  |  | 35 | 6 | 2025 | 231.0 | 11.4 |
|  | 20 | X | 7 | 6 | 198 | 25.4 | 12.8 |
|  |  |  | 21 | 6 | 956 | 135.1 | 14.1 |
|  |  |  | 35 | 6 | 2253 | 108.7 | 4.8 |
|  | 21 | Y | 7 | 6 | 193 | 17.9 | 9.3 |
|  |  |  | 21 | 6 | 889 | 141.5 | 15.9 |
|  |  |  | 35 | 6 | 2213 | 328.2 | 14.8 |
|  | 22 | X | 7 | 6 | 208 | 36.4 | 17.5 |
|  |  |  | 21 | 6 | 989 | 114.6 | 11.6 |
|  |  |  | 35 | 6 | 2481 | 246.4 | 9.9 |
|  | 23 | Y | 7 | 6 | 198 | 21.8 | 11.0 |
|  |  |  | 21 | 6 | 925 | 90.1 | 9.7 |
|  |  |  | 35 | 6 | 2232 | 224.7 | 10.1 |
|  | 24 | Y | 7 | 6 | 185 | 8.8 | 4.8 |
|  |  |  | 21 | 6 | 886 | 92.0 | 10.4 |
|  |  |  | 35 | 6 | 1963 | 137.0 | 7.0 |
| B | 25 | X | 7 | 6 | 197 | 27.0 | 13.7 |
|  |  |  | 21 | 6 | 1010 | 46.2 | 4.6 |
|  |  |  | 35 | 6 | 2162 | 159.4 | 7.4 |
|  | 26 | Y | 7 | 6 | 187 | 16.4 | 8.7 |
|  |  |  | 21 | 6 | 924 | 164.2 | 17.8 |
|  |  |  | 35 | 6 | 2296 | 260.1 | 11.3 |
|  | 27 | X | 7 | 6 | 190 | 27.4 | 14.4 |
|  |  |  | 21 | 6 | 973 | 178.1 | 18.3 |
|  |  |  | 35 | 6 | 2465 | 224.8 | 9.1 |
|  | 28 | Y | 7 | 6 | 187 | 14.1 | 7.6 |
|  |  |  | 21 | 6 | 955 | 102.4 | 10.7 |
|  |  |  | 35 | 6 | 2016 | 189.4 | 9.4 |
|  | 29 | Y | 7 | 6 | 178 | 26.6 | 15.0 |
|  |  |  | 21 | 6 | 951 | 87.2 | 9.2 |
|  |  |  | 35 | 6 | 1988 | 197.4 | 9.9 |
|  | 30 | X | 7 | 6 | 183 | 15.4 | 8.5 |
|  |  |  | 21 | 6 | 1008 | 79.4 | 7.9 |
|  |  |  | 35 | 6 | 2170 | 187.5 | 8.6 |
|  | 31 | X | 7 | 6 | 208 | 26.5 | 12.8 |
|  |  |  | 21 | 6 | 1055 | 91.2 | 8.6 |
|  |  |  | 35 | 6 | 2494 | 340.8 | 13.7 |
|  | 32 | X | 7 | 6 | 204 | 17.1 | 8.4 |
|  |  |  | 21 | 6 | 1019 | 108.7 | 10.7 |
|  |  |  | 35 | 6 | 2380 | 324.4 | 13.6 |
|  | 33 | Y | 7 | 6 | 196 | 21.2 | 10.8 |
|  |  |  | 21 | 6 | 965 | 81.1 | 8.4 |
|  |  |  | 35 | 6 | 2075 | 349.6 | 16.9 |
|  | 34 | Y | 7 | 6 | 190 | 21.7 | 11.4 |
|  |  |  | 21 | 6 | 922 | 49.6 | 5.4 |
|  |  |  | 35 | 6 | 2041 | 170.0 | 8.3 |
|  | 35 | X | 7 | 6 | 207 | 25.4 | 12.2 |
|  |  |  | 21 | 6 | 929 | 70.5 | 7.6 |
|  |  |  | 35 | 6 | 2234 | 227.5 | 10.2 |
|  | 36 | Y | 7 | 6 | 205 | 17.3 | 8.4 |
|  |  |  | 21 | 6 | 987 | 125.1 | 12.7 |
|  |  |  | 35 | 6 | 2488 | 263.0 | 10.6 |
|  | 37 | X | 7 | 6 | 201 | 34.7 | 17.3 |
|  |  |  | 21 | 6 | 1009 | 102.9 | 10.2 |
|  |  |  | 35 | 6 | 2228 | 236.1 | 10.6 |
|  | 38 | X | 7 | 6 | 194 | 26.3 | 13.6 |
|  |  |  | 21 | 6 | 1001 | 34.1 | 3.4 |
|  |  |  | 35 | 6 | 2335 | 164.5 | 7.0 |
|  | 39 | Y | 7 | 6 | 196 | 18.1 | 9.3 |
|  |  |  | 21 | 6 | 972 | 65.4 | 6.7 |
|  |  |  | 35 | 6 | 2138 | 112.1 | 5.3 |
|  | 40 | X | 7 | 6 | 208 | 31.6 | 15.2 |
|  |  |  | 21 | 6 | 953 | 130.7 | 13.7 |
|  |  |  | 35 | 6 | 2511 | 383.6 | 15.3 |
|  | 41 | X | 7 | 6 | 201 | 21.4 | 10.6 |
|  |  |  | 21 | 6 | 1033 | 232.1 | 22.5 |
|  |  |  | 35 | 6 | 2554 | 193.1 | 7.6 |
|  | 42 | Y | 7 | 6 | 200 | 20.7 | 10.4 |
|  |  |  | 21 | 6 | 896 | 116.4 | 13.0 |
|  |  |  | 35 | 6 | 2370 | 276.1 | 11.7 |
|  | 43 | Y | 7 | 6 | 204 | 24.6 | 12.0 |
|  |  |  | 21 | 6 | 968 | 75.3 | 7.8 |
|  |  |  | 35 | 6 | 2288 | 217.9 | 9.5 |
|  | 44 | Y | 7 | 6 | 203 | 11.8 | 5.8 |
|  |  |  | 21 | 6 | 964 | 93.3 | 9.7 |
|  |  |  | 35 | 6 | 2432 | 231.4 | 9.5 |
|  | 45 | Y | 7 | 6 | 190 | 19.3 | 10.1 |
|  |  |  | 21 | 6 | 903 | 19.1 | 2.1 |
|  |  |  | 35 | 6 | 2023 | 235.1 | 11.6 |
|  | 46 | Y | 7 | 6 | 201 | 29.6 | 14.8 |
|  |  |  | 21 | 6 | 895 | 100.6 | 11.2 |
|  |  |  | 35 | 6 | 2039 | 278.5 | 13.7 |
|  | 47 | X | 7 | 6 | 200 | 20.0 | 10.0 |
|  |  |  | 21 | 6 | 1034 | 88.0 | 8.5 |
|  |  |  | 35 | 6 | 2157 | 261.7 | 12.1 |
|  | 48 | X | 7 | 6 | 216 | 25.3 | 11.7 |
|  |  |  | 21 | 6 | 1023 | 124.3 | 12.2 |
|  |  |  | 35 | 6 | 2643 | 232.1 | 8.8 |
| C | 49 | X | 7 | 6 | 187 | 25.8 | 13.8 |
|  |  |  | 21 | 6 | 1078 | 120.6 | 11.2 |
|  |  |  | 35 | 6 | 2327 | 282.2 | 12.1 |
|  | 50 | X | 7 | 6 | 172 | 28.4 | 16.5 |
|  |  |  | 21 | 6 | 982 | 112.3 | 11.4 |
|  |  |  | 35 | 6 | 2031 | 80.5 | 4.0 |
|  | 51 | X | 7 | 6 | 181 | 15.4 | 8.5 |
|  |  |  | 21 | 6 | 973 | 100.5 | 10.3 |
|  |  |  | 35 | 6 | 1993 | 316.6 | 15.9 |
|  | 52 | Y | 7 | 6 | 178 | 15.2 | 8.5 |
|  |  |  | 21 | 6 | 873 | 85.6 | 9.8 |
|  |  |  | 35 | 6 | 1647 | 242.5 | 14.7 |
|  | 53 | X | 7 | 6 | 183 | 24.4 | 13.3 |
|  |  |  | 21 | 6 | 954 | 98.4 | 10.3 |
|  |  |  | 35 | 6 | 2020 | 184.6 | 9.1 |
|  | 54 | Y | 7 | 6 | 170 | 12.3 | 7.2 |
|  |  |  | 21 | 6 | 764 | 70.2 | 9.2 |
|  |  |  | 35 | 6 | 1798 | 334.3 | 18.6 |
|  | 55 | Y | 7 | 6 | 180 | 18.5 | 10.3 |
|  |  |  | 21 | 6 | 838 | 141.2 | 16.9 |
|  |  |  | 35 | 6 | 1546 | 159.3 | 10.3 |
|  | 56 | Y | 7 | 6 | 179 | 25.0 | 14.0 |
|  |  |  | 21 | 6 | 859 | 87.1 | 10.2 |
|  |  |  | 35 | 6 | 1699 | 285.5 | 16.8 |
|  | 57 | Y | 7 | 6 | 177 | 19.9 | 11.2 |
|  |  |  | 21 | 6 | 811 | 136.7 | 16.9 |
|  |  |  | 35 | 6 | 1839 | 215.9 | 11.7 |
|  | 58 | Y | 7 | 6 | 184 | 20.4 | 11.1 |
|  |  |  | 21 | 6 | 808 | 105.0 | 13.0 |
|  |  |  | 35 | 6 | 1523 | 130.4 | 8.6 |
|  | 59 | X | 7 | 6 | 199 | 22.2 | 11.2 |
|  |  |  | 21 | 6 | 998 | 98.8 | 9.9 |
|  |  |  | 35 | 6 | 1974 | 105.9 | 5.4 |
|  | 60 | X | 7 | 6 | 186 | 22.1 | 11.9 |
|  |  |  | 21 | 6 | 983 | 124.6 | 12.7 |
|  |  |  | 35 | 6 | 2097 | 360.4 | 17.2 |
|  | 61 | X | 7 | 6 | 184 | 22.8 | 12.4 |
|  |  |  | 21 | 6 | 942 | 58.7 | 6.2 |
|  |  |  | 35 | 6 | 2185 | 252.1 | 11.5 |
|  | 62 | X | 7 | 6 | 176 | 18.5 | 10.5 |
|  |  |  | 21 | 6 | 917 | 81.0 | 8.8 |
|  |  |  | 35 | 6 | 1882 | 133.6 | 7.1 |
|  | 63 | X | 7 | 6 | 189 | 12.1 | 6.4 |
|  |  |  | 21 | 6 | 934 | 114.6 | 12.3 |
|  |  |  | 35 | 6 | 2157 | 297.1 | 13.8 |
|  | 64 | Y | 7 | 6 | 181 | 24.2 | 13.4 |
|  |  |  | 21 | 6 | 797 | 76.7 | 9.6 |
|  |  |  | 35 | 6 | 1653 | 211.0 | 12.8 |
|  | 65 | X | 7 | 6 | 211 | 32.7 | 15.5 |
|  |  |  | 21 | 6 | 1087 | 126.9 | 11.7 |
|  |  |  | 35 | 6 | 2017 | 245.4 | 12.2 |
|  | 66 | Y | 7 | 6 | 177 | 18.4 | 10.4 |
|  |  |  | 21 | 6 | 838 | 161.6 | 19.3 |
|  |  |  | 35 | 6 | 1855 | 178.7 | 9.6 |
|  | 67 | Y | 7 | 6 | 188 | 7.5 | 4.0 |
|  |  |  | 21 | 6 | 871 | 140.7 | 16.1 |
|  |  |  | 35 | 6 | 1960 | 179.2 | 9.1 |
|  | 68 | X | 7 | 6 | 191 | 16.0 | 8.4 |
|  |  |  | 21 | 6 | 932 | 53.9 | 5.8 |
|  |  |  | 35 | 6 | 2003 | 133.1 | 6.6 |
|  | 69 | Y | 7 | 6 | 195 | 26.9 | 13.8 |
|  |  |  | 21 | 6 | 1004 | 70.2 | 7.0 |
|  |  |  | 35 | 6 | 2077 | 343.2 | 16.5 |
|  | 70 | Y | 7 | 6 | 180 | 9.2 | 5.1 |
|  |  |  | 21 | 6 | 846 | 88.7 | 10.5 |
|  |  |  | 35 | 6 | 1759 | 289.2 | 16.4 |
|  | 71 | X | 7 | 6 | 207 | 17.9 | 8.7 |
|  |  |  | 21 | 6 | 1044 | 55.4 | 5.3 |
|  |  |  | 35 | 6 | 2239 | 177.5 | 7.9 |
|  | 72 | Y | 7 | 6 | 198 | 25.5 | 12.9 |
|  |  |  | 21 | 6 | 779 | 66.2 | 8.5 |
|  |  |  | 35 | 6 | 1792 | 263.3 | 14.7 |
| All | | | | 1296 | 1096.4 | 835.3 | 76.2 |

^1^Values are presented as means. SD: Standard deviation; CV: coefficient of variation. BW: Body weight.

BD: Basal diet; PR: Basal diet + probiotic; PH: Basal diet + phytobiotic. F: female; M: male. X and Y correspond to two fast growing commercial poultry genetic lines.
